# Safety of treating acute pulmonary embolism at home: an individual patient data meta-analysis

**DOI:** 10.1093/eurheartj/ehae378

**Published:** 2024-07-12

**Authors:** Dieuwke Luijten, Delphine Douillet, Kim Luijken, Cecile Tromeur, Andrea Penaloza, Olivier Hugli, Drahomir Aujesky, Stefano Barco, Joseph R Bledsoe, Kyle E Chang, Francis Couturaud, Paul L den Exter, Carme Font, Menno V Huisman, David Jimenez, Christopher Kabrhel, Jeffrey A Kline, Stavros Konstantinides, Thijs van Mens, Remedios Otero, W Frank Peacock, Olivier Sanchez, William B Stubblefield, Luca Valerio, David R Vinson, Philip Wells, Maarten van Smeden, Pierre-Marie Roy, Frederikus A Klok

**Affiliations:** Department of Medicine—Thrombosis and Hemostasis, Leiden University Medical Center, Albinusdreef 2, 2333 ZA Leiden, The Netherlands; Emergency Department, CHU Angers, Angers, France; UNIV Angers, UMR MITOVASC INSERM 1083—CNRS 6015, Equipe CARME, SFR ICAT, Angers, France; F-CRIN, INNOVTE, Saint-Etienne, France; Department of Epidemiology, Julius Center for Health Sciences and Primary Care, University Medical Center Utrecht, Utrecht University, Utrecht, The Netherlands; F-CRIN, INNOVTE, Saint-Etienne, France; Département de Médecine Interne et Pneumologie, Université de Bretagne Occidentale, INSERM U1304-GETBO, Centre Hospitalo-Universitaire de Brest, F 29200 Brest, France; F-CRIN, INNOVTE, Saint-Etienne, France; Emergency Department, Cliniques Universitaires Saint-Luc, Brussels, Belgium; UCLouvain, Brussels, Belgium; Emergency Department, University Hospital of Lausanne and University of Lausanne, Lausanne, Switzerland; Department of General Internal Medicine, Inselspital, Bern University Hospital, University of Bern, Bern, Switzerland; Center for Thrombosis and Hemostasis, University Medical Center of the Johannes Gutenberg University, Mainz, Germany; Department of Angiology, University Hospital Zurich, Zurich, Switzerland; Department of Emergency Medicine Intermountain Healthcare, Salt Lake City, UT, USA; Center for Vascular Emergencies, Department of Emergency Medicine, Massachusetts General Hospital, Harvard Medical School, Boston, MA, USA; School of Medicine, California University of Science and Medicine, Colton, CA, USA; F-CRIN, INNOVTE, Saint-Etienne, France; Département de Médecine Interne et Pneumologie, Université de Bretagne Occidentale, INSERM U1304-GETBO, Centre Hospitalo-Universitaire de Brest, F 29200 Brest, France; Department of Medicine—Thrombosis and Hemostasis, Leiden University Medical Center, Albinusdreef 2, 2333 ZA Leiden, The Netherlands; Medical Oncology Department, Hospital Clinic de Barcelona, Barcelona, Spain; Department of Medicine—Thrombosis and Hemostasis, Leiden University Medical Center, Albinusdreef 2, 2333 ZA Leiden, The Netherlands; Respiratory Department and Medicine Department, Ramon y Cajal Hospital and Alcalá University (IRYCIS), CIBER de Enfermedades Respiratorias (CIBERES), Madrid, Spain; Center for Vascular Emergencies, Department of Emergency Medicine, Massachusetts General Hospital, Harvard Medical School, Boston, MA, USA; Department of Emergency Medicine, Wayne State University School of Medicine, Detroit, MI, USA; Center for Thrombosis and Hemostasis, University Medical Center of the Johannes Gutenberg University, Mainz, Germany; Department of Cardiology, Democritus University of Thrace, Alexandroupolis, Greece; Department of Medicine—Thrombosis and Hemostasis, Leiden University Medical Center, Albinusdreef 2, 2333 ZA Leiden, The Netherlands; Pneumology Department, Hospital Universitario Virgen del Rocío-IBIS-US_CIBERES, Seville, Spain; Department of Emergency Medicine, Baylor College of Medicine, Ben Taub General Hospital, Houston, TX, USA; F-CRIN, INNOVTE, Saint-Etienne, France; University Paris Cité, INSERM UMR-S 1140 Innovative Therapies in Haemostasis, Paris, France; Pneumology Department and Intensive Care, Hôpital Européen Georges Pompidou, APHP, Paris, France; Department of Emergency Medicine, Vanderbilt University Medical Center, Nashville, TN, USA; Center for Thrombosis and Hemostasis, University Medical Center of the Johannes Gutenberg University, Mainz, Germany; Department of Cardiology, University Medical Center of the Johannes Gutenberg University, Mainz, Germany; The Permanente Medical Group, Oakland, CA, USA; Delivery Science and Applied Research Program, Kaiser Permanente Division of Research, Oakland, CA, USA; The Kaiser Permanente CREST Network, Oakland, CA, USA; Department of Emergency Medicine, Kaiser Permanente Roseville Medical Center, Roseville, CA, USA; Department of Medicine, University of Ottawa, Ottawa, ON, Canada; Department of Epidemiology, Julius Center for Health Sciences and Primary Care, University Medical Center Utrecht, Utrecht University, Utrecht, The Netherlands; Emergency Department, CHU Angers, Angers, France; UNIV Angers, UMR MITOVASC INSERM 1083—CNRS 6015, Equipe CARME, SFR ICAT, Angers, France; F-CRIN, INNOVTE, Saint-Etienne, France; Department of Medicine—Thrombosis and Hemostasis, Leiden University Medical Center, Albinusdreef 2, 2333 ZA Leiden, The Netherlands; Center for Thrombosis and Hemostasis, University Medical Center of the Johannes Gutenberg University, Mainz, Germany

**Keywords:** Pulmonary embolism, Emergency care, Outpatient care, Clinical decision-making, Early discharge

## Abstract

**Background and Aims:**

Home treatment is considered safe in acute pulmonary embolism (PE) patients selected by a validated triage tool (e.g. simplified PE severity index score or Hestia rule), but there is uncertainty regarding the applicability in underrepresented subgroups. The aim was to evaluate the safety of home treatment by performing an individual patient-level data meta-analysis.

**Methods:**

Ten prospective cohort studies or randomized controlled trials were identified in a systematic search, totalling 2694 PE patients treated at home (discharged within 24 h) and identified by a predefined triage tool. The 14- and 30-day incidences of all-cause mortality and adverse events (combined endpoint of recurrent venous thromboembolism, major bleeding, and/or all-cause mortality) were evaluated. The relative risk (RR) for 14- and 30-day mortalities and adverse events is calculated in subgroups using a random effects model.

**Results:**

The 14- and 30-day mortalities were 0.11% [95% confidence interval (CI) 0.0–0.24, *I*^2^ = 0) and 0.30% (95% CI 0.09–0.51, *I*^2^ = 0). The 14- and 30-day incidences of adverse events were 0.56% (95% CI 0.28–0.84, *I*^2^ = 0) and 1.2% (95% CI 0.79–1.6, *I*^2^ = 0). Cancer was associated with increased 30-day mortality [RR 4.9; 95% prediction interval (PI) 2.7–9.1; *I*^2^ = 0]. Pre-existing cardiopulmonary disease, abnormal troponin, and abnormal (N-terminal pro–)B-type natriuretic peptide [(NT-pro)BNP] at presentation were associated with an increased incidence of 14-day adverse events [RR 3.5 (95% PI 1.5–7.9, *I*^2^ = 0), 2.5 (95% PI 1.3–4.9, *I*^2^ = 0), and 3.9 (95% PI 1.6–9.8, *I*^2^ = 0), respectively], but not mortality. At 30 days, cancer, abnormal troponin, and abnormal (NT-pro)BNP were associated with an increased incidence of adverse events [RR 2.7 (95% PI 1.4–5.2, *I*^2^ = 0), 2.9 (95% PI 1.5–5.7, *I*^2^ = 0), and 3.3 (95% PI 1.6–7.1, *I*^2^ = 0), respectively].

**Conclusions:**

The incidence of adverse events in home-treated PE patients, selected by a validated triage tool, was very low. Patients with cancer had a three- to five-fold higher incidence of adverse events and death. Patients with increased troponin or (NT-pro)BNP had a three-fold higher risk of adverse events, driven by recurrent venous thromboembolism and bleeding.

## Structured Graphical Abstract

**Structured Graphical Abstract:**
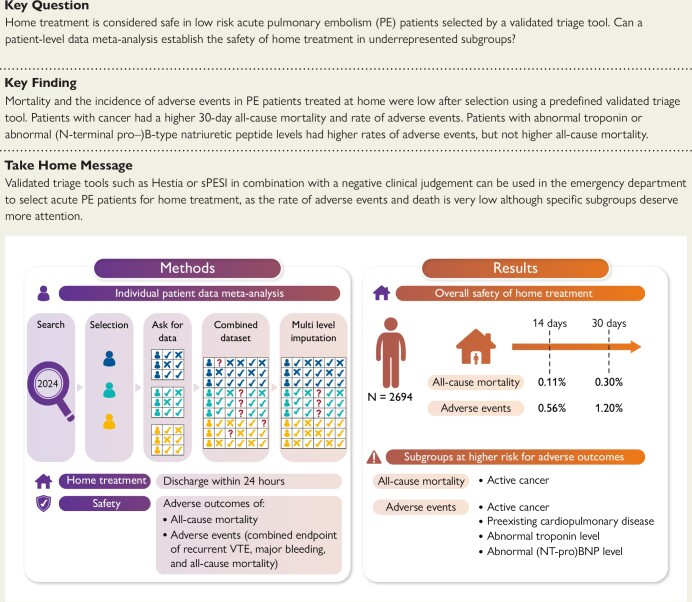
Safety of home treatment of acute pulmonary embolism in the overall population and clinically relevant patient subgroups. (NT-pro)BNP, (N-terminal pro–)B-type natriuretic peptide; PE, pulmonary embolism; VTE, venous thromboembolism.

**See the editorial comment for this article ‘Triaging early discharge for pulmonary embolism: home is where the heart(/lung) is’, by K.T. Kadakia and B. Bikdeli, https://doi.org/10.1093/eurheartj/ehae358.**

## Introduction

Acute pulmonary embolism (PE) has a broad spectrum of clinical presentations.^1,2^ Haemodynamically unstable patients as well as stable patients with an elevated risk of deterioration due to obstructive shock or respiratory failure should be hospitalized and closely monitored, while others might be eligible for immediate discharge and home treatment. As home treatment is associated with high patient satisfaction and lower healthcare costs, identification of acute PE patients with no medical contraindication to home treatment is relevant for both individuals, local hospital governance, and society.^3–5^

The PE severity index (PESI) and the simplified PESI (sPESI) are clinical prognostic models estimating the absolute 30-day mortality.^6–8^ The Hestia rule consists of a checklist of 11 indications to hospitalize PE patients (*Table 1*).^9,10^ Strategies based on either of these triage tools are proven safe to select PE patients eligible for home treatment, with low rates of adverse events.^8–11^

**Table 1:** Hestia rule, pulmonary embolism severity index, and simplified pulmonary embolism severity index

Hestia^9,10^	Answer	PESI^6^	Points	sPESI^7^	Points
Is the patient haemodynamically unstable?^a^	Yes/no	Age	Years	Age > 80 years	1
Is thrombolysis or embolectomy necessary?	Yes/no	Male sex	+10	History of cancer	1
Active bleeding or high risk of bleeding?^b^	Yes/no	History of cancer	+30	Chronic cardiopulmonary disease	1
>24 h of oxygen supply to maintain oxygen saturation > 90%?	Yes/no	History of heart failure	+10	Systolic blood pressure < 100 mmHg	1
Is pulmonary embolism diagnosed during anticoagulant treatment?	Yes/no	History of chronic lung disease	+10	Heart rate ≥ 110 b.p.m.	1
Severe pain needing intravenous pain medication for >24 h?	Yes/no	Heart rate ≥ 110 b.p.m.	+20	Arterial oxygen saturation < 90%	1
Medical or social reason for treatment in the hospital for >24 h (infection, malignancy, no support system)^c^?	Yes/no	Systolic blood pressure < 100 mmHg	+30		
Does the patient have a creatinine clearance of <30 mL/min?^d^	Yes/no	Respiratory rate ≥ 30/min	+20		
Does the patient have severe liver impairment?^e^	Yes/no	Temperature < 36°C/96.8°F	+20		
Is the patient pregnant?	Yes/no	Altered mental status (disorientation, lethargy, stupor, or coma)	+60		
Does the patient have a documented history of heparin-induced thrombocytopenia?	Yes/no	Arterial oxygen saturation < 90%	+20		
If all questions can be answered with ‘No’, the patient has a negative Hestia rule and is eligible for home treatment		If the PESI Class is I (total score of 0–65) or II (total score of 66–85), a patient is eligible for home treatment		If the sPESI = 0, a patient is eligible for home treatment	

PESI, pulmonary embolism severity index; sPESI, simplified pulmonary embolism severity index.

^a^Include the following criteria, but leave these to the discretion of the investigator: systolic blood pressure < 100 mmHg with heart rate > 100 b.p.m.; condition requiring admission to an intensive care unit.

^b^Gastrointestinal bleeding in the preceding 14 days, recent stroke (<4 weeks ago), recent operation (<2 weeks ago), bleeding disorder or thrombocytopenia (platelet count <75 × 10^9^/L), and uncontrolled hypertension (systolic blood pressure > 180 mmHg or diastolic blood pressure > 110 mmHg).

^c^This subjective item allows to hospitalize patients based on medical or social reasons needing hospitalization. However, since it is a subjective item, interpretation on when a patient requires hospitalization based on this item can very. For example, not all patients with active cancer were assessed to require hospitalization based on their malignancy and thus received home treatment in the original studies.

^d^Calculated creatinine clearance according to the Cockcroft–Gault formula.

^e^Left to the discretion of the physician.

However, most studies evaluating the safety of home treatment included relatively low numbers of patients and were conducted in single centres, resulting in broad confidence intervals (CIs) around the incidences of adverse outcomes. Moreover, specific patient subgroups, e.g. those with cancer, serious comorbidities, or intermediate-risk PE, were underrepresented or even excluded, fuelling discussion on the applicability of the trial results to these groups.^12–14^

We performed a systematic review and individual patient data meta-analysis (IPDMA) to estimate the overall incidence of adverse events in patients with acute PE who received home treatment and were selected using validated triage tools. We aimed to estimate incidences of adverse events in predefined clinically relevant patient subgroups.

## Methods

### Search strategy and selection criteria

We conducted a systematic literature search up to January 2024 for all relevant publications in PubMed, Embase, Web of Science, Cochrane Library, EmCare, Academic Search Premier, the WHO COVID-19 Research Database, and Google Scholar (see Supplementary data online, *Appendix A*). Relevant publications were independently assessed for eligibility in duplicate by four individual authors (D.L., D.D., C.T., and F.A.K.). Discrepancies were resolved by discussion. Study designs eligible for inclusion were (i) prospective cohort studies or randomized controlled trials investigating different algorithms to assess eligibility for home treatment, with (ii) established acute symptomatic or incidental acute PE patients involving sub-segmental or more proximal pulmonary arteries confirmed by computed tomography pulmonary angiography (CTPA) or a high-probability ventilation/perfusion (VQ) imaging, (iii) who were managed according to a predefined algorithm determining initiation of initial treatment as in- or outpatient, (iv) with a minimum follow-up duration of 1 month, (v) reporting at least one of the predefined outcomes, and (vi) including a minimum of 50 patients treated at home.

Lead investigators of the included studies were invited to provide de-identified individual patient data (IPD) of patients who received home treatment upon diagnosis. Patients with a PE diagnosis during hospitalization (>48 h) were excluded from this study. Individual patient information was collected, including demographics, risk factors for venous thromboembolism (VTE), comorbidities, items for evaluation of PE severity [e.g. vital signs, laboratory results, and presence of right ventricular (RV) overload and/or dysfunction], and time until discharge from the hospital (see Supplementary data online, *Appendix B*). All available data on the occurrence of recurrent VTE, bleeding complications, mortality, and loss to follow-up according to the pre-specified definitions from the protocol were collected. Data from the original studies were converted to a universal database either by the primary researcher of the original study or by the lead investigator of this IPDMA. Correctness of conversion was performed by repeating analysis of the original studies in the new data set to identify non-matching results.

Risk of bias was evaluated using a modified version of the Newcastle–Ottawa Scale (NOS) for observational studies.^15^ For the risk of bias analysis, each arm of a randomized trial was considered an independent observational cohort. Studies were eligible to be awarded a maximum of three stars for quality of patient selection, as well as for outcome assessment. A study was considered at low risk of bias when achieving three stars in selection and two or three stars in outcome, at moderate risk of bias with two stars in selection and two or three stars in outcome, and at high risk of bias with zero or one star in selection or zero or one star in outcome. The evaluation of the risk of bias was independently performed by two researchers (D.D. and D.L.), and disagreements were resolved by discussion or by consultation of a third researcher (F.A.K.) if the two researchers could not agree.

### Outcomes

**Figure 1:**
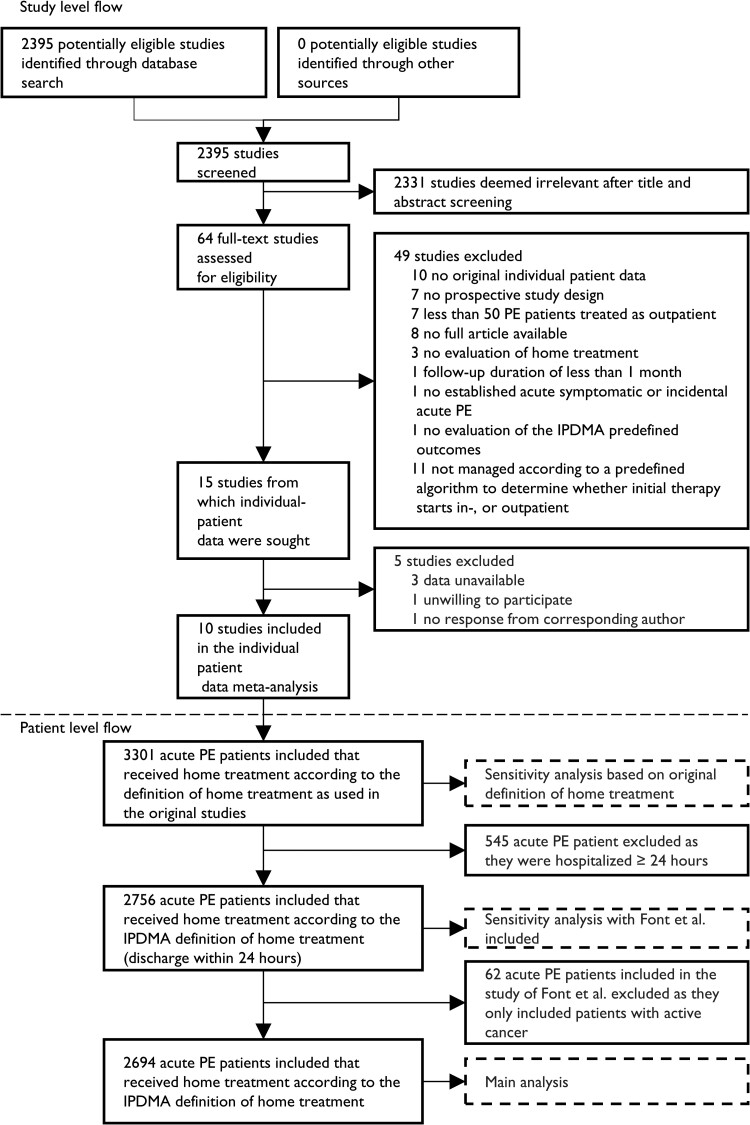
Flowchart of included studies. Above the dashed line is the study flowchart on study level. We included 10 studies in our IPDMA. Below the dashed line is the study flowchart on patient-level data. The main analysis was performed only with patients who were discharged within 24 h. IPDMA, individual patient data meta-analysis; PE, pulmonary embolism

Our primary aim was to evaluate the safety of home treatment in the overall population by calculating the 14-day incidence of all-cause mortality and adverse events (i.e. a combined endpoint of recurrent VTE, major bleeding, and all-cause mortality). We defined home treatment as discharge from the hospital within 24 h after diagnosis of PE, randomization, or emergency department registration; this meant that patients who were hospitalized for >24 h were excluded from our main analysis (*Figure 1*). We also evaluated other adverse outcomes: (i) 30-day incidence of all-cause mortality and of adverse events, (ii) 14- and 30-day incidences of recurrent VTE, and (iii) 14- and 30-day incidences of major bleeding.^16^

The secondary aims of this study were to evaluate all-cause mortality and adverse outcomes in relevant patient subgroups. The following predefined subgroups were evaluated based on the presence or absence of the following characteristics: symptomatic vs. incidental PE, cancer, decreased kidney function, pre-existing cardiopulmonary disease, abnormal (N-terminal pro–)B-type natriuretic peptide [(NT-pro)BNP], abnormal troponin, RV overload, RV dysfunction, and the applied triage tool (i.e. Hestia or sPESI/PESI). Definitions of these subgroups are described in Supplementary data online, *Appendix C*. Cancer was considered active if meeting at least one of the following criteria: (i) current diagnosis of cancer, (ii) receiving treatment for cancer, or (iii) not receiving treatment for cancer and not in complete remission (e.g. palliative patients).^17^

### Statistical analysis

Baseline characteristics were described using median and interquartile range (IQR) or mean and standard deviation (SD) for continuous variables and counts and proportions for categorical variables.

Data included in our analysis were missing with proportions ranging from 1% to 62% (see Supplementary data online, *Appendix D*; *Table S1*).

Values non-completely missing were handled using multiple imputations by chained equations with a fixed effects approach, taking study into account as a cluster variable using the mice package (see Supplementary data online, *Appendix E*).^18,19^ Using fully conditional specifications, we defined an imputation model containing all subgroup variables and the outcomes at 14 days for imputation and added auxiliary variables to improve imputation. The number of imputed data sets was 75, and the number of iterations per imputation was 50. When values were completely missing in a study (i.e. a variable was 100% missing within a certain study), missing variables were not handled using imputations; these variables remained missing for all individuals derived from that study (see Supplementary data online, *Appendix D*; *Table S1*). Individuals with missing subgroup or outcome data were excluded from the corresponding analysis after imputation.^20,21^

Overall and for each subgroup, the incidence of each safety measure was calculated as a proportion at the corresponding prediction time point averaged over the included studies (i.e. using a fixed effects approach). Proportion and standard error were calculated across imputed data sets using Rubin’s rules, and 95% CI were computed by a Wald interval.^22^

We calculated the relative risk (RR) for adverse events when a subgroup characteristic was present vs. absent. Relative risks were estimated in each study using a penalized log-binomial model with the subgroup variable as the only independent variable and calculated over imputed data sets using Rubin’s rules to arrive at an estimate of the RR for each study.^22^ Single value studies (e.g. subgroup characteristic was present in all patients or absent in all patients; Supplementary data online, *Appendix D;Table S1*) were excluded from this analysis. Due to very low event fractions across studies and even zero events in some cases, Firth’s correction was applied using the brglm2 package.^23,24^ To arrive at an overall RR across studies, we subsequently used a random effects model with restricted maximum likelihood estimation to derive prediction intervals (PIs).

For the evaluation of specific triage tools to assess eligibility for home treatment, studies were only included in the subgroup strategy of the tool that was originally used in the study to assess eligibility. Subsequently incidence of adverse events was calculated with a corresponding 95% CI for each tool. No direct statistical comparison across different tools was performed due to the methodological challenge of comparing outcomes across distinct study designs and populations, emphasizing the descriptive nature of this sub-analysis.

We performed three sensitivity analyses. First, the definition of home treatment in the studies, e.g. Barco *et al*.^25^ (discharge within 48 h) and Otero *et al.*^26^ (discharge within 72–120 h), varied from our IPDMA definition of home treatment. We performed a sensitivity analysis that included all patients who did not meet the IPDMA definition of home treatment of discharge within 24 h (excluded from main analysis) but were treated at home according to the definition of home treatment of the original study (*Figure 1*). Second, as Font *et al.*^27^ included only patients with cancer, this study may not be an accurate representation of low-risk acute PE patients who received home treatment and was therefore excluded from the main analysis. However, to maximize the utilization of available data and ensure that the valuable information that these patients hold contributed to a comprehensive assessment of home treatment safety across different patient profiles, we performed a sensitivity analysis for the overall safety by including the study by Font *et al.*^27^ Finally, as we used multiple imputations to handle missing data, but as we did not have exact information on how each variable was collected in a data set, we cannot guarantee that missing values were truly missing at random, potentially influencing the imputation model. We therefore performed a sensitivity analysis of the overall safety based on the non-imputed complete case data. The sensitivity analyses were performed to explore robustness of our results and not to establish statistical significance compared with the main analysis. Therefore, no significance tests were performed as part of this analysis.

All analyses were performed using R, version 4.3.1 (R Foundation for Statistical Computing, Vienna, Austria; www.R-project.org).

## Results

### Included studies

The literature search resulted in 2395 studies, of which 64 full texts were screened for eligibility. Fifteen studies met the predefined inclusion and none for the exclusion criteria. Their corresponding authors were contacted with a request to share de-identified IPD. Data of 10 studies were shared and included in our study (*Figure 1*). Nine studies had a low risk of bias and one study a moderate risk of bias^27^ (potential selection bias as only patients with cancer were included; Supplementary data online, *Appendix D* and *Table S2*). As Font *et al.*^27^ included only patients with cancer, this study may not be an accurate representation of low-risk acute PE patients who received home treatment and was therefore excluded from the main analysis. Characteristics of the included studies are summarized in *Table 2*.^3,9–11,25–31^ There were no important issues when checking the IPD.

**Table 2:** Characteristics of included studies

Study	Primary study aim	Study period	Selection of low-risk patients	Follow-up (days)	Outcome adjudication	PE patients treated at home according to the study definition of home treatment, *n* (% of total study population)	PE patients treated at home according to the IPDMA definition of home treatment, *n* (% of previous column)
Barco *et al.*^25^ (HoT-PE)	Evaluate the efficacy and safety of home treatment in low-risk acute PE patient treated with rivaroxaban	May 2014–June 2018	Negative Hestia rule and no RV dysfunction or intra-cardiac thrombi	90	Yes	520 (100)^a^	170 (32)^i^
Bledsoe *et al.*^3^	Evaluate the efficacy and safety outpatient treatment in low-risk acute PE patients	January 2013–October 2016	PESI Class I or II with a list of exclusion criteria including RV dysfunction on echocardiography	90	No	200 (100)^b^	192 (96)^i^
den Exter et al.^28^ (Vesta)	Evaluate the utility and safety of the Hestia rule vs. Hestia rule in combination with (NT-pro)BNP testing for selection of outpatient PE treatment	December 2010–February 2014	Negative Hestia rule with/without negative (NT-pro)BNP testing	90	Yes	513 (93)^c^	513 (100)^j^
Font *et al.*^27^	Evaluate the feasibility of outpatient treatment in patients with cancer and PE	May 2006–December 2009	Negative Hestia-like criteria: systolic blood pressure < 100 mmHg, arterial oxygen pressure < 60 mmHg or pulse oximetry < 90%, active bleeding, platelet count ≥50 000/mm^3^, renal failure, lack of social support, poor treatment compliance, or the presence of other admission criteria according to treating physicians	90	Yes	62 (45)^d^	62 (100)^d^
Kabrhel *et al.*^29^	Determine whether a protocol combining risk stratification, treatment with rivaroxaban, and defined follow-up is associated with a greater proportion of patients with VTE treated as outpatients	September 2015–unknown	Negative: social or psychological barrier, abnormal vital signs, coronary artery disease or congestive heart failure, elevated troponin, high risk of bleeding, large PE, intermediate PE with RV dysfunction, high-risk DVT	28	No	164 (25)^e^	122 (74)^k^
Kline *et al.*^30^ (MATH-VTE)	Evaluate the safety and efficacy of home treatment with a DOAC in low-risk acute PE patient presented at the emergency department	April 2016–March 2019	Negative modified Hestia rule or sPESI of 0 plus physician’s judgement	30	No	604 (100)^f^	604 (100)^f^
Otero *et al.*^26^	Evaluate efficacy and safety of early discharge in patients with low risk according to a clinical prediction rule	February 2005–April 2007	≤2 points on a clinical prediction score, haemodynamic instability, troponin ≥ 0.1 ng/mL, saturation < 93%, need for hospitalization for comorbidities, severe chronic obstructive pulmonary disease, severe asthma, active or high risk of bleeding, pregnancy, morbid obesity, or RV dysfunction	30	No	72 (55)^g^	0 (0)^j^
Roy *et al.*^11^ (HOME-PE)	Evaluate safety and efficacy of Hestia vs. sPESI in the selection of outpatient PE treatment	January 2017–July 2019	Negative Hestia rule or sPESI of 0 (overruling by clinician possible)	30	Yes	739 (38)^h^	681 (92)^k^
Vinson *et al.*^31^ (eSPEED)	Evaluate the effect of an integrated electronic clinical decision support system to facilitate risk stratification and decision for selection of outpatient PE treatment	January 2014–April 2015	PESI Class I or II with a broad list of relative contraindications from variables from the Ottawa and Hestia exclusion criteria (including absence of RV dysfunction)	30	No	130 (19)^c^	116 (89)^k^
Zondag *et al.*^9,10^ (Hestia)	Evaluate the efficacy and safety of outpatient treatment according to the Hestia rule in patients with acute PE	May 2008–April 2010	Negative Hestia rule	90	Yes	297 (100)^c^	296 (100)^j^

Since follow-up was only 28 days in the study by Kabrhel et al., we used 28 days as a surrogate measurement for incidence within 30 days for Kabrhel et al.^29^

DOAC, direct oral anticoagulant; DVT, deep vein thrombosis; (NT-pro)BNP, N-terminal pro–B-type natriuretic peptide; PE, pulmonary embolism; PESI, pulmonary embolism severity index; RV, right ventricle; sPESI, simplified pulmonary embolism severity index; VTE, venous thromboembolism.

^a^Discharge from the hospital within 48 h of presentation.

^b^Observation or hospitalization for >12–<24 h.

^c^Discharge from the hospital within 24 h of PE diagnosis.

^d^Discharge within 12 h after PE diagnosis.

^e^Discharged directly from the ED or admitted to the ED observation unit with a plan for discharge.

^f^Discharge within 24 h after triage.

^g^Discharge at 72 or 120 h (61 and 39%, respectively).

^h^Discharge within 24 h of presentation or randomization.

^i^Within 24 h after randomization/inclusion.

^j^Within 24 h after acute PE diagnosis.

^k^Within 24 h after ED presentation.

### Patients

A total of 3301 acute PE patients received home treatment, according to the definition of home treatment in the original studies. Of these, 2756 (83%) were discharged within 24 h. Excluding Font *et al.*^27^ resulted in a total of 2694 acute PE patients discharged within 24 h (*Figure 1*). The following triage tools were used in the studies to assess eligibility for home treatment: (i) Hestia rule (none of the 11 items present; with/without RV overload/dysfunction), (ii) sPESI (0 points) or PESI (Classes I–II) in combination with clinical judgement (with/without RV overload/dysfunction), or (iii) a list of inclusion and exclusion criteria predefined to select eligible patients for home treatment not based on Hestia/sPESI. The characteristics after imputation of patients discharged within 24 h are depicted in *Table 3*.

**Table 3:** Characteristics of patients that received home treatment (defined as discharge within 24 h)

Study, year (reference)	Overall	Zondag *et al.*^10^	den Exter *et al.*^28^	Roy *et al.*^11^	Kline *et al.*^30^	Barco *et al.*^25^	Kabrhel *et al.*^29^	Vinson *et al.*^31^	Bledsoe *et al.*^3^
Patients, *n*	2694	296	513	681	604	170	122	116	192
Age, years, mean (SD)	53.79 (16.06)	54.5 (15.4)	53.5 (14.7)	56.4 (16.2)	52.0 (16.6)	54.5 (16.0)	55.4 (16.4)	60.3 (15.1)	44.4 (14.3)
Female sex, *n* (%)	1282 (48%)	124 (42%)	235 (46%)	314 (46%)	301 (50%)	80 (47%)	64 (52%)	61 (53%)	103 (54%)
Triage tool applied, *n* (%)									
Negative Hestia (or Hestia-like) rule	1623 (60%)	296 (100%)	513 (100%)	351 (51%)	463 (77%)	0 (0%)	0 (0%)	0 (0%)	0 (0%)
sPESI 0 or PESI I/II^a^	471 (17%)	0 (0%)	0 (0%)	330 (49%)	141 (23%)	0 (0%)	0 (0%)	0 (0%)	0 (0%)
sPESI 0 or PESI I/II and absence of RVD^a^	308 (11%)	0 (0%)	0 (0%)	0 (0%)	0 (0%)	0 (0%)	0 (0%)	116 (100%)	192 (100%)
Negative Hestia (or Hestia-like) criteria and absence of RVD	170 (6.3%)	0 (0%)	0 (0%)	0 (0%)	0 (0%)	170 (100%)	0 (0%)	0 (0%)	0 (0%)
Other tool	122 (4.5%)	0 (0%)	0 (0%)	0 (0%)	0 (0%)	0 (0%)	122 (100%)	0 (0%)	0 (0%)
Treatment with a DOAC, *n* (%)	1549 (60%)	0 (0%)	3 (1%)	565 (83%)	604 (100%)	170 (100%)	43 (35%)	^i^	165 (86%)
Risk factors, *n* (%)									
Recent immobilization or surgery	261 (11%)	27 (9%)	69 (13%)	68 (10%)	54 (9%)	26 (15%)	17 (14%)	^i^	^i^
Oestrogen use	290 (12%)	47 (16%)	91 (18%)	64 (9%)	41 (7%)	31 (18%)	8 (7%)	8 (7%)	^i^
Symptomatic PE, *n* (%)	1645 (99%)	296 (100%)	513 (100%)	681 (100%)	^i^	155 (91%)	^i^	^i^	^i^
Vital signs at presentation^b^, *n* (%)									
Heart rate ≥ 110 b.p.m.	223 (9%)	27 (9%)	35 (7%)	63 (9%)	62 (10%)	3 (2%)	^i^	15 (13%)	18 (9%)
Respiratory rate of ≥30/min	13 (1%)	^i^	^i^	^i^	5 (1%)	2 (1%)	^i^	4 (3%)	2 (1%)
Oxygen saturation < 90% or need for oxygen	56 (2%)	19 (6%)	4 (1%)	23 (3%)	2 (0%)	1 (1%)	^i^	5 (4%)	0 (0%)
Comorbidities, *n* (%)									
Cancer^c^	227 (8%)	28 (9%)	34 (7%)	50 (7%)	24 (4%)	19 (11%)	55 (45%)	16 (14%)	2 (1%)
Previous VTE	842 (33%)	74 (25%)	120 (23%)	169 (25%)	375 (62%)	34 (20%)	34 (28%)	^i^	37 (19%)
Decreased kidney function^d^	207 (8%)	12 (4%)	22 (4%)	66 (10%)	67 (11%)	12 (7%)	11 (9%)	11 (9%)	6 (3%)
Pre-existing cardiopulmonary disease^e^	487 (18%)	12 (4%)	25 (5%)	161 (24%)	186 (31%)	12 (7%)	30 (25%)	28 (24%)	32 (17%)
Laboratory/imaging results, *n* (%)									
Abnormal troponin^f^	249 (11%)	^i^	67 (13%)	117 (17%)	53 (9%)	^i^	4 (3%)	9 (8%)	1 (1%)
Abnormal (NT-pro)BNP^g^	212 (9%)	^i^	27 (5%)	78 (11%)	80 (13%)	10 (6%)	11 (9%)	21 (18%)	6 (3%)
RV overload^h^	331 (26%)	201 (68%)	^i^	106 (16%)	^i^	9 (5%)	15 (12%)	^i^	^i^
RVD on echocardiography	31 (3%)	^i^	^i^	^i^	20 (3%)	0 (0%)	6 (5%)	3 (3%)	2 (1%)

DOAC, direct oral anticoagulant; PE, pulmonary embolism; RV, right ventricular; RVD, right ventricular dysfunction SD, standard deviation; VTE, venous thromboembolism.

^a^In combination with a negative clinical judgement.

^b^Vinson *et al.*^31^ reported the worst vital signs throughout the hole of the patients’ ED stay.

^c^Cancer was defined as (i) a current diagnosis of cancer, (ii) receiving treatment for cancer, or (iii) not receiving treatment for cancer and not in complete response.

^d^Estimated glomerular filtration rate < 60 mL/min.

^e^Pre-existing pulmonary disease was defined as a history of chronic obstructive pulmonary disease, asthma, or lung fibrosis; a pre-existing cardiovascular disease was defined as any of coronary artery disease, heart failure, congenital heart disease, cardiomyopathy, or rheumatic heart disease.

^f^Abnormal troponin was defined as a troponin level > 99th percentile according to local technique.

^g^(NT-pro)BNP > 500 ng/L or BNP level > 100 ng/L.

^h^Right ventricle/left ventricle ratio > 0.9 on computed tomography pulmonary angiogram or echocardiogram.

^i^Variable systematically missing within a study.

### Outcomes

#### All-cause mortality

*Table 4* presents the overall incidence of safety outcomes at 14 and 30 days in patients discharged within 24 h. At 14 days, three patients had died, corresponding to a pooled 14-day mortality of 0.11% (95% CI 0.0–0.24). One had a PE-related death, one had a major bleeding–related death, and one died due to a cause other than PE or major bleeding. The 14-day incidence of combined adverse events was 0.56% (95% CI 0.28–0.84), 0.34% (95% CI 0.12–0.56) for recurrent VTE, and 0.19% (95% CI 0.03–0.35) for major bleeding.

**Table 4:** Overall incidence of safety outcomes

	14 days	30 days
**All-cause mortality, %, (95%CI)**		
All patients discharged within 24 h	0.11 (0.0–0.24)	0.30 (0.09–0.51)
Including Font *et al.*^27^	0.18 (0.02–0.34)	0.37 (0.14–0.60)
Triage tool: Hestia (or Hestia-like) rule	0.19 (0.0–0.40)	0.31 (0.04–0.58)
Triage tool: PESI or sPESI^a^	0.0 (0.0–0.0)	0.21 (0–0.63)
All patients discharged within 120 h	0.25 (0.08–0.42)	0.40 (0.18–0.62)
**Recurrent VTE, % (95% CI)**		
All patients discharged within 24 h	0.34 (0.12–0.56)	0.57 (0.28–0.86)
Including Font *et al.*^27^	0.37 (0.14–0.60)	0.59 (0.30– 0.88)
Triage tool: Hestia (or Hestia-like) rule	0.52 (0.17–0.87)	0.80 (0.36–1.2)
Triage tool: PESI or sPESI^a^	0.11 (0.0–0.41)	0.43 (0.0–1.0)
All patients discharged within 120 h	0.43 (0.20–0.66)	0.65 (0.37–0.93)
**Major bleeding, %, (95% CI)**		
All patients discharged within 24 h	0.19 (0.03–0.35)	0.45 (0.19–0.71)
Including Font *et al.*^27^	0.22 (0.04–0.40)	0.52 (0.25–0.79)
Triage tool: Hestia (or Hestia-like) rule	0.35 (0.06–0.64)	0.62 (0.24–1.0)
Triage tool: PESI or sPESI^a^	0.0 (0.0–0.0)	0.43 (0.0–1.0)
All patients discharged within 120 h	0.28 (0.10– 0.46)	0.53 (0.28–0.78)
**Combined endpoint, % (95% CI)**		
All patients discharged within 24 h	0.56 (0.28–0.84)	1.2 (0.79–1.6)
Including Font *et al.*^27^	0.66 (0.36–0.96)	1.3 (0.90– 1.8)
Triage tool: Hestia (or Hestia-like) rule	0.86 (0.41–1.31)	1.5 (0.94–2.1)
Triage tool: PESI or sPESI^a^	0.21 (0.0–0.63)	1.1 (0.13–2.0)
All patients discharged within 120 h	0.77 (0.47–1.1)	1.4 (0.96–1.8)

PESI, pulmonary embolism severity index; sPESI, simplified pulmonary embolism severity index.

^a^In combination with a negative clinical judgement.

At 30 days, eight patients had died, corresponding to a pooled 30-day mortality of 0.30% (95% CI 0.09–0.51). Two out of eight had a PE-related death, one had a major bleeding–related death, and five died due to a cause other than PE or major bleeding. The 30-day incidence of all adverse events was 1.2% (95% CI 0.79–1.6), 0.57% (95% CI 0.28–0.86) for recurrent VTE, and 0.45% (95% CI 0.19–171) for major bleeding.

**Figure 2:**
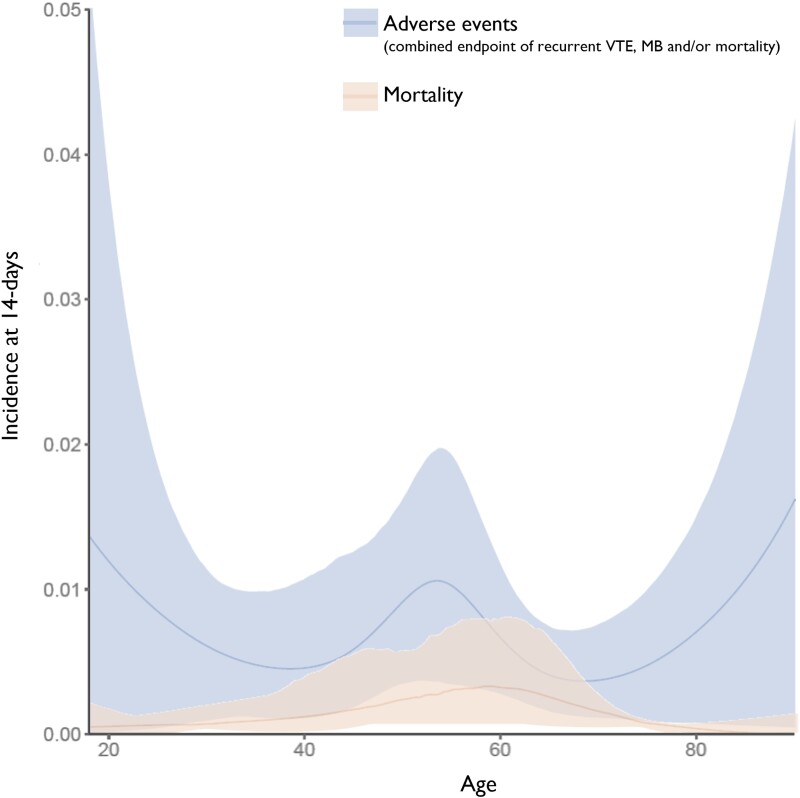
Incidence (%) of 14-day adverse events and mortality with 95% prediction intervals vs. age (in years) as a continuous variable. For distribution of age, see Supplementary data online, *Appendix D*; *Figure S2*. MB, major bleeding; VTE, venous thromboembolism

Age and sex were not associated with an increased 14- or 30-day mortality (*Figure 2* and *Tables 5* and *6*; Supplementary data online, *Appendix D*; *Figure S1*). In terms of cardiopulmonary comorbidities and signs of RV dysfunction (i.e. RV/LV ratio > 0.9, elevated cardiac biomarkers), no subgroup was associated with an increased 14- or 30-day mortality. Only patients with cancer had an increased 30-day mortality (RR 4.9; 95% PI 2.7–9.1; *Table 6*).

**Table 5:** Combined endpoint and mortality at 14 days of all patients that were discharged within 24 h

	Combined endpoint of VTE, MB, or all-cause morality						All-cause mortality					
	Events (*n*)	Patients (*n*)	%	(95% CI)	RR	(95% PI)	Events (*n*)	Patients (*n*)	%	(95% CI)	RR	(95% PI)
Overall	15	2660	0.56	(0.28–0.84)			3	2664	0.11	(0.0–0.24)		
Age												
18–40*	3	580	0.52	(0.0–1.1)			0	582	0.00	(0.0–0)		
41–60	8	1086	0.74	(0.23–1.3)	1.10	(0.66–1.8)	2	1086	0.18	(0.0–0.44)	1.28	(0.9–1.82)
61–80	4	894	0.45	(0.01–0.8)	0.96	(0.48–1.4)	1	896	0.11	(0.0–0.33)	1.14	(0.86–1.5)
>81	0	99	0.00	(0.0–0)	0.77	(0.52–1.2)	0	99	0.00	(0.0–0)	NA	NA
Sex												
Female	6	1264	0.47	(0.09–0.85)	1.1	(0.48–2.4)	2	1266	0.16	(0.0–0.38)	1.3	(0.65–2.5)
Male*	9	1396	0.64	(0.22–1.1)			1	1398	0.07	(0.0–0.21)		
Symptoms												
Incidental	0	15	0.00	(0.0–0)	1.0	(0.0–1005)	0	15	0.00	(0.0–0)	1.0	(0.0–1005)
Symptomatic*	10	1641	0.61	(0.23–0.99)			3	1641	0.18	(0.0–0.39)		
Treatment												
LMWH or VKA	7	1012	0.69	(0.18–1.2)	1.3	(0.78–2.3)	3	1014	0.30	(0.0–0.63)	3.1	(0.17–56)
DOAC*	8	1532	0.52	(0.16–0.88)			0	1534	0.00	(0.0–0)		
Cancer^a^												
Yes	1	217	0.46	(0.0–1.4)	1.7	(0.7–3.9)	1	219	0.46	(0.0–1.4)	2.9	(0.8–10)
No*	14	2443	0.57	(0.27–0.87)			2	2445	0.08	(0.0–0.19)		
Previous VTE												
Yes	7	830	0.81	(0.2–1.4)	1.3	(0.55–3.3)	1	831	0.12	(0.0–0.36)	1.3	(0.49–3.4)
No*	8	1714	0.48	(0.15–0.81)			2	1717	0.12	(0.0–0.28)		
Decreased kidney function^b^												
Yes	1	203	0.25	(0.0–0.94)	0.47	(0.22–1.0)	0	203	0.00	(0.0–0)	0.78	(0.58–1.1)
No*	14	2457	0.59	(0.29–0.89)			3	2461	0.12	(0.0–0.26)		
Pre-existing cardiopulmonary disease^c^												
Yes	6	479	1.30	(0.29–2.3)	3.5	(1.5–7.9)	1	480	0.28	(0.0–0.75)	2. 70^g^	(0.68–11)
No*	9	2181	0.40	(0.13–0.67)			2	2184	0.08	(0.0–0.2)		
Abnormal troponin^d^												
Yes	3	249	1.23	(0.0–2.6)	2.5	(1.3–4.9)	0	249	0.14	(0.0–0.6)	0.86	(0.56–1.3)
No*	8	1946	0.41	(0.13–0.69)			2	1950	0.08	(0.0–0.21)		
Abnormal (NT-pro)BNP^e^												
Yes	3	210	1.60	(0.0–3.3)	3.9	(1.6–9.8)	0	210	0.10	(0.0–0.53)	0.93	(0.75–1.1)
No*	8	2154	0.35	(0.1–0.6)			2	2158	0.08	(0.0–0.2)		
Signs of RV overload^f^												
Yes	5	326	1.69	(0.29–3.1)	2.7	(0.62–11)	0	327	0.15	(0.0–0.57)	0.49	(0.1–2.4)
No*	3	910	0.28	(0.0–0.62)			2	913	0.17	(0.0–0.43)		

This table presents the 14-day incidence of the combined endpoint of VTE, MB, or all-cause mortality and the 14-day incidence of all-cause mortality. Relative risk presents the ratio of the risk for an event for the exposure group to the risk for the non-exposure/reference group. Non-exposure/reference group is marked with an asterisk.

CI, confidence interval; DOAC, direct oral anticoagulant; LMWH, low-molecular-weight heparin; MB, major bleeding; NA, not applicable; PI, prediction interval; RR, relative risk; RV, right ventricle; TTE, transthoracic echocardiography; VKA, vitamin K antagonist; VTE, venous thromboembolism.

^a^(i) Current diagnosis of cancer, (ii) receiving treatment for cancer, or (iii) not receiving treatment for cancer and not in complete response.

^b^Estimated glomerular filtration rate < 60 mL/min.

^c^Pre-existing pulmonary disease was defined as a history of chronic obstructive pulmonary disease, asthma, or lung fibrosis; a pre-existing cardiovascular disease was defined as any of coronary artery disease, heart failure, congenital heart disease, cardiomyopathy, or rheumatic heart disease.

^d^Abnormal troponin was defined as a troponin level > 99th percentile according to local technique.

^e^(NT-pro)BNP > 500 ng/L or BNP level > 100 ng/L.

^f^Right ventricle/left ventricle ratio > 0.9 on computed tomography pulmonary angiogram or echocardiogram.

^g^ *I* ^2^ were all 0% for all analysis, except for *I*^2^ = 0.68%.

**Table 6:** Combined endpoint and mortality at 30 days of all patients that were discharged within 24 h

	Combined endpoint of VTE, MB, or all-cause morality						All-cause mortality					
	Events (*n*)	Patients (*n*)	%	(95% CI)	RR	(95% PI)	Events (*n*)	Patients (*n*)	%	(95% CI)	RR	(95% PI)
Overall	32	2653	1.2	(0.79–1.6)			8	2660	0.30	(0.09–0.51)		
Age												
18–40*	8	580	1.4	(0.43–2.3)			1	582	0.17	(0.0–0.51)		
41–60	12	1084	1.1	(0.49–1.7)	0.82	(0.45–1.5)	2	1085	0.18	(0.0–0.44)	0.93	(0.47–1.8)
61–80	12	889	1.4	(0.59–2.1)	0.91	(0.61–1.4)	5	893	0.56	(0.07–1.1)	1.3	(0.54–2.9)
>81	0	99	0.0	(0.0–0.0)	0.49	(0.31–0.77)	0	99	0.0	(0.0–0.0)	0.84	(0.62–1.1)
Sex												
Female	18	1260	1.4	(0.77–2.1)	1.4^g^	(0.57–3.4)	6	1264	0.47	(0.09–0.85)	1.7	(0.98–2.9)
Male*	14	1393	1.0	(0.49–1.5)			2	1396	0.14	(0.0–0.34)		
Symptoms												
Incidental	0	15	0.0	(0.0–0)	1	(0.0–986)	0	15	0.0	(0.0–0)	1.0	(0.0–986)
Symptomatic*	20	1640	1.2	(0.69–1.8)			6	1640	0.37	(0.08–0.66)		
Treatment												
LMWH or VKA	14	1007	1.4	(0.67–2.1)	1.4	(0.72–2.9)	6	1011	0.59	(0.12–1.1)	2.6	(0.91–7.5)
DOAC*	17	1530	1.1	(0.58–1.6)			1	1533	0.07	(0.0–0.2)		
Cancer^a^												
Yes	5	211	2.4	(0.31–4.4)	2.7	(1.4–5.2)	4	215	1.9	(0.06–3.7)	4.9	(2.7–9.1)
No*	27	2442	1.1	(0.7–1.5)			4	2445	0.16	(0.0–0.32)		
Previous VTE												
Yes	13	829	1.6	(0.73–2.4)	1.3	(0.65–2.6)	3	831	0.36	(0.0–0.77)	1.8	(0.57–5.7)
No*	18	1708	1.1	(0.57–1.5)			4	1713	0.23	(0.0–0.46)		
Decreased kidney function^b^												
Yes	1	202	0.49	(0.0–1.5)	0.35	(0.14–0.88)	0	203	0.18	(0.0–0.76)	0.66	(0.39–1.1)
No*	31	2451	1.3	(0.82–1.7)			8	2457	0.31	(0.09–0.53)		
Pre-existing cardiopulmonary disease^c^												
Yes	8	476	1.8	(0.57–2.9)	1.9	(0.9–3.8)	2	478	0.34	(0.0–0.86)	1.8^i^	(0.36–9.5)
No*	24	2177	1.1	(0.65–1.5)			6	2182	0.29	(0.06–0.52)		
Abnormal troponin^d^												
Yes	6	248	2.6	(0.59–4.5)	2.9	(1.5–5.7)	1	249	0.6	(0.0–1.6)	2.2	(0.59–8.1)
No*	19	1941	1.0	(0.53–1.4)			5	1947	0.23	(0.02–0.44)		
Abnormal (NT-pro)BNP^e^												
Yes	6	208	2.7	(0.47–4.9)	3.3	(1.6–7.1)	1	210	0.4	(0.0–1.3)	0.84	(0.52–1.4)
No*	19	2149	0.91	(0.51–1.3)			5	2154	0.24	(0.03–0.45)		
Signs of RV overload^f^												
Yes	9	325	2.7	(0.96–4.5)	2.0^h^	(0.68–6)	2	327	0.55	(0.0–1.4)	0.70	(0.4–1.2)
No*	8	905	0.9	(0.29–1.5)			3	909	0.35	(0.0–0.74)		

This table presents the 30-day incidence of the combined endpoint of VTE, MB, or all-cause mortality and the 30-day incidence of all-cause mortality. Relative risk presents the ratio of the risk for an event for the exposure group to the risk for the non-exposure/reference group. Non-exposure/reference group is marked with an asterisk.

CI, confidence interval; DOAC, direct oral anticoagulant; LMWH, low-molecular-weight heparin; MB, major bleeding; PI, prediction interval; RR, relative risk; RV, right ventricle; TTE, transthoracic echocardiography; VKA, vitamin K antagonist; VTE, venous thromboembolism.

^a^(i) Current diagnosis of cancer, (ii) receiving treatment for cancer, or (iii) not receiving treatment for cancer and not in complete response.

^b^Estimated glomerular filtration rate < 60 mL/min.

^c^Pre-existing pulmonary disease was defined as a history of chronic obstructive pulmonary disease, asthma, or lung fibrosis; a pre-existing cardiovascular disease was defined as any of coronary artery disease, heart failure, congenital heart disease, cardiomyopathy, or rheumatic heart disease.

^d^Abnormal troponin was defined as a troponin level > 99th percentile according to local technique.

^e^(NT-pro)BNP > 500 ng/L or BNP level > 100 ng/L.

^f^Right ventricle/left ventricle ratio > 0.9 on computed tomography pulmonary angiogram or echocardiogram.

^g^ *I* ^2^ were all 0% for all analysis, except for *I*^2^ = 7.3%.

^h^ *I* ^2^ were all 0% for all analysis, except for *I*^2^ = 0.32%.

^i^ *I* ^2^ were all 0% for all analysis, except for *I*^2^ = 4.2%.

#### Adverse events (combined endpoint of all-cause mortality, recurrent venous thromboembolism, and major bleeding)

Pre-existing cardiopulmonary comorbidity, an abnormal troponin, and an abnormal (NT-pro)BNP were all associated with an increased incidence of 14-day adverse events [RR 3.5 (95% PI 1.5–7.9), 2.5 (95% PI 1.3–4.9), and 3.9 (95% PI 1.6–9.8), respectively; *Table 5*]. At 30 days, an abnormal troponin, an abnormal (NT-pro)BNP, and cancer were associated with an increased incidence of adverse events [RR 2.9 (95% PI 1.5–5.7), 3.3 (95% PI 1.6–7.1), and 2.7 (95% PI 1.4–5.2), respectively; *Table 6*].

Decreased kidney function was associated with a lower risk of 14- and 30-day adverse events [0.47 (95% PI 0.22–1.0) and 0.35 (95% PI 0.14–0.88), respectively; *Tables 5* and *6*].

Subgroup analysis for recurrent VTE and major bleeding are presented in Supplementarydata online, *Appendix D*; *Tables S3* and *S4*.

#### Hestia or simplified pulmonary embolism severity index

There was no clear difference in all-cause mortality between patients selected by Hestia or (s)PESI plus clinical judgement (*Table 4*). Patients selected using Hestia had a higher incidence of recurrent VTE than patients selected using (s)PESI [14 days, 0.52% (95% CI 0.17–0.87) vs. 0.11% (95% CI 0.0–0.41); 30 days, 0.80% (95% CI 0.36–1.2) vs. 0.43% (95% CI 0.0–1.0), respectively] and a higher incidence of major bleeding [14 days, 0.35% (95% CI 0.06–0.64) vs. 0.0% (95% CI 0.00–0.0); 30 days, 0.62% (95% CI 0.24–1.0) vs. 0.43% (95% CI 0.0–1.0), respectively].

#### Sensitivity analysis

According to the definition of home treatment from the original studies (discharge within 120 h at most), 3301 patients received home treatment. Of these patients, 83% were discharged <24 h, 12% within 24–48 h, 1.4% within 48–72 h, and 0.9% within 72–120 h, and in 2%, information on time to discharge was unknown. The baseline characteristics of all 3301 patients are demonstrated in Supplementary data online, *Appendix D*; *Table S5*. All sensitivity analyses, including those based on the definition of home treatment in the original studies (see Supplementary data online, *Appendix D*; *Tables S6–S9*), the inclusion of Font *et al.*^27^ (see Supplementary data online, *Appendix D*; *Tables S10–S14*), and the analysis based on the non-imputed data (see Supplementary data online, *Appendix D*; *Tables S15–S18*), revealed no substantial differences in the incidence of adverse outcomes or subgroup analyses compared with the main analysis.

## Discussion

In this IPDMA, home-treated PE patients, who were selected using predefined validated triage tools [e.g. Hestia rule or (s)PESI in combination with a negative clinical judgement], had low 14-day mortality (0.11%) and incidence of adverse events (0.56%). As expected, patients with cancer showed a higher (three- to five-fold) all-cause mortality and incidence of adverse events. Patients with increased troponin or (NT-pro)BNP had an approximately three-fold higher incidence of adverse events, but not of mortality (Structured Graphical Abstract).

The ESC guideline risk stratification model suggests that the sPESI score or Hestia rule should be used to select patients eligible for home treatment.^2^ By default, according to sPESI, all patients with cancer, with chronic cardiopulmonary disease, or older than 80 years should be hospitalized.^7^ In line with previous studies and this recommendation, our study confirmed a higher incidence of death and adverse events in cancer patients treated at home.^7,13^ However, the absolute risk was low, and mortality was partially due to the underlying cancer. Out of the six patients with cancer that died within 30 days, only one patient had a PE-related death after 10 days and one patient died of major bleeding after 5 days. Notably, we found no increased mortality in patients older than 80 years who were selected for home treatment. Patients with pre-existing cardiopulmonary comorbidity had a higher incidence of adverse events at 14 days but not at 30 days, which was mainly driven by a higher incidence of recurrent VTE as there was no higher incidence of mortality.

According to ESC guidelines, PE patients with RV overload on CTPA or with increased troponin levels require hospitalization.^2^ Elevation of other laboratory biomarkers, such as (NT-pro)BNP, may provide additional prognostic information.^2,32^ This recommendation is based on a meta-analysis that showed that otherwise ‘low-risk’ patients (i.e. sPESI of 0 or negative Hestia rule) with RV overload, abnormal troponin, or abnormal (NT-pro)BNP have an increased risk of 30-day mortality (RR 3.37, 5.14, and 3.63, respectively).^12^ The current study did not show an association between 30-day mortality and RV overload or abnormal biomarkers. The observed difference between the two studies is likely due to the inclusion of hospitalized patients in the other meta-analysis, while the current meta-analysis focused on patients selected for home treatment by fulfilling low-risk criteria based on the individual triage tools. On the other hand, RV overload represented a formal exclusion criterion in some trials, whereas it was part of the broader clinical judgement in most of the other trials adopting either the sPESI or Hestia rule, possibly resulting in an underestimation of the association. Clinical judgement on top of triage tools nonetheless seems to add additional safety in selecting low-risk patients eligible for home treatment, partly diluting the additional value of cardiac markers or RV overload.^14,33^ Echocardiographic-assessed RV dysfunction had the highest proportion of missing data across the included studies, was found in a low number of patients, and its definition was not homogeneous across studies. We could therefore not provide a solid conclusion on the safety of home treatment in patients with RV dysfunction on echocardiography and decided to show only this data in Supplementary data online, *Appendix D*; *Tables S19* and *S20*.

Patients with renal impairment appeared to have a better outcome of care than those with normal renal function. This seems contradictory and could be explained by (i) the exclusion of patients with severe renal impairment (estimated glomerular filtration rate < 30 mL/min) from most studies and (ii) the low number of patients in this category in our database. Our interpretation is that patients with mild-to-moderate renal insufficiency who do not meet any of the Hestia criteria or are considered at low risk of death by the sPESI at least do not face a clearly higher incidence of adverse outcomes.

The interpretation of absolute risks is clinically more relevant than that of RRs in patients with (vs. without) a subgroup variable. When considering the safety of home treatment of acute PE, it can be debated what absolute threshold for early mortality rate is acceptable. In the original sPESI study, a 30-day all-cause mortality of 1.1% among patients is identified as low-risk.^7^ Adding additional criteria to sPESI or Hestia for assessing home treatment eligibility would most likely result in a lower risk of mortality, although at the cost of a lower number of patients eligible for home treatment, as was shown in the HoT-PE trial.^25^ Patients with signs of cardiopulmonary impairment, including those with elevated troponin or (NT-pro)BNP and/or signs of RV dysfunction or RV overload, had an absolute 30-day risk of adverse events exceeding 2.5%, although 30-day mortality was only 0.40%–0.60%. These absolute risks should inform clinicians and patients concerning the safety of early discharge and home treatment. From a healthcare resource perspective, if all deaths in our study were considered to be PE-related and preventable by hospitalization, 58–263 additional acute PE patients with cancer or 500 additional acute PE patients with RV overload would need to be hospitalized to prevent one death. Clearly, it remains questionable whether hospitalization would have actually prevented these deaths, in particular in the case of cancer-related death, or other complications as recurrent VTE or bleeding as there is no comparison between hospitalized and home-treated patients. Therefore, when looking at preventing PE-related complications in our study, the added value of hospitalization remains debatable. As hospitalization is more expensive than home treatment, healthcare costs associated with hospitalizations must also be considered.^4^

When considering eligibility for home treatment, clinical judgement and individualized treatment decisions remain important. This was highlighted by the HOME-PE trial: after a shared decision-making, 0.5%–3.3% of the patients deemed ineligible for home treatment by the Hestia rule or sPESI ultimately received home treatment, and 3.4% (by the Hestia rule) and 28.5% (by the sPESI) of the patients deemed eligible for home treatment were ultimately hospitalized.^11^ Studies within this IPDMA that utilized the (s)PESI score for home treatment eligibility also incorporated clinical judgement. Only patients with a PESI II/III or sPESI of 0 in combination with a negative clinical judgement actually receive home treatment. Therefore, the application of risk classification scores used in this IPDMA in daily practice should always be combined with a clinical judgement. Clinical judgement is not only important for overruling home treatment, but hospitalization might also be overruled in certain patients based on clinical judgement and individualized decision-making. For patients with a limited life expectancy, such as patients with cancer, focusing on other outcomes such as patient satisfaction or quality of life might be more important than the risk of death. Home treatment has been associated with high patient satisfaction, although this has only been investigated by two studies, without a comparison with comparable hospitalized patients.^3,34^

The feasibility of home treatment has increased in recent years with the introduction of direct oral anticoagulants (DOACs), as these are safer and easier to use than conventional treatment. Up to 40% of the patients included in this IPDMA were treated with a vitamin K antagonist, which has been associated with a higher bleeding risk compared with DOACs.^35^ This was also confirmed in our study, where patients treated with a vitamin K antagonist had an incidence of major bleeding at 14 days of 0.30% compared with 0.13% for those treated with a DOAC. Ultimately, implementation of home treatment strategies, including specific selection criteria, depends on local healthcare systems and infrastructure and therefore may vary across different geographical, social, and cultural contexts.

Our study has strengths and limitations. Its main strength lies in its large number of patients and the state-of-the-art statistical methods. This enabled evaluation of the safety of home treatment with more accuracy and narrower 95% CIs than reported previously. This is also the first study to investigate specific subgroups of interest suspected to be at higher risk for adverse events when receiving home treatment.

As a first limitation, the calculation of RRs is difficult within subgroups with few events, resulting in RRs with a higher level of uncertainty, reflected in broad 95% PIs. Even so, we used Firth’s correction to handle small-sample bias. Some subgroups may exhibit a non-significant RR for adverse outcomes due to a lack of statistical power. However, this is because overall absolute risks in these subgroups were low. Therefore, the emphasis should be on considering absolute risks rather than solely detecting differences in risks, especially when comparing incidence rates that potentially fall within a range considered safe from a clinical perspective. Second, we have performed multiple imputations of variables with a high level of missingness. For data sets where variables are missing (completely) at random, this approach is reliable and will reduce bias.^36^ We assumed that missing (completely) at random was mostly applicable for our data set. However, we did not have exact information on how each variable was collected in a data set, so we cannot guarantee that missing values were truly missing at random, as abnormal values might have been more frequently reported than normal ones. Imputed values may, therefore, not accurately reflect true (unobserved) values. We have reported all percentages of missingness in Supplementary data online, *Appendix D*; *Table S1*, aiming for transparency when interpreting the data. Third, the subgroup definitions applied in this IPDMA were not fully standardized. Fourth, our data include only adverse event rates but do not contain other relevant outcomes such as unscheduled visits, patient satisfaction, quality of life, or cost-effectiveness. Such outcomes therefore were not included in this IPDMA, nor were data of patients that were hospitalized for comparison. Finally, some studies included in our IPDMA excluded patients with certain subgroup characteristics (e.g. cancer, RV overload), which may have resulted in an underestimation of the prognostic impact of these characteristics in our analysis. The current study did not show an association between troponin, (NT-pro)BNP, RV overload, and mortality in patients selected for home treatment, but this association might have been underestimated due to this limitation, and these findings should thus be interpreted with caution. Clinicians should focus on the absolute incidences, while keeping in mind the uncertainty due to the small number with the reflecting 95% CI, when discussing the risk of home treatment and assessing home treatment as a potential treatment option.

## Conclusions

Validated triage tools such as Hestia or sPESI in combination with a negative clinical judgement can be used in the emergency department to select acute PE patients for home treatment, as the rate of adverse events and death in our cohort was very low. Patients with cancer had a three- to five-fold higher incidence of 30-day mortality or adverse events. Patients with increased troponin or (NT-pro)BNP had a three-fold higher risk of adverse events, driven by recurrent VTE and bleeding complications. The point estimates of the absolute risk of adverse events provide important evidence to inform clinical shared decision-making in daily practice.

## Supplementary Material

ehae378_Supplementary_Data

## Declarations

### Data Availability

Data from individual studies were provided by the authors for the specific purpose of conducting this IPDMA. Any requests by other researchers for those data should be directed to the responsible party for individual studies.

### Pre-registered Clinical Trial Number

The pre-registered clinical trial number is PROSPERO CRD42021272504.
