# Supplementary material for: Safety of treating acute pulmonary embolism at home: an individual patient data meta-analysis
Source: Eur Heart J. 2024 Jul 12;45(32):2933–50. doi: 10.1093/eurheartj/ehae378 (PMC11335374; doi:10.1093/eurheartj/ehae378)
Supplement: ehae378_Supplementary_Data [file ehae378_supplementary_data.pdf]

Supplementary file*: S*afety of home treatment of acute pulmonary embolism in the overall population and clinically relevant patient subgroups: A systematic review and individual-patient data meta-analysis

Dieuwke Luijten, Delphine Douillet , Kim Luijken, Cecile Tromeur , Andrea Penaloza, Olivier Hugli, Drahomir Aujesky, Stefano Barco, Joseph R. Bledsoe, Kyle E. Chang, Francis Couturaud, Paul L. den Exter, Carme Font, Menno V. Huisman, David Jimenez, Christopher Kabrhel, Jeffrey A Kline, Stavros Konstantinides, Thijs van Mens, Remedios Otero, W. Frank Peacock, Olivier Sanchez, William B. Stubblefield, Luca Valerio, David R Vinson, Philip Wells, Maarten van Smeden, Pierre-Marie Roy and Frederikus A. Klok

Department of Medicine – Thrombosis and Hemostasis, Leiden University Medical Center, Leiden, the Netherlands (D Luijten MD, PL den Exter MD PhD, prof MV Huisman MD PhD, T van Mens MD PhD, prof FA Klok MD PhD), **Emergency Department, CHU Angers, Angers, France** (D Douillet MD PhD, prof PMR Roy MD PhD)**, University of Angers, UMR MITOVASC INSERM 1083 -CNRS 6015, Equipe CARME, SFR ICAT, Angers, France** (D Douillet MD PhD, prof PM Roy MD PhD)**, F-CRIN, INNOVTE, Saint-Etienne, France** (D Douillet MD PhD, prof C Tromeur MD, PhD, Andrea Penaloza MD PhD, prof F Couturaud MD PhD, prof O Sanchez MD, PhD, prof PMR Roy MD PhD)**, Department of Epidemiology, Julius Center for Health Sciences and Primary Care, University Medical Center Utrecht, Utrecht University, Utrecht, the Netherlands** (K Luijken PhD, prof M van Smeden PhD)**, Université de Bretagne Occidentale, INSERM U1304-GETBO, Département de Médecine Interne et Pneumologie, Centre Hospitalo-Universitaire de Brest, F 29200, Brest, France** (prof C Tromeur MD PhD, prof F Couturaud MD PhD)**, Emergency Department, Cliniques Universitaires Saint-Luc, Brussels, Belgium** (Andrea Penaloza MD PhD)**, UCLouvain, Brussels, Belgium** (Andrea Penaloza MD PhD)**, Emergency Department, University Hospital of Lausanne and University of Lausanne, Lausanne, Switzerland** (O Hugli MD)**, Department of General Internal Medicine, Inselspital, Bern University Hospital, University of Bern, Bern, Switzerland** (D Aujesky MD MSc)**, Center for Thrombosis and Hemostasis, University Medical Center of the Johannes Gutenberg University, Mainz, Germany** (S Barco MD PhD, prof S Konstantinides MD PhD, L Valerio MD PhD, prof FA Klok MD PhD)**, Department of Angiology, University Hospital Zurich, Zurich, Switzerland** (S Barco MD PhD)**, Department of Emergency Medicine Intermountain Healthcare, Salt Lake City, Utah, USA** (JR Bledsoe MD)**, Center for Vascular Emergencies, Department of Emergency Medicine, Massachusetts General Hospital, Harvard Medical School, Boston, Massachusetts, USA** (KE Chang BSc, C Kabrhel MD MPH)**, School of Medicine, California University of Science and Medicine, Colton, California, USA** (KE Chang BSc)**, Medical Oncology Department, Hospital Clinic de Barcelona, Barcelona, Spain** (C Font MD PhD)**, Respiratory Department and Medicine Department, Ramon y Cajal Hospital and Alcalá University (IRYCIS); CIBER de Enfermedades Respiratorias (CIBERES), Madrid, Spain** (D Jimenez MD PhD)**, Wayne State University School of Medicine, Detroit, MI, USA** (J Kline MD)**, Department of Cardiology, Democritus University of Thrace, Alexandroupolis, Greece** (prof S Konstantinides MD PhD)**, Pneumology Department, Hospital Universitario Virgen del Rocío-IBIS-US_CIBERES, Seville, Spain** (R Otero MD PhD), **Department of Emergency Medicine, Baylor College of Medicine, Ben Taub General Hospital, Houston, Texas, USA (**WF Peacock MD), **University Paris Cité, INSERM UMR-S 1140 Innovaties Therapies in Haemostasis, Paris, France** (prof O Sanchez MD, PhD), **Pneumology Department and Intensive Care, Hôpital Européen Georges Pompidou, APHP, 20-40 rue Leblanc, Paris, France** (prof O Sanchez MD, PhD)**, Department of Emergency Medicine, Vanderbilt University Medical Center, Nashville, Tennessee, USA** (WB Stubblefield MD, MPH), **Department of Cardiology, University Medical Center of the Johannes Gutenberg University, Mainz, Germany** (L Valerio MD PhD)**, The Permanente Medical Group, Oakland, California, USA** (D Vinson MD), **Kaiser Permanente Division of Research, Oakland, California, USA** (D Vinson MD), **The Kaiser Permanente CREST Network, USA** (D Vinson MD)**, Department of Emergency Medicine, Kaiser Permanente Roseville Medical Center, Roseville, California, USA** (D Vinson MD)**, Department of Medicine, University of Ottawa, Ottawa, ON, Canada** (prof P Wells MD PhD)

Contents

Appendix A: search strategy ... 3

Appendix B: variables IPDMA home treatment ... 6

Appendix C: definitions of subgroups ... 13

Appendix D: supplementary tables and figures ... 14

Table S1: description of missing data ... 14

Table S2: risk of bias assessment using the adapted NOS (Newcastle-Ottawa Scale) ... 24

Table S3: overall and subgroup analysis at 14- and 30-days for recurrent VTE incidence ... 25

Table S4: overall and subgroup analysis at 14- and 30-days for major bleeding ... 26

Sensitivity analysis: according to the definition of home-treatment of the original studies ... 27

Table S5: characteristics of patients that received home treatment according to the original study definitions ... 27

Table S6: overall and subgroup analysis at 14- and 30-days for mortality: sensitivity analysis according to the definition of home-treatment of the original studies 28

Table S7: overall and subgroup analysis at 14- and 30-days for all adverse events (i.e. combined endpoint of recurrent VTE, MB or mortality): sensitivity analysis according to the definition of home-treatment of the original studies ... 29

Table S8: overall and subgroup analysis at 14- and 30-days for recurrent VTE incidence: sensitivity analysis according to the definition of home-treatment of the original studies ... 30

Table S9: overall and subgroup analysis at 14- and 30-days for major bleeding: sensitivity analysis according to the definition of home-treatment of the original studies ... 31

Sensitivity analysis: Font et al included (discharge within 24 hours) ... 32

Table S10: characteristics patients that received home treatment (defined as discharge within 24 hours) including Font et al ... 32

Table S11: overall and subgroup analysis at 14- and 30-days for mortality: sensitivity analysis with Font et al included ... 33

Table S12: overall and subgroup analysis at 14- and 30-days for all adverse events (i.e. combined endpoint of recurrent VTE, MB or mortality): sensitivity analysis with Font et al included ... 34

Table S13: overall and subgroup analysis at 14- and 30-days for recurrent VTE: sensitivity analysis with Font et al included ... 35

Table S14: overall and subgroup analysis at 14- and 30-days for major bleeding: sensitivity analysis with Font et al included ... 36

Sensitivity analysis according to the non-imputed data (discharge within 24 hours) ... 37

Table S15 : overall and subgroup analysis at 14- and 30-days for mortality: sensitivity analysis non-imputed data (discharge within 24 hours) ... 37

Table S16: overall and subgroup analysis at 14- and 30-days for combined endpoint(i.e. combined endpoint of recurrent VTE, MB or mortality) : sensitivity analysis non-imputed data (discharge within 24 hours) ... 38

Table S17: overall and subgroup analysis at 14- and 30-days for recurrent VTE: sensitivity analysis non-imputed data (discharge within 24 hours) ... 39

Table S18: overall and subgroup analysis at 14- and 30-days for major bleeding: sensitivity analysis non-imputed data (discharge within 24 hours) ... 40

Echocardiography data main analysis ... 41

Table S19: echocardiography analysis at 14-days for mortality and all adverse events (i.e. combined endpoint of recurrent VTE, MB or mortality) ... 41

Table S20: echocardiography analysis at 30-days for mortality and all adverse events (i.e. combined endpoint of recurrent VTE, MB or mortality) ... 41

Figure S1: incidence of 30-day adverse events and mortality with 95% PIs versus age as a continuous variable ... 42

Figure S2: distribution of age in our cohort ... 43

Appendix E: Missing data imputation ... 44

References ... 45

Appendix A: search strategy

MEDLINE

(("Pulmonary Embolism"[mesh] OR "pulmonary embolism"[tw] OR "pulmonary embolisms"[tw] OR "pulmonary thromboembolisms"[tw] OR "pulmonary thromboembolism"[tw] OR "pulmonary thrombo embolism"[tw] OR "pulmonary thromboembol*"[tw] OR "pulmonary thrombo embol*"[tw]) AND ("home treatment"[tw] OR "home treat*"[tw] OR "hometreatment"[tw] OR "hometreat*"[tw] OR "treatment at home"[tw] OR "Home Care Services"[mesh] OR "home care"[tw] OR "home therapy"[tw] OR "home therap*"[tw] OR "outpatient treatment"[tw] OR "outpatient therapy"[tw] OR "outpatient care"[tw] OR "outpatient health service"[tw] OR "outpatient health services"[tw] OR "Outpatients"[mesh] OR "outpatient"[tw] OR "outpatients"[tw] OR "out of hospital treatment"[tw] OR "out of hospital"[tw] OR "outofhospital"[tw] OR "Ambulatory Care"[mesh] OR "ambulant treatment"[tw] OR "ambulatory treatment"[tw] OR "ambulatory therapy"[tw] OR "ambulatory care"[tw] OR "early discharge"[tw] OR "early discharges"[tw] OR "early discharged"[tw] OR "discharged early"[tw]) NOT (("Case Reports"[ptyp] OR "case report"[ti] OR "Review"[ptyp] OR "review"[ti]) NOT ("Clinical Study"[ptyp] OR "trial"[ti] OR "RCT"[ti] OR "systematic"[sb] OR "systematic review"[ti])))

Archivalia:

AND ("Safety"[mesh] OR "safety"[tw] OR "safe"[tw] OR "unsafe"[tw] OR "Risk"[mesh] OR "Risk Factors"[mesh] OR "Risk Assessment"[mesh] OR "risk"[tw] OR "risks"[tw] OR "Patient Readmission"[Mesh] OR "Readmission"[tw] OR "re admission"[tw] OR "Readmission*"[tw] OR "re admission*"[tw] OR "Readmit*"[tw] OR "re admit*"[tw] OR "unscheduled visit"[tw] OR "unscheduled visits"[tw] OR "unscheduled hospital visit"[tw] OR "unscheduled hospital visits"[tw] OR "Appointments and Schedules"[Mesh] OR "Emergency Service, Hospital"[Mesh] OR "Emergency Department"[tw] OR "Emergency Departments"[tw] OR "Emergency Hospital Service"[tw] OR "Emergency Hospital Services"[tw] OR "Emergency Outpatient Unit"[tw] OR "Emergency Outpatient Units"[tw] OR "Emergency Room"[tw] OR "Emergency Rooms"[tw] OR "Emergency Unit"[tw] OR "Emergency Units"[tw] OR "Emergency Ward"[tw] OR "Emergency Wards"[tw] OR "Hospital Emergency Service"[tw] OR "Hospital Emergency Services"[tw] OR "Hospitalization"[Mesh:noexp] OR "Hospitalization"[tw] OR "Hospitaliz*"[tw] OR "Hospitalisation"[tw] OR "hospitalis*"[tw] OR "major bleeding"[tw] OR "major bleed*"[tw] OR "major hemorrhage"[tw] OR "major hemorrhag*"[tw] OR "major haemorrhage"[tw] OR "major haemorrhag*"[tw] OR "Recurrence"[Mesh] OR "Recurrence"[tw] OR "Recurrent"[tw] OR "recurr*"[tw] OR "Mortality"[Mesh] OR "mortality"[Subheading] OR "Mortality"[tw] OR "mortalit*"[tw] OR "cause of death"[tw] OR "deaths"[tw] OR "survival rate"[tw] OR "survival rates"[tw] OR "Fatal Outcome"[mesh] OR "fatal outcome"[tw] OR "fatal outcomes"[tw]))

Embase

((exp *"lung embolism"/ OR "pulmonary embolism".ti,ab OR "pulmonary embolisms".ti,ab OR "pulmonary thromboembolisms".ti,ab OR "pulmonary thromboembolism".ti,ab OR "pulmonary thrombo embolism".ti,ab OR "pulmonary thromboembol*".ti,ab OR "pulmonary thrombo embol*".ti,ab) AND ("home treatment".ti,ab OR "home treat*".ti,ab OR "hometreatment".ti,ab OR "hometreat*".ti,ab OR "treatment at home".ti,ab OR exp *"Home Care"/ OR "home care".ti,ab OR "home therapy".ti,ab OR "home therap*".ti,ab OR "outpatient treatment".ti,ab OR "outpatient therapy".ti,ab OR "outpatient care".ti,ab OR "outpatient health service".ti,ab OR "outpatient health services".ti,ab OR exp *"Outpatient"/ OR "outpatient".ti,ab OR "outpatients".ti,ab OR "out of hospital treatment".ti,ab OR "out of hospital".ti,ab OR "outofhospital".ti,ab OR exp *"outpatient care"/ OR exp *"Ambulatory Care"/ OR "ambulant treatment".ti,ab OR "ambulatory treatment".ti,ab OR "ambulatory therapy".ti,ab OR "ambulatory care".ti,ab OR "early discharge".ti,ab OR "early discharges".ti,ab OR "early discharged".ti,ab OR "discharged early".ti,ab) NOT (("Case Report"/ OR "case report".ti OR exp "Review"/ OR "review".ti) NOT ("Clinical Study"/ OR exp "Clinical Trial"/ OR "trial".ti OR "RCT".ti OR exp "systematic review"/ OR "systematic review".ti)) NOT (conference review or conference abstract).pt)

Web of Science

((TI=("lung embolism" OR "pulmonary embolism" OR "pulmonary embolisms" OR "pulmonary thromboembolisms" OR "pulmonary thromboembolism" OR "pulmonary thrombo embolism" OR "pulmonary thromboembol*" OR "pulmonary thrombo embol*") OR AK=("lung embolism" OR "pulmonary embolism" OR "pulmonary embolisms" OR "pulmonary thromboembolisms" OR "pulmonary thromboembolism" OR "pulmonary thrombo embolism" OR "pulmonary thromboembol*" OR "pulmonary thrombo embol*") OR

AB=("lung embolism" OR "pulmonary embolism" OR "pulmonary embolisms" OR "pulmonary thromboembolisms" OR "pulmonary thromboembolism" OR "pulmonary thrombo embolism" OR "pulmonary thromboembol*" OR "pulmonary thrombo embol*")) AND (TI=("home treatment" OR "home treat*" OR "hometreatment" OR "hometreat*" OR "treatment at home" OR "Home Care" OR "home care" OR "home therapy" OR "home therap*" OR "outpatient treatment" OR "outpatient therapy" OR "outpatient care" OR "outpatient health service" OR "outpatient health services" OR "Outpatient" OR "outpatient" OR "outpatients" OR "out of hospital treatment" OR "out of hospital" OR "outofhospital" OR "outpatient care" OR "Ambulatory Care" OR "ambulant treatment" OR "ambulatory treatment" OR "ambulatory therapy" OR "ambulatory care" OR "early discharge" OR "early discharges" OR "early discharged" OR "discharged early") OR AK=("home treatment" OR "home treat*" OR "hometreatment" OR "hometreat*" OR "treatment at home" OR "Home Care" OR "home care" OR "home therapy" OR "home therap*" OR "outpatient treatment" OR "outpatient therapy" OR "outpatient care" OR "outpatient health service" OR "outpatient health services" OR "Outpatient" OR "outpatient" OR "outpatients" OR "out of hospital treatment" OR "out of hospital" OR "outofhospital" OR "outpatient care" OR "Ambulatory Care" OR "ambulant treatment" OR "ambulatory treatment" OR "ambulatory therapy" OR "ambulatory care" OR "early discharge" OR "early discharges" OR "early discharged" OR "discharged early") OR AB=("home treatment" OR "home treat*" OR "hometreatment" OR "hometreat*" OR "treatment at home" OR "Home Care" OR "home care" OR "home therapy" OR "home therap*" OR "outpatient treatment" OR "outpatient therapy" OR "outpatient care" OR "outpatient health service" OR "outpatient health services" OR "Outpatient" OR "outpatient" OR "outpatients" OR "out of hospital treatment" OR "out of hospital" OR "outofhospital" OR "outpatient care" OR "Ambulatory Care" OR "ambulant treatment" OR "ambulatory treatment" OR "ambulatory therapy" OR "ambulatory care" OR "early discharge" OR "early discharges" OR "early discharged" OR "discharged early")) NOT TI=(("Case Report" OR "case report" OR "Review" OR "review") NOT ("Clinical Study" OR "Clinical Trial" OR "trial" OR "RCT" OR "systematic review" OR "systematic review")) NOT DT=(meeting abstract))

Cochrane

(("lung embolism" OR "pulmonary embolism" OR "pulmonary embolisms" OR "pulmonary thromboembolisms" OR "pulmonary thromboembolism" OR "pulmonary thrombo embolism" OR "pulmonary thromboembol*" OR "pulmonary thrombo embol*"):ti,ab,kw AND ("home treatment" OR "home treat*" OR "hometreatment" OR "hometreat*" OR "treatment at home" OR "Home Care" OR "home care" OR "home therapy" OR "home therap*" OR "outpatient treatment" OR "outpatient therapy" OR "outpatient care" OR "outpatient health service" OR "outpatient health services" OR "Outpatient" OR "outpatient" OR "outpatients" OR "out of hospital treatment" OR "out of hospital" OR "outofhospital" OR "outpatient care" OR "Ambulatory Care" OR "ambulant treatment" OR "ambulatory treatment" OR "ambulatory therapy" OR "ambulatory care" OR "early discharge" OR "early discharges" OR "early discharged" OR "discharged early"):ti,ab,kw)

NOT DT=(meeting abstract)

Emcare

((exp *"lung embolism"/ OR "pulmonary embolism".ti,ab OR "pulmonary embolisms".ti,ab OR "pulmonary thromboembolisms".ti,ab OR "pulmonary thromboembolism".ti,ab OR "pulmonary thrombo embolism".ti,ab OR "pulmonary thromboembol*".ti,ab OR "pulmonary thrombo embol*".ti,ab) AND ("home treatment".ti,ab OR "home treat*".ti,ab OR "hometreatment".ti,ab OR "hometreat*".ti,ab OR "treatment at home".ti,ab OR exp *"Home Care"/ OR "home care".ti,ab OR "home therapy".ti,ab OR "home therap*".ti,ab OR "outpatient treatment".ti,ab OR "outpatient therapy".ti,ab OR "outpatient care".ti,ab OR "outpatient health service".ti,ab OR "outpatient health services".ti,ab OR exp *"Outpatient"/ OR "outpatient".ti,ab OR "outpatients".ti,ab OR "out of hospital treatment".ti,ab OR "out of hospital".ti,ab OR "outofhospital".ti,ab OR exp *"outpatient care"/ OR exp *"Ambulatory Care"/ OR "ambulant treatment".ti,ab OR "ambulatory treatment".ti,ab OR "ambulatory therapy".ti,ab OR "ambulatory care".ti,ab OR "early discharge".ti,ab OR "early discharges".ti,ab OR "early discharged".ti,ab OR "discharged early".ti,ab) NOT (("Case Report"/ OR "case report".ti OR exp "Review"/ OR "review".ti) NOT ("Clinical Study"/ OR exp "Clinical Trial"/ OR "trial".ti OR "RCT".ti OR exp "systematic review"/ OR "systematic review".ti)))

Academic Search Premier

((TI("lung embolism" OR "pulmonary embolism" OR "pulmonary embolisms" OR "pulmonary thromboembolisms" OR "pulmonary thromboembolism" OR "pulmonary thrombo embolism" OR "pulmonary thromboembol*" OR "pulmonary thrombo embol*") OR KW("lung embolism" OR "pulmonary embolism" OR "pulmonary embolisms" OR "pulmonary thromboembolisms" OR "pulmonary thromboembolism" OR "pulmonary thrombo embolism" OR "pulmonary thromboembol*" OR "pulmonary thrombo embol*") OR SU("lung embolism" OR "pulmonary embolism" OR "pulmonary embolisms" OR "pulmonary thromboembolisms" OR "pulmonary thromboembolism" OR "pulmonary thrombo embolism" OR "pulmonary thromboembol*" OR "pulmonary thrombo embol*") OR AB("lung embolism" OR "pulmonary embolism" OR "pulmonary embolisms" OR "pulmonary thromboembolisms" OR "pulmonary thromboembolism" OR "pulmonary thrombo embolism" OR "pulmonary thromboembol*" OR "pulmonary thrombo embol*"))

AND

(TI("home treatment" OR "home treat*" OR "hometreatment" OR "hometreat*" OR "treatment at home" OR "Home Care" OR "home care" OR "home therapy" OR "home therap*" OR "outpatient treatment" OR "outpatient therapy" OR "outpatient care" OR "outpatient health service" OR "outpatient health services" OR "Outpatient" OR "outpatient" OR "outpatients" OR "out of hospital treatment" OR "out of hospital" OR "outofhospital" OR "outpatient care" OR "Ambulatory Care" OR "ambulant treatment" OR "ambulatory treatment" OR "ambulatory therapy" OR "ambulatory care" OR "early discharge" OR "early discharges" OR "early discharged" OR "discharged early") OR KW("home treatment" OR "home treat*" OR "hometreatment" OR "hometreat*" OR "treatment at home" OR "Home Care" OR "home care" OR "home therapy" OR "home therap*" OR "outpatient treatment" OR "outpatient therapy" OR "outpatient care" OR "outpatient health service" OR "outpatient health services" OR "Outpatient" OR "outpatient" OR "outpatients" OR "out of hospital treatment" OR "out of hospital" OR "outofhospital" OR "outpatient care" OR "Ambulatory Care" OR "ambulant treatment" OR "ambulatory treatment" OR "ambulatory therapy" OR "ambulatory care" OR "early discharge" OR "early discharges" OR "early discharged" OR "discharged early") OR SU("home treatment" OR "home treat*" OR "hometreatment" OR "hometreat*" OR "treatment at home" OR "Home Care" OR "home care" OR "home therapy" OR "home therap*" OR "outpatient treatment" OR "outpatient therapy" OR "outpatient care" OR "outpatient health service" OR "outpatient health services" OR "Outpatient" OR "outpatient" OR "outpatients" OR "out of hospital treatment" OR "out of hospital" OR "outofhospital" OR "outpatient care" OR "Ambulatory Care" OR "ambulant treatment" OR "ambulatory treatment" OR "ambulatory therapy" OR "ambulatory care" OR "early discharge" OR "early discharges" OR "early discharged" OR "discharged early")) OR AB("home treatment" OR "home treat*" OR "hometreatment" OR "hometreat*" OR "treatment at home" OR "Home Care" OR "home care" OR "home therapy" OR "home therap*" OR "outpatient treatment" OR "outpatient therapy" OR "outpatient care" OR "outpatient health service" OR "outpatient health services" OR "Outpatient" OR "outpatient" OR "outpatients" OR "out of hospital treatment" OR "out of hospital" OR "outofhospital" OR "outpatient care" OR "Ambulatory Care" OR "ambulant treatment" OR "ambulatory treatment" OR "ambulatory therapy" OR "ambulatory care" OR "early discharge" OR "early discharges" OR "early discharged" OR "discharged early"))

NOT TI("Case Report" OR "case report" OR "Review" OR "review"))

WHO Covid-19 database

(("lung embolism" OR "pulmonary embolism" OR "pulmonary embolisms" OR "pulmonary thromboembolisms" OR "pulmonary thromboembolism" OR "pulmonary thrombo embolism" OR "pulmonary thromboembol*" OR "pulmonary thrombo embol*") AND ("home treatment" OR "home treat*" OR "hometreatment" OR "hometreat*" OR "treatment at home" OR "Home Care" OR "home care" OR "home therapy" OR "home therap*" OR "outpatient treatment" OR "outpatient therapy" OR "outpatient care" OR "outpatient health service" OR "outpatient health services" OR "Outpatient" OR "outpatient" OR "outpatients" OR "out of hospital treatment" OR "out of hospital" OR "outofhospital" OR "outpatient care" OR "Ambulatory Care" OR "ambulant treatment" OR "ambulatory treatment" OR "ambulatory therapy" OR "ambulatory care" OR "early discharge" OR "early discharges" OR "early discharged" OR "discharged early"))

Google Scholar

"pulmonary embolism"|"pulmonary thromboembolism" "home treatment"|"home therapy"|"Home Care"|"outpatient"|"out of hospital"|"ambulatory care"|"early discharge"|"discharged early" -"case" -"review"

Appendix B: variables IPDMA home treatment

Variable name	Short description	Level/value 1 [code]	Level/value 2 [code]	Level/value 3 [code]	Level/value 4 [code]	Level/value 5 [code]	Definition	Difference in definitions used
IPDMA_ID	IPDMA ID							
Study_ID	Specific ID from the origional study							
stratification_tool	Which tool was used to select eligibile patient for home-treatment	Negative Hestia (like) criteria [1]	negative sPESI/PESI [2]	Other + no RV overload [3]	Negative sPESI + no RV overload [4]	Negative Hestia + no RV overload [5]		
Study	Patient is derived from which study and which algorithm was used to select low risk patients?	101=hestia, 1021=vesta_hestia, 1022=Vesta_hestia_ntprobnp, 1031=HOME-PE_hestia, 1032=HOME-PE_spesi 104=MATH_VTE_unkown, 1041=MATHVTE_hestia, 1042=MATH-VTE_Spesi, 106=HoT_PE_hestia, 107=Kabrhel_other, 109=Otero_other, 110=Font_other, 111=Vinson_PESI, 112=Bledsoe_PESI						
Study2	Patient is derived from which study?	101=hestia, 102-vesta, 103=HOME-PE 104=MATH_VTE, 106=HoT_PE, 107=Kabrhel, 109=Otero, 110=Font, 111=Vinson, 112=Bledsoe						
age	Age in years							
agegroup	what age group is the patient in?	18-40 [1]	41-60 [2]	61-80 [3]	>80 [4]			
age18	Age 18-40 years	no [0]	yes [1]					
age41	Age 41-60 years	no [0]	yes [1]					
age61	Age 61-80 years	no [0]	yes [1]					
age80	Age >80 years	no [0]	yes [1]					
Sex	Sex	male [1]	female [2]					
pulm	Preexisting pulmonary disease	no [0]	yes [1]				History of chronic obstructive pulmonary disease, asthma, or lung fibrosis.	Zondag et al. and Exter et al. COPD with medication [equal to copd]; Kline et al. chronic lung disease; Font et al. COPD

cvd	Preexisting cardiovascular disease	no [0]	yes [1]				Please include any of coronary artery disease, heart failure, congenital heart disease, cardiomyopathy, rheumatic heart disease. Do not include hypertension	Zondag et al. and Exter et al. heart faillure with medication [equal to chf]; Vinson et al. Congestive heart faillure [equal to chf]; Font et al. heart condition
carpulm_calc	Preexisting cardiopulmonary disease	no [0]	yes [1]				Preexisting pulmonary disease or congestive heart faillure; if chf=1 or pulm=1	Zondag et al. and Exter et al. COPD with medication [equal to copd]; Kline et al. chronic lung disease; Font et al. COPD
renal60	Decreased kidney function eGFR < 60 ml/min	no [0]	yes [1]				eGFR < 60 ml/min according to the Cockcroft-Gault equation.	
estrogen	Exogenous estrogen use	no [0]	yes [1]				all hormonal therapy including but not limiting to: contracepitves containing estrogen, hormonal replacement therapy	Zondag et al. and Exter et al. Estrogen use
immosurg	Recent (<4 weeks) immobilization or surgery	no [0]	yes [1]					
prevvte	Previous DVT or PE	no [0]	yes [1]				Previous DVT or PE; if prevdvt=1 OR prevpe=1	
mal	active malignancy	no [0]	yes [1]				Indication of a diagnosis of active cancer (not including skin cancer basalcel carcinoma and squamouscell carcinoma) for the participant, based on meeting at least one of the following criteria: - Current diagnosis of cancer - Receiving treatment for cancer - Not receiving treatment for cancer and not in complete response;	
malhist	History of cancer	no [0]	yes [1]				Malignancy which is not active not including basalcellcarcinoma or squamouscellcarcinoma	Roy et al. has the patient ever had cancer whether it is active or not.
date	Date of PE event i.e. this is the date of the imaging diagnosis.						Date of PE event i.e. this is the date of the imaging diagnosis.	
symp	incidental or symptomatic PE	symptomatic [1]	incidental [2]				Acute symptomatic PE is defined as radiological confirmation of PE on a test ordered to diagnose PE. Incidental PE is defined as radiological confirmation of PE on a test ordered for any other reason than suspected VTE. Radiological diagnosis criteria for PE are as follows: (1) Computed Tomography Pulmonary Angiography (CTPA): PE is defined as a central contrast filling defect or complete occlusion up to the subsegmental level of the pulmonary arteries. (2) Ventilation/Perfusion Scan (V/Q): PE is defined as at least two large mismatched segmental perfusion	

							defects or the arithmetic equivalent in moderate or large and moderate defects (revised PIOPED criteria). (3) Digital Subtraction Angiography (DSA): PE is defined as a filling defect or a cut-off of a vessel of > 2.5 mm	
hr110	Heart rate ≥110 bpm	no [0]	yes [1]					
hr100	Heart rate ≥100 bpm	no [0]	yes [1]					
bpsyst100	Systolic blood pressure <100 mmHg	no [0]	yes [1]					
resp30	respiratory rate of ≥ 30 breaths/min	no [0]	yes [1]					
sat90	Oxygen saturation <90% or need for oxygen suppletion	no [0]	yes [1]					
ntprobnoabn500	NT-proBNP/BNP level abnormal>500 ng/L or BNP>100	no [0]	yes [1]					
tropoabn	Abnormal troponine	no [0]	yes [1]				Troponin level >99th percentile according to local technique;	Exter et al. hsTNT >0.014 ug/L; Kline et al. TnI with 0.1 ng/mL as cutoff; Bledsoe et al. Troponin I > 0.04 ng/mL; Kabhrel et al. Troponin T >0.1 ng/ml
location	the most proximal location of a pulmonary embolism	central [1]	lobar [2]	segmental [3]	subsegmental [4]		subsegmental: The first arterial branch division of any segmental artery independent of artery diameter, visible in at least two subsequent axial slices, using a CT scanner with a desired maximum collimator width of ≤1mm	Zondag et al. no difference between central/lobair, all patients with central/lobair are classified in this study as central
rvlvratio9	RV/LV ratio according to CTPA > 0.9	no [0]	yes [1]					
treat.med.bin	VKA/LMWH or DOAC	Direct oral anticoagulant [1]	vitamin K antagonist	low molecular weight heparin [3]				
treat.loca	patient treated at home according to original arm study	no [0]	yes [1]					
treat.loca24	patient treated at home or in the hospital within 24 hours	Home [1]	hospital [2]				home treatment is when a patient is discharched within 24 hours after diagnosis of PE, or randomization, or emergency department registration	
censored	Lost to follow-up	study complete [0]	death [1]	lost to follow-up [2]	withdrawal from study [3]			

censored.date	Date censored							
fuptime	number of days follow-up duration (=from date of PE diagnosis until date of last follow-up visit)							
-	-	-	-	-	-	-	-	-
vtefup	All VTE events during follow-up. Including but not limited to: DVT, nonfatal PE, and/or fatal PE	no [0]	yes [1]					Zondag et al. Recurrent VTE was considered to be present if recurrent PE or DVT were documented objectively, or in the case of death for which PE could not be confidently ruled out as a contributory cause.<br>Vinson et al. new or expanded abnormality on imaging;<br>Kline et al. The definitions of new or recurrent VTE were based upon chart review to confirm suspected recurrent PE or DVT, and the requirement of explicit radiographic or ultrasonic evidence of PE/DVT;<br>Bledsoe et al. new abnormality on a relevant imaging study (CUS, CTPA, and ventilation/perfusion scan). Imaging was performed by treating clinicians to evaluate symptoms suspicious for new VTE; there was no routine imaging of asymptomatic patients during follow-up;<br>Font et al. a second venous thrombotic event or a clinically relevant progression of the previous embolism during anticoagulation<br><br>Use of an adjudication committee: Barco et al, Roy et al., Zondag et al. Font et al. Exter et al.
vtefup.14	All VTE events during 14-day follow-up. Including but not limited to: DVT, nonfatal PE, and/or fatal PE	no [0]	yes [1]				VTE during <14 days of follow-up; Day of PE diagnosis= day 0	
vtefup.28	All VTE events during 30day follow-up. Including but not limited to: DVT, nonfatal PE, and/or fatal PE; KABHREL follow-up of 28 days	no [0]	yes [1]					
vtefup.30	All VTE events during 1 month follow-up. Including but not limited to: DVT, nonfatal PE, and/or fatal PE	no [0]	yes [1]				VTE during <30 days of follow-up; Day of PE diagnosis= day 0	
vtefup.90	All VTE events during 3-month follow-up. Including but not limited to: DVT, nonfatal PE, and/or fatal PE	no [0]	yes [1]				VTE during <90 days of follow-up; Day of PE diagnosis= day 0	
mbfup	major bleeding during follow-up	no [0]	yes [1]				1) fatal bleeding, 2) symptomatic bleeding in a critical area or organ,	Font et al: A bleeding event was classified as major if it was associated with death, occurred at a critical

							and/or 3) bleeding causing a fall in hemoglobin level of ≥ 1.24 mmol/L, or leading to a transfusion of ≥ 2 units of blood	site (intracranial, intraspinal, intraocular, retroperitoneal, or pericardial), required a blood transfusion, or resulted in a reduction of hemoglobin of at least 20 g/L<br><br>Use of an adjudication committee: Barco et al, Font et al, Exter et al., Roy et al. Zondag et al.
mbfup.14	major bleeding during 14-day follow-up after acute PE	no [0]	yes [1]				MB during <14 days of follow-up; Day of PE diagnosis= day 0	
mbfup.28	major bleeding during 1-month follow-up after acute PE; Kabhrel 28 days	no [0]	yes [1]					
mbfup.30	major bleeding during 1-month follow-up after acute PE	no [0]	yes [1]				MB during <30 days of follow-up; Day of PE diagnosis= day 0	
mbfup.90	major bleeding during 3-month follow-up after acute PE	no [0]	yes [1]				MB during <90 days of follow-up; Day of PE diagnosis= day 0	
nonmbfup	Clinically relevant non-major bleeding during follow-up	no [0]	yes [1]				Clinically relevant non-major bleeding is defined as any sign or symptom of hemorrhage that does not meet criteria for major bleeding but does meet at least one of the following criteria: (a) Requiring medical intervention by a healthcare professional (b) Leading to hospitalization or an increased level of care (c) Prompting a face to face (i.e., not just a telephone or electronic communication) evaluation	Zondag et al. Clinically relevant bleeding episodes, not qualifying as major bleeding, were classified as clinically relevant non-major bleeding (e.g. epistaxis that required intervention, large hematoma visible on the skin, or spontaneous macroscopic hematuria); Kline et al. patient-reported bleeding requiring any unscheduled medical care<br><br>Use of an adjudication committee: Barco et al
nonmbfup.14	Clinically relevant non-major bleeding during 14 days follow-up after acute PE	no [0]	yes [1]				nonMB during <14 days of follow-up; Day of PE diagnosis= day 0	
nonmbfup.28	Clinically relevant non-major bleeding during 1 month of follow-up after acute PE; kabhrel 28 days	no [0]	yes [1]					
nonmbfup.30	Clinically relevant non-major bleeding during 1 month of follow-up after acute PE	no [0]	yes [1]				nonMB during <30 days of follow-up; Day of PE diagnosis= day 0	
nonmbfup.90	Clinically relevant non-major bleeding during 3 month of follow-up after acute PE	no [0]	yes [1]				nonMB during <90 days of follow-up; Day of PE diagnosis= day 0	
deathfup	death during follow-up	no [0]	yes [1]					Vinson et al: to identify death, we used a health system mortality database that links to the Social Security death master file and the California State Department of Vital Statistics, which include deaths outside the delivery system. We also identified out-of-system medical encounters using comprehensive claims data to improve the accuracy of our 5-day and 30-day outcomes

								Use of an adjudication committee: Barco et al, Exter et al., Font et al, Roy et al. Zondag et al.
deathfup.vtemb	Cause of death	PE-related death; category A [1]	Undetermined cause of death; category B [2]	Major bleeding (MB) related death; category C [3]	Cause of death other than PE or MB; category D [4]		Category A includes A1: autopsy-confirmed PE in the absence of another more likely cause of death; A2: objectively confirmed PE before death(= ≥1 of the following situations in the last 48 hours before death: PE diagnosed by imaging or Objectively confirmed proximal deep vein thrombosis of the lower extremity in patients with clinical signs and symptoms of PE) in the absence of another more likely cause of death; and A3: PE is not objectively confirmed, but is most likely the main cause of death.<br>Category B includes B1: cause of death is undetermined, despite available information; and B2: insufficient clinical information available to determine the cause of death.<br>Category C includes C1: autopsy-confirmed MB in the absence of another more likely cause of death; C2: objectively confirmed MB in the last 48 hours before death (see MB definition in MB variable) in the absence of another more likely cause of death; and C3: MB is not objectively confirmed, but is most likely the main cause of death.<br>Category D included all other causes other than PE or MB	
deathfup.14	death during 14-days follow-up after acute PE	no [0]	yes [1]				death during <14 days of follow-up; Day of PE diagnosis= day 0	
deathfup.28	death during 1-month follow-up after acute PE; Kabhrel 28 days	no [0]	yes [1]					
deathfup.30	death during 1-month follow-up after acute PE	no [0]	yes [1]				death during <30 days of follow-up; Day of PE diagnosis= day 0	
deathfup.90	death during 3-month follow-up after acute PE	no [0]	yes [1]				death during <90 days of follow-up; Day of PE diagnosis= day 0	
combfup	Recurrent VTE, major bleeding or all-cause mortality during follow up	no [0]	yes [1]					
combfup.14	Recurrent VTE, major bleeding or all-cause mortality during 14 days follow-up after acute PE	no [0]	yes [1]				Recurrent VTE, major bleeding or all-cause mortalit during <14 days of follow-up; Day of PE diagnosis= day 0	
combfup.28	Recurrent VTE, major bleeding bleeding or all-cause mortality	no [0]	yes [1]					

	during follow up during 1-month follow-up after acute PE; kabhrel 28 days							
combfup.30	Recurrent VTE, major bleeding or all-cause mortality during 1 month of follow-up after acute PE	no [0]	yes [1]				Recurrent VTE, major bleeding or all-cause mortalit during <30 days of follow-up; Day of PE diagnosis= day 0	
combfup.90	Recurrent VTE, major bleeding or all-cause mortality during 3-month follow-up after acute PE	no [0]	yes [1]				Recurrent VTE, major bleeding or all-cause mortalit during <90 days of follow-up; Day of PE diagnosis= day 0	

Appendix C: definitions of subgroups

The following subgroups were evaluated, based on the presence or absence of the following characteristics: symptomatic versus incidental PE, the latter defined as radiological confirmation of PE on a test ordered for any reason other than suspected VTE[1]; cancer (not including basal cell or squamous cell carcinoma), defined as meeting at least one of the following criteria: (1) current diagnosis of cancer, (2) receiving treatment for cancer or (3) not receiving treatment for cancer and not in complete remission[1]; decreased kidney function, defined as an estimated glomerular filtration rate (eGFR) of <60 ml/min; preexisting pulmonary disease, defined as a history of chronic obstructive pulmonary disease, asthma, or lung fibrosis; preexisting cardiovascular disease, defined as any history of coronary artery disease, heart failure, congenital heart disease, cardiomyopathy or rheumatic heart disease; abnormal troponin defined as a troponin level >99th percentile according to local assay; abnormal (NT-pro)BNP, defined as NT-proBNP > 500 ng/L, or BNP >100 ng/L; RV overload defined as a right ventricle (RV)/left ventricle (LV) ratio >0·9 on CTPA or echocardiogram; and RV dysfunction, defined by the 2019 European Society of Cardiology (ESC) Guidelines for acute pulmonary embolism.[2]

We chose cut-off values that were most frequently used within the original studies to define abnormal NT-proBNP (> 500 ng/L) and RV overload (RV/LV ratio >0·9) as these cut-offs resulted in the lowest frequency of missing data and thus the highest power to evaluate subgroup outcomes.

Appendix D: supplementary tables and figures

Table S1: description of missing data

Yellow marking means variable was not imputed because the variable was systematically missing within a study. Green marking means study was not included in the subgroup analysis because of single value within a study. Grey marking means excluded from subgroup analysis, since one of the imputed dataset included a single value within a study. Red marked the imputed variables from a specific study while being >30% missing within that study.

Variables	**Zondag et al.[3,4]**	**Exter et al. [5]**	**Roy et al.[6]**	**Kline et al.[7]**	**Barco et al. [8]**	**Kabrhel et al. [9]**	**Otero et al.[10]**	**Font et al. [11]**	**Vinson et al.[12]**	**Bledsoe et al. [13]**	**Overall % of missing**
**Description of missing data in patients that received home treatment according to the IPDMA definition of home treatment (discharge within 24 hours) (n=2756)**											
n	296	513	681	604	170	122	0	62	116	192	
age (mean (SD))	54.47 (15.36)	53.48 (14.65)	56.44 (16.22)	51.97 (16.57)	54.46 (16.02)	55.41 (16.43)	-	62.48 (10.33)	60.25 (15.05)	44.35 (14.32)	0.0
sex = female (%)	124 (41.9)	235 (45.8)	314 (46.1)	301 (49.8)	80 (47.1)	64 (52.5)	-	25 (40.3)	61 (52.6)	103 (53.6)	0.0
symp (%)							-				37.5
Symptomatic	296 (100.0)	513 (100.0)	681 (100.0)	0 (0.0)	155 (91.2)	0 (0.0)	-	14 (22.6)	0 (0.0)	0 (0.0)	
Incidental	0 (0.0)	0 (0.0)	0 (0.0)	0 (0.0)	15 (8.8)	0 (0.0)	-	48 (77.4)	0 (0.0)	0 (0.0)	
NA	0 (0.0)	0 (0.0)	0 (0.0)	604 (100.0)	0 (0.0)	122 (100.0)	-	0 (0.0)	116 (100.0)	192 (100.0)	
treat.med.bin (%)							-				5.8
DOAC	0 (0.0)	0 (0.0)	541 (79.4)	604 (100.0)	170 (100.0)	42 (34.4)	-	0 (0.0)	0 (0.0)	165 (85.9)	
VKA/LWMH	296 (100.0)	507 (98.8)	104 (15.3)	0 (0.0)	0 (0.0)	78 (63.9)	-	62 (100.0)	0 (0.0)	27 (14.1)	
NA	0 (0.0)	6 (1.2)	36 (5.3)	0 (0.0)	0 (0.0)	2 (1.6)	-	0 (0.0)	116 (100.0)	0 (0.0)	
mal (%)							-				0.7
No	268 (90.5)	474 (92.4)	621 (91.2)	580 (96.0)	150 (88.2)	67 (54.9)	-	0 (0.0)	100 (86.2)	190 (99.0)	
Yes	28 (9.5)	33 (6.4)	49 (7.2)	24 (4.0)	19 (11.2)	55 (45.1)	-	62 (100.0)	16 (13.8)	2 (1.0)	
NA	0 (0.0)	6 (1.2)	11 (1.6)	0 (0.0)	1 (0.6)	0 (0.0)	-	0 (0.0)	0 (0.0)	0 (0.0)	
prevvte (%)							-				8.4

Variables	Zondag et al.[3,4]	Exter et al. [5]	Roy et al.[6]	Kline et al.[7]	Barco et al. [8]	Kabrhel et al. [9]	Otero et al.[10]	Font et al. [11]	Vinson et al.[12]	Bledsoe et al. [13]	Overall % of missing
No	222 (75.0)	389 (75.8)	505 (74.2)	171 (28.3)	135 (79.4)	88 (72.1)	-	58 (93.5)	0 (0.0)	155 (80.7)	
Yes	74 (25.0)	118 (23.0)	165 (24.2)	336 (55.6)	33 (19.4)	34 (27.9)	-	4 (6.5)	0 (0.0)	37 (19.3)	
NA	0 (0.0)	6 (1.2)	11 (1.6)	97 (16.1)	2 (1.2)	0 (0.0)	-	0 (0.0)	116 (100.0)	0 (0.0)	
renal60 (%)							-				21.6
No	91 (30.7)	456 (88.9)	520 (76.4)	435 (72.0)	158 (92.9)	105 (86.1)	-	57 (91.9)	0 (0.0)	177 (92.2)	
Yes	3 (1.0)	21 (4.1)	53 (7.8)	53 (8.8)	12 (7.1)	11 (9.0)	-	2 (3.2)	0 (0.0)	6 (3.1)	
NA	202 (68.2)	36 (7.0)	108 (15.9)	116 (19.2)	0 (0.0)	6 (4.9)	-	3 (4.8)	116 (100.0)	9 (4.7)	
carpulm_calc (%)							-				44.1
No	284 (95.9)	482 (94.0)	0 (0.0)	0 (0.0)	157 (92.4)	92 (75.4)	-	45 (72.6)	88 (75.9)	160 (83.3)	
Yes	12 (4.1)	24 (4.7)	36 (5.3)	42 (7.0)	12 (7.1)	30 (24.6)	-	17 (27.4)	28 (24.1)	32 (16.7)	
NA	0 (0.0)	7 (1.4)	645 (94.7)	562 (93.0)	1 (0.6)	0 (0.0)	-	0 (0.0)	0 (0.0)	0 (0.0)	
tropoabn (%)							-				53.2
No	0 (0.0)	284 (55.4)	339 (49.8)	246 (40.7)	0 (0.0)	73 (59.8)	-	0 (0.0)	11 (9.5)	190 (99.0)	
Yes	0 (0.0)	44 (8.6)	82 (12.0)	19 (3.1)	0 (0.0)	1 (0.8)	-	0 (0.0)	0 (0.0)	1 (0.5)	
NA	296 (100.0)	185 (36.1)	260 (38.2)	339 (56.1)	170 (100.0)	48 (39.3)	-	62 (100.0)	105 (90.5)	1 (0.5)	
ntprobnpabn500 (%)							-				51.3
No	0 (0.0)	450 (87.7)	274 (40.2)	122 (20.2)	117 (68.8)	53 (43.4)	-	0 (0.0)	36 (31.0)	183 (95.3)	
Yes	0 (0.0)	23 (4.5)	30 (4.4)	27 (4.5)	5 (2.9)	5 (4.1)	-	0 (0.0)	12 (10.3)	6 (3.1)	
NA	296 (100.0)	40 (7.8)	377 (55.4)	455 (75.3)	48 (28.2)	64 (52.5)	-	62 (100.0)	68 (58.6)	3 (1.6)	
rvlvratio9 (%)							-				61.4
No	87 (29.4)	0 (0.0)	477 (70.0)	0 (0.0)	155 (91.2)	4 (3.3)	-	39 (62.9)	0 (0.0)	0 (0.0)	

Variables	Zondag et al.[3,4]	Exter et al. [5]	Roy et al.[6]	Kline et al.[7]	Barco et al. [8]	Kabrhel et al. [9]	Otero et al.[10]	Font et al. [11]	Vinson et al.[12]	Bledsoe et al. [13]	Overall % of missing
Yes	187 (63.2)	0 (0.0)	85 (12.5)	0 (0.0)	9 (5.3)	2 (1.6)	-	20 (32.3)	0 (0.0)	0 (0.0)	
NA	22 (7.4)	513 (100.0)	119 (17.5)	604 (100.0)	6 (3.5)	116 (95.1)	-	3 (4.8)	116 (100.0)	192 (100.0)	
echo.rvdys (%)							-				85.0
No	0 (0.0)	0 (0.0)	0 (0.0)	34 (5.6)	170 (100.0)	8 (6.6)	-	0 (0.0)	3 (2.6)	190 (99.0)	
Yes	0 (0.0)	0 (0.0)	0 (0.0)	4 (0.7)	0 (0.0)	1 (0.8)	-	0 (0.0)	1 (0.9)	2 (1.0)	
NA	296 (100.0)	513 (100.0)	681 (100.0)	566 (93.7)	0 (0.0)	113 (92.6)	-	62 (100.0)	112 (96.6)	0 (0.0)	
vtefup.14 (%)							-				1.3
No	292 (98.6)	512 (99.8)	676 (99.3)	598 (99.0)	169 (99.4)	97 (79.5)	-	59 (95.2)	116 (100.0)	192 (100.0)	
Yes	3 (1.0)	0 (0.0)	1 (0.1)	5 (0.8)	0 (0.0)	0 (0.0)	-	1 (1.6)	0 (0.0)	0 (0.0)	
NA	1 (0.3)	1 (0.2)	4 (0.6)	1 (0.2)	1 (0.6)	25 (20.5)	-	2 (3.2)	0 (0.0)	0 (0.0)	
mbfup.14 (%)							-				1.4
No	295 (99.7)	511 (99.6)	675 (99.1)	603 (99.8)	169 (99.4)	93 (76.2)	-	58 (93.5)	116 (100.0)	192 (100.0)	
Yes	1 (0.3)	1 (0.2)	3 (0.4)	0 (0.0)	0 (0.0)	0 (0.0)	-	1 (1.6)	0 (0.0)	0 (0.0)	
NA	0 (0.0)	1 (0.2)	3 (0.4)	1 (0.2)	1 (0.6)	29 (23.8)	-	3 (4.8)	0 (0.0)	0 (0.0)	
nonmbfup.14 (%)							-				44.0
No	0 (0.0)	0 (0.0)	671 (98.5)	596 (98.7)	165 (97.1)	91 (74.6)	-	0 (0.0)	0 (0.0)	0 (0.0)	
Yes	0 (0.0)	0 (0.0)	6 (0.9)	6 (1.0)	4 (2.4)	4 (3.3)	-	0 (0.0)	0 (0.0)	0 (0.0)	
NA	296 (100.0)	513 (100.0)	4 (0.6)	2 (0.3)	1 (0.6)	27 (22.1)	-	62 (100.0)	116 (100.0)	192 (100.0)	
deathfup.14 (%)							-				1.1
No	295 (99.7)	512 (99.8)	677 (99.4)	603 (99.8)	169 (99.4)	97 (79.5)	-	60 (96.8)	116 (100.0)	192 (100.0)	
Yes	1 (0.3)	1 (0.2)	1 (0.1)	0 (0.0)	0 (0.0)	0 (0.0)	-	2 (3.2)	0 (0.0)	0 (0.0)	

Variables	Zondag et al.[3,4]	Exter et al. [5]	Roy et al.[6]	Kline et al.[7]	Barco et al. [8]	Kabrhel et al. [9]	Otero et al.[10]	Font et al. [11]	Vinson et al.[12]	Bledsoe et al. [13]	Overall % of missing
NA	0 (0.0)	0 (0.0)	3 (0.4)	1 (0.2)	1 (0.6)	25 (20.5)	-	0 (0.0)	0 (0.0)	0 (0.0)	
combfup.14 (%)							-				1.3
No	292 (98.6)	511 (99.6)	674 (99.0)	598 (99.0)	169 (99.4)	93 (76.2)	-	57 (91.9)	116 (100.0)	192 (100.0)	
Yes	4 (1.4)	2 (0.4)	4 (0.6)	5 (0.8)	0 (0.0)	0 (0.0)	-	3 (4.8)	0 (0.0)	0 (0.0)	
NA	0 (0.0)	0 (0.0)	3 (0.4)	1 (0.2)	1 (0.6)	29 (23.8)	-	2 (3.2)	0 (0.0)	0 (0.0)	
immosurg (%)							-				15.0
No	268 (90.5)	437 (85.2)	582 (85.5)	546 (90.4)	141 (82.9)	105 (86.1)	-	0 (0.0)	0 (0.0)	0 (0.0)	
Yes	27 (9.1)	68 (13.3)	65 (9.5)	54 (8.9)	25 (14.7)	17 (13.9)	-	7 (11.3)	0 (0.0)	0 (0.0)	
NA	1 (0.3)	8 (1.6)	34 (5.0)	4 (0.7)	4 (2.4)	0 (0.0)	-	55 (88.7)	116 (100.0)	192 (100.0)	
estrogen (%)							-				9.9
No	248 (83.8)	416 (81.1)	607 (89.1)	563 (93.2)	138 (81.2)	114 (93.4)	-	54 (87.1)	55 (47.4)	0 (0.0)	
Yes	47 (15.9)	91 (17.7)	63 (9.3)	40 (6.6)	31 (18.2)	8 (6.6)	-	8 (12.9)	0 (0.0)	0 (0.0)	
NA	1 (0.3)	6 (1.2)	11 (1.6)	1 (0.2)	1 (0.6)	0 (0.0)	-	0 (0.0)	61 (52.6)	192 (100.0)	
tropongml (mean (SD))	NaN (NA)	0.01 (0.03)	1.12 (5.78)	0.50 (2.23)	0.19 (1.73)	0.01 (0.01)	-	NaN (NA)	0.02 (0.00)	0.01 (0.01)	47.8
hr110 (%)							-				6.4
No	235 (79.4)	478 (93.2)	608 (89.3)	536 (88.7)	166 (97.6)	0 (0.0)	-	62 (100.0)	101 (87.1)	174 (90.6)	
Yes	24 (8.1)	35 (6.8)	62 (9.1)	62 (10.3)	3 (1.8)	0 (0.0)	-	0 (0.0)	15 (12.9)	18 (9.4)	
NA	37 (12.5)	0 (0.0)	11 (1.6)	6 (1.0)	1 (0.6)	122 (100.0)	-	0 (0.0)	0 (0.0)	0 (0.0)	
bpsyst100 (%)							-				6.4
No	259 (87.5)	513 (100.0)	665 (97.7)	588 (97.4)	170 (100.0)	0 (0.0)	-	62 (100.0)	110 (94.8)	187 (97.4)	
Yes	1 (0.3)	0 (0.0)	4 (0.6)	10 (1.7)	0 (0.0)	0 (0.0)	-	0 (0.0)	6 (5.2)	5 (2.6)	

Variables	Zondag et al.[3,4]	Exter et al. [5]	Roy et al.[6]	Kline et al.[7]	Barco et al. [8]	Kabrhel et al. [9]	Otero et al.[10]	Font et al. [11]	Vinson et al.[12]	Bledsoe et al. [13]	Overall % of missing
NA	36 (12.2)	0 (0.0)	12 (1.8)	6 (1.0)	0 (0.0)	122 (100.0)	-	0 (0.0)	0 (0.0)	0 (0.0)	
resp30 (%)							-				62.0
No	0 (0.0)	0 (0.0)	0 (0.0)	591 (97.8)	141 (82.9)	0 (0.0)	-	0 (0.0)	112 (96.6)	190 (99.0)	
Yes	0 (0.0)	0 (0.0)	0 (0.0)	5 (0.8)	2 (1.2)	0 (0.0)	-	0 (0.0)	4 (3.4)	2 (1.0)	
NA	296 (100.0)	513 (100.0)	681 (100.0)	8 (1.3)	27 (15.9)	122 (100.0)	-	62 (100.0)	0 (0.0)	0 (0.0)	
sat90 (%)							-				7.2
No	233 (78.7)	509 (99.2)	644 (94.6)	595 (98.5)	159 (93.5)	0 (0.0)	-	62 (100.0)	111 (95.7)	192 (100.0)	
Yes	17 (5.7)	4 (0.8)	23 (3.4)	2 (0.3)	1 (0.6)	0 (0.0)	-	0 (0.0)	5 (4.3)	0 (0.0)	
NA	46 (15.5)	0 (0.0)	14 (2.1)	7 (1.2)	10 (5.9)	122 (100.0)	-	0 (0.0)	0 (0.0)	0 (0.0)	
creat (mean (SD))	107.56 (31.42)	113.46 (37.64)	103.69 (36.58)	116.24 (52.36)	83.00 (18.18)	91.11 (20.33)	-	NaN (NA)	NaN (NA)	89.64 (18.63)	23.8
location (%)							-				70.7
Central	31 (10.5)	0 (0.0)	0 (0.0)	22 (3.6)	0 (0.0)	6 (4.9)	-	11 (17.7)	15 (12.9)	0 (0.0)	
Lobar	0 (0.0)	0 (0.0)	0 (0.0)	94 (15.6)	0 (0.0)	23 (18.9)	-	26 (41.9)	27 (23.3)	0 (0.0)	
Segmental	89 (30.1)	0 (0.0)	0 (0.0)	126 (20.9)	0 (0.0)	60 (49.2)	-	15 (24.2)	38 (32.8)	84 (43.8)	
Subsegmental	22 (7.4)	0 (0.0)	0 (0.0)	41 (6.8)	0 (0.0)	23 (18.9)	-	5 (8.1)	16 (13.8)	33 (17.2)	
NA	154 (52.0)	513 (100.0)	681 (100.0)	321 (53.1)	170 (100.0)	10 (8.2)	-	5 (8.1)	20 (17.2)	75 (39.1)	

Variables	Zondag et al.[3,4]	Exter et al. [5]	Roy et al.[6]	Kline et al.[7]	Barco et al. [8]	Kabrhel et al. [9]	Otero et al.[10]	Font et al. [11]	Vinson et al.[12]	Bledsoe et al. [13]	Overall % of missing
**Description of missing data in patients that received home-treatment according to the original study definitions (n=3301)**											
n	297	513	739	604	520	164	72	62	130	200	
age (mean (SD))	54.53 (15.37)	53.48 (14.65)	56.68 (16.14)	51.97 (16.57)	56.33 (16.33)	55.94 (16.37)	60.01 (17.50)	62.48 (10.33)	60.42 (15.45)	44.20 (14.18)	0.0

Variables	Zondag et al.[3,4]	Exter et al. [5]	Roy et al.[6]	Kline et al.[7]	Barco et al. [8]	Kabrhel et al. [9]	Otero et al.[10]	Font et al. [11]	Vinson et al.[12]	Bledsoe et al. [13]	Overall % of missing
sex = female (%)	125 (42.1)	235 (45.8)	341 (46.1)	301 (49.8)	242 (46.5)	86 (52.4)	36 (50.0)	25 (40.3)	71 (54.6)	108 (54.0)	0.0
symp (%)											34.2
Symptomatic, n (%)	297 (100.0)	513 (100.0)	739 (100.0)	0 (0.0)	479 (92.1)	0 (0.0)	72 (100.0)	14 (22.6)	0 (0.0)	0 (0.0)	
Incidental, n (%)	0 (0.0)	0 (0.0)	0 (0.0)	0 (0.0)	33 (6.3)	0 (0.0)	0 (0.0)	48 (77.4)	0 (0.0)	0 (0.0)	
Missing, n (%)	0 (0.0)	0 (0.0)	0 (0.0)	604 (100.0)	8 (1.5)	164 (100.0)	0 (0.0)	0 (0.0)	130 (100.0)	200 (100.0)	
treat.med.bin (%)											6.4
DOAC, n (%)	0 (0.0)	0 (0.0)	587 (79.4)	604 (100.0)	520 (100.0)	57 (34.8)	0 (0.0)	0 (0.0)	0 (0.0)	172 (86.0)	
VKA/LWMH, n (%)	297 (100.0)	507 (98.8)	114 (15.4)	0 (0.0)	0 (0.0)	105 (64.0)	72 (100.0)	62 (100.0)	0 (0.0)	28 (14.0)	
missing, n (%)	0 (0.0)	6 (1.2)	38 (5.1)	0 (0.0)	0 (0.0)	2 (1.2)	0 (0.0)	0 (0.0)	130 (100.0)	0 (0.0)	
mal (%)											0.8
No, n (%)	269 (90.6)	474 (92.4)	676 (91.5)	580 (96.0)	470 (90.4)	98 (59.8)	70 (97.2)	0 (0.0)	113 (86.9)	198 (99.0)	
Yes, n (%)	28 (9.4)	33 (6.4)	51 (6.9)	24 (4.0)	40 (7.7)	66 (40.2)	2 (2.8)	62 (100.0)	17 (13.1)	2 (1.0)	
missing, n (%)	0 (0.0)	6 (1.2)	12 (1.6)	0 (0.0)	10 (1.9)	0 (0.0)	0 (0.0)	0 (0.0)	0 (0.0)	0 (0.0)	
prevvte (%)											8.7
No, n (%)	223 (75.1)	389 (75.8)	538 (72.8)	171 (28.3)	410 (78.8)	117 (71.3)	62 (86.1)	58 (93.5)	0 (0.0)	163 (81.5)	
Yes, n (%)	74 (24.9)	118 (23.0)	189 (25.6)	336 (55.6)	103 (19.8)	47 (28.7)	10 (13.9)	4 (6.5)	0 (0.0)	37 (18.5)	
missing, n (%)	0 (0.0)	6 (1.2)	12 (1.6)	97 (16.1)	7 (1.3)	0 (0.0)	0 (0.0)	0 (0.0)	130 (100.0)	0 (0.0)	
renal60 (%)											19.5
No, n (%)	91 (30.6)	456 (88.9)	567 (76.7)	435 (72.0)	465 (89.4)	142 (86.6)	68 (94.4)	57 (91.9)	0 (0.0)	185 (92.5)	
Yes, n (%)	3 (1.0)	21 (4.1)	60 (8.1)	53 (8.8)	55 (10.6)	15 (9.1)	3 (4.2)	2 (3.2)	2 (1.5)	6 (3.0)	
missing, n (%)	203 (68.4)	36 (7.0)	112 (15.2)	116 (19.2)	0 (0.0)	7 (4.3)	1 (1.4)	3 (4.8)	128 (98.5)	9 (4.5)	

Variables	Zondag et al.[3,4]	Exter et al. [5]	Roy et al.[6]	Kline et al.[7]	Barco et al. [8]	Kabrhel et al. [9]	Otero et al.[10]	Font et al. [11]	Vinson et al.[12]	Bledsoe et al. [13]	Overall % of missing
carpulm_calc (%)											38.3
No, n (%)	285 (96.0)	482 (94.0)	0 (0.0)	0 (0.0)	451 (86.7)	121 (73.8)	55 (76.4)	45 (72.6)	96 (73.8)	168 (84.0)	
Yes, n (%)	12 (4.0)	24 (4.7)	38 (5.1)	42 (7.0)	60 (11.5)	43 (26.2)	17 (23.6)	17 (27.4)	34 (26.2)	32 (16.0)	
missing, n (%)	0 (0.0)	7 (1.4)	701 (94.9)	562 (93.0)	9 (1.7)	0 (0.0)	0 (0.0)	0 (0.0)	0 (0.0)	0 (0.0)	
tropoabn (%)											56.7
No, n (%)	0 (0.0)	284 (55.4)	369 (49.9)	246 (40.7)	0 (0.0)	105 (64.0)	69 (95.8)	0 (0.0)	11 (8.5)	198 (99.0)	
Yes, n (%)	0 (0.0)	44 (8.6)	91 (12.3)	19 (3.1)	0 (0.0)	1 (0.6)	3 (4.2)	0 (0.0)	0 (0.0)	1 (0.5)	
missing, n (%)	297 (100.0)	185 (36.1)	279 (37.8)	339 (56.1)	520 (100.0)	58 (35.4)	0 (0.0)	62 (100.0)	119 (91.5)	1 (0.5)	
ntprobnpabn500 (%)											49.9
No, n (%)	0 (0.0)	450 (87.7)	296 (40.1)	122 (20.2)	338 (65.0)	79 (48.2)	0 (0.0)	0 (0.0)	40 (30.8)	190 (95.0)	
Yes, n (%)	0 (0.0)	23 (4.5)	34 (4.6)	27 (4.5)	33 (6.3)	5 (3.0)	0 (0.0)	0 (0.0)	16 (12.3)	7 (3.5)	
missing, n (%)	297 (100.0)	40 (7.8)	409 (55.3)	455 (75.3)	149 (28.7)	80 (48.8)	72 (100.0)	62 (100.0)	74 (56.9)	3 (1.5)	
rvlvratio9 (%)											54.4
No, n (%)	88 (29.6)	0 (0.0)	523 (70.8)	0 (0.0)	474 (91.2)	5 (3.0)	71 (98.6)	39 (62.9)	0 (0.0)	0 (0.0)	
Yes, n (%)	187 (63.0)	0 (0.0)	90 (12.2)	0 (0.0)	21 (4.0)	3 (1.8)	0 (0.0)	20 (32.3)	0 (0.0)	0 (0.0)	
missing, n (%)	22 (7.4)	513 (100.0)	126 (17.1)	604 (100.0)	25 (4.8)	156 (95.1)	1 (1.4)	3 (4.8)	130 (100.0)	200 (100.0)	
echo.rvdys (%)											74.1
No, n (%)	0 (0.0)	0 (0.0)	0 (0.0)	34 (5.6)	520 (100.0)	17 (10.4)	71 (98.6)	0 (0.0)	4 (3.1)	198 (99.0)	
Yes, n (%)	0 (0.0)	0 (0.0)	0 (0.0)	4 (0.7)	0 (0.0)	2 (1.2)	1 (1.4)	0 (0.0)	1 (0.8)	2 (1.0)	
missing, n (%)	297 (100.0)	513 (100.0)	739 (100.0)	566 (93.7)	0 (0.0)	145 (88.4)	0 (0.0)	62 (100.0)	125 (96.2)	0 (0.0)	
vtefup.14 (%)											1.8

Variables	Zondag et al.[3,4]	Exter et al. [5]	Roy et al.[6]	Kline et al.[7]	Barco et al. [8]	Kabrhel et al. [9]	Otero et al.[10]	Font et al. [11]	Vinson et al.[12]	Bledsoe et al. [13]	Overall % of missing
No, n (%)	293 (98.7)	512 (99.8)	734 (99.3)	598 (99.0)	512 (98.5)	123 (75.0)	68 (94.4)	59 (95.2)	129 (99.2)	200 (100.0)	
Yes, n (%)	3 (1.0)	0 (0.0)	1 (0.1)	5 (0.8)	1 (0.2)	0 (0.0)	2 (2.8)	1 (1.6)	1 (0.8)	0 (0.0)	
missing, n (%)	1 (0.3)	1 (0.2)	4 (0.5)	1 (0.2)	7 (1.3)	41 (25.0)	2 (2.8)	2 (3.2)	0 (0.0)	0 (0.0)	
mbfup.14 (%)											1.9
No, n (%)	296 (99.7)	511 (99.6)	733 (99.2)	603 (99.8)	512 (98.5)	118 (72.0)	68 (94.4)	58 (93.5)	129 (99.2)	200 (100.0)	
Yes, n (%)	1 (0.3)	1 (0.2)	3 (0.4)	0 (0.0)	1 (0.2)	0 (0.0)	1 (1.4)	1 (1.6)	1 (0.8)	0 (0.0)	
missing, n (%)	0 (0.0)	1 (0.2)	3 (0.4)	1 (0.2)	7 (1.3)	46 (28.0)	3 (4.2)	3 (4.8)	0 (0.0)	0 (0.0)	
nonmbfup.14 (%)											38.9
No, n (%)	0 (0.0)	0 (0.0)	728 (98.5)	596 (98.7)	500 (96.2)	116 (70.7)	68 (94.4)	0 (0.0)	0 (0.0)	0 (0.0)	
Yes, n (%)	0 (0.0)	0 (0.0)	7 (0.9)	6 (1.0)	11 (2.1)	6 (3.7)	1 (1.4)	0 (0.0)	0 (0.0)	0 (0.0)	
missing, n (%)	297 (100.0)	513 (100.0)	4 (0.5)	2 (0.3)	9 (1.7)	42 (25.6)	3 (4.2)	62 (100.0)	130 (100.0)	200 (100.0)	
deathfup.14 (%)											1.6
No, n (%)	296 (99.7)	512 (99.8)	735 (99.5)	603 (99.8)	513 (98.7)	123 (75.0)	68 (94.4)	60 (96.8)	130 (100.0)	200 (100.0)	
Yes, n (%)	1 (0.3)	1 (0.2)	1 (0.1)	0 (0.0)	0 (0.0)	1 (0.6)	2 (2.8)	2 (3.2)	0 (0.0)	0 (0.0)	
missing, n (%)	0 (0.0)	0 (0.0)	3 (0.4)	1 (0.2)	7 (1.3)	40 (24.4)	2 (2.8)	0 (0.0)	0 (0.0)	0 (0.0)	
combfup.14 (%)											1.8
No, n (%)	293 (98.7)	511 (99.6)	732 (99.1)	598 (99.0)	511 (98.3)	118 (72.0)	68 (94.4)	57 (91.9)	129 (99.2)	200 (100.0)	
Yes, n (%)	4 (1.3)	2 (0.4)	4 (0.5)	5 (0.8)	2 (0.4)	1 (0.6)	3 (4.2)	3 (4.8)	1 (0.8)	0 (0.0)	
missing, n (%)	0 (0.0)	0 (0.0)	3 (0.4)	1 (0.2)	7 (1.3)	45 (27.4)	1 (1.4)	2 (3.2)	0 (0.0)	0 (0.0)	
immosurg (%)											14.3
No, n (%)	269 (90.6)	437 (85.2)	633 (85.7)	546 (90.4)	435 (83.7)	136 (82.9)	59 (81.9)	0 (0.0)	0 (0.0)	0 (0.0)	

Variables	Zondag et al.[3,4]	Exter et al. [5]	Roy et al.[6]	Kline et al.[7]	Barco et al. [8]	Kabrhel et al. [9]	Otero et al.[10]	Font et al. [11]	Vinson et al.[12]	Bledsoe et al. [13]	Overall % of missing
Yes, n (%)	27 (9.1)	68 (13.3)	71 (9.6)	54 (8.9)	77 (14.8)	28 (17.1)	13 (18.1)	7 (11.3)	0 (0.0)	0 (0.0)	
missing, n (%)	1 (0.3)	8 (1.6)	35 (4.7)	4 (0.7)	8 (1.5)	0 (0.0)	0 (0.0)	55 (88.7)	130 (100.0)	200 (100.0)	
estrogen (%)											9.4
No, n (%)	249 (83.8)	416 (81.1)	663 (89.7)	563 (93.2)	429 (82.5)	155 (94.5)	68 (94.4)	54 (87.1)	59 (45.4)	0 (0.0)	
Yes, n (%)	47 (15.8)	91 (17.7)	64 (8.7)	40 (6.6)	86 (16.5)	9 (5.5)	4 (5.6)	8 (12.9)	0 (0.0)	0 (0.0)	
missing, n (%)	1 (0.3)	6 (1.2)	12 (1.6)	1 (0.2)	5 (1.0)	0 (0.0)	0 (0.0)	0 (0.0)	71 (54.6)	200 (100.0)	
tropongml (mean (SD))		0.01 (0.03)	1.09 (5.65)	0.50 (2.23)	0.14 (1.24)	0.01 (0.01)			0.02 (0.00)	0.01 (0.01)	44.8
hr110 (%)											6.6
No, n (%)	236 (79.5)	478 (93.2)	661 (89.4)	536 (88.7)	512 (98.5)	0 (0.0)	63 (87.5)	62 (100.0)	113 (86.9)	182 (91.0)	
Yes, n (%)	24 (8.1)	35 (6.8)	66 (8.9)	62 (10.3)	7 (1.3)	0 (0.0)	9 (12.5)	0 (0.0)	17 (13.1)	18 (9.0)	
missing, n (%)	37 (12.5)	0 (0.0)	12 (1.6)	6 (1.0)	1 (0.2)	164 (100.0)	0 (0.0)	0 (0.0)	0 (0.0)	0 (0.0)	
bpsyst100 (%)											6.6
No, n (%)	260 (87.5)	513 (100.0)	722 (97.7)	588 (97.4)	518 (99.6)	0 (0.0)	71 (98.6)	62 (100.0)	122 (93.8)	195 (97.5)	
Yes, n (%)	1 (0.3)	0 (0.0)	4 (0.5)	10 (1.7)	0 (0.0)	0 (0.0)	1 (1.4)	0 (0.0)	8 (6.2)	5 (2.5)	
missing, n (%)	36 (12.1)	0 (0.0)	13 (1.8)	6 (1.0)	2 (0.4)	164 (100.0)	0 (0.0)	0 (0.0)	0 (0.0)	0 (0.0)	
resp30 (%)											58.3
No, n (%)	0 (0.0)	0 (0.0)	0 (0.0)	591 (97.8)	424 (81.5)	0 (0.0)	0 (0.0)	0 (0.0)	126 (96.9)	198 (99.0)	
Yes, n (%)	0 (0.0)	0 (0.0)	0 (0.0)	5 (0.8)	6 (1.2)	0 (0.0)	0 (0.0)	0 (0.0)	4 (3.1)	2 (1.0)	
missing, n (%)	297 (100.0)	513 (100.0)	739 (100.0)	8 (1.3)	90 (17.3)	164 (100.0)	72 (100.0)	62 (100.0)	0 (0.0)	0 (0.0)	
sat90 (%)											7.7
No, n (%)	234 (78.8)	509 (99.2)	700 (94.7)	595 (98.5)	494 (95.0)	0 (0.0)	66 (91.7)	62 (100.0)	122 (93.8)	200 (100.0)	

Variables	Zondag et al.[3,4]	Exter et al. [5]	Roy et al.[6]	Kline et al.[7]	Barco et al. [8]	Kabrhel et al. [9]	Otero et al.[10]	Font et al. [11]	Vinson et al.[12]	Bledsoe et al. [13]	Overall % of missing
Yes, n (%)	17 (5.7)	4 (0.8)	24 (3.2)	2 (0.3)	1 (0.2)	0 (0.0)	6 (8.3)	0 (0.0)	8 (6.2)	0 (0.0)	
missing, n (%)	46 (15.5)	0 (0.0)	15 (2.0)	7 (1.2)	25 (4.8)	164 (100.0)	0 (0.0)	0 (0.0)	0 (0.0)	0 (0.0)	
creat (mean (SD))	107.56 (31.42)	113.46 (37.64)	102.97 (36.57)	116.24 (52.36)	81.57 (18.61)	90.59 (20.05)	97.00			89.87 (18.55)	23.5
location (%)											73.2
Central	31 (10.4)	0 (0.0)	0 (0.0)	22 (3.6)	0 (0.0)	6 (3.7)	0 (0.0)	11 (17.7)	18 (13.8)	0 (0.0)	
Lobar	0 (0.0)	0 (0.0)	0 (0.0)	94 (15.6)	0 (0.0)	30 (18.3)	0 (0.0)	26 (41.9)	28 (21.5)	0 (0.0)	
Segmental	89 (30.0)	0 (0.0)	0 (0.0)	126 (20.9)	0 (0.0)	77 (47.0)	0 (0.0)	15 (24.2)	42 (32.3)	87 (43.5)	
Subsegmental	22 (7.4)	0 (0.0)	0 (0.0)	41 (6.8)	0 (0.0)	36 (22.0)	0 (0.0)	5 (8.1)	19 (14.6)	34 (17.0)	
missing, n (%)	155 (52.2)	513 (100.0)	739 (100.0)	321 (53.1)	520 (100.0)	15 (9.1)	72 (100.0)	5 (8.1)	23 (17.7)	79 (39.5)	

Table S2: risk of bias assessment using the adapted NOS (Newcastle-Ottawa Scale)

Study	Stars of selection	Stars for outcome	Total number of stars	Overall risk of bias assessment
Barco et al.[8]	★★★	★★	5	Low risk of bias
Bledsoe et al.[13]	★★★	★★★	6	Low risk of bias
Exter et al.[5] Hestia + normal NTproBNP group	★★★	★★★	6	Low risk of bias
Exter et al.[5] Hestia group	★★★	★★★	6	Low risk of bias
Font et al.[11]	★★	★★	4	Moderate risk of bias
Kabrhel et al. [9]	★★	★★★	5	Low risk of bias
Kline et al.[7]	★★★	★★★	6	Low risk of bias
Otero et al.[10]	★★★	★★★	6	Low risk of bias
Roy et al.[6] Hestia group	★★	★★★	5	Low risk of bias
Roy et al.[6] sPESI group	★★	★★★	5	Low risk of bias
Vinson et al.[12]	★★★	★★★	6	Low risk of bias
Zondag et al. [3,4]	★★★	★★★	6	Low risk of bias

All studies, including each arm of a randomized trial, as an independent observational cohort, was assessed on risk of bias by using a modified version of NOS for observational studies.

The NOS was modified by removing item 2 from selection ("Selection of the non-exposed cohort") and question 1 from comparability ("Comparability of cohorts on the basis of the design or analysis") as each study included was an independent observational cohort and therefore there we non non-exposed cohorts and no comparison made between two cohorts within each study.

Table S3: overall and subgroup analysis at 14- and 30-days for recurrent VTE incidence

This table depicts the recurrent VTE outcomes of all patients that received home-treatment according to our IPDMA definition of home-treatment (discharge within 24 hours)

		Recurrent VTE at 14 days							Recurrent VTE at 30 days						
**subgroup variable**	**subgroup level**	**Events (n)**	**Patients (n)**	**%**	**(95%CI)**	**RR**	**(95%PI)**	**I2**	**Events (n)**	**Patients (n)**	**%**	**(95%CI)**	**RR**	**(95%PI)**	**I2**
overall		9	2661	0.34	(0.12 to 0.56)				15	2654	0.57	(0.28 to 0.86)			
Sex	Male*	7	1397	0.50	(0.13 to 0.87)	0.74	(0.31 to 1.81)	0	10	1394	0.72	(0.28 to 1.16)	0.78	(0.36 to 1.71)	0
	Female	2	1264	0.16	(0 to 0.38)				5	1260	0.40	(0.05 to 0.75)			
Cancer#	Yes	0	218	0.00	(0 to 0)	0.71	(0.41 to 1.22)	0	0	212	0.00	(0 to 0)	0.61	(0.31 to 1.17)	0
	No*	9	2443	0.37	(0.13 to 0.61)				15	2442	0.61	(0.3 to 0.92)			
Previous VTE	Yes	6	830	0.69	(0.13 to 1.25)	1.41	(0.68 to 2.92)	0	10	829	1.21	(0.46 to 1.96)	2.22	(1 to 4.95)	0
	No*	3	1715	0.19	(0 to 0.4)				5	1710	0.29	(0.04 to 0.54)			
Decreased kidney function^	Yes	1	203	0.25	(0 to 0.94)	0.63	(0.32 to 1.24)	0	1	203	0.31	(0 to 1.07)	0.51	(0.25 to 1.06)	0
	No*	8	2458	0.35	(0.12 to 0.58)				14	2451	0.59	(0.29 to 0.89)			
Preexisting cardio-pulmonary disease¥	Yes	4	479	0.92	(0.07 to 1.77)	2.93	(1.29 to 6.64)	0	5	478	1.11	(0.17 to 2.05)	2.19	(1.06 to 4.55)	0
	No*	5	2182	0.21	(0.02 to 0.4)				10	2176	0.45	(0.17 to 0.73)			
Treatment	DOAC*	6	1534	0.39	(0.08 to 0.7)	0.78	(0.26 to 2.38)	0	10	1533	0.65	(0.25 to 1.05)	0.68	(0.12 to 3.85)	0
	LMWH or VKA	3	1011	0.30	(0 to 0.64)				5	1006	0.50	(0.07 to 0.93)			
Abnormal troponinΩ	Yes	1	248	0.39	(0 to 1.17)	0.89	(0.74 to 1.08)	0	2	247	0.81	(0 to 1.93)	2.09	(0.63 to 6.9)	0
	No*	5	1949	0.26	(0.03 to 0.49)				9	1944	0.46	(0.16 to 0.76)			
Abnormal (NT-pro)BNP±	Yes	1	210	0.55	(0 to 1.55)	0.90	(0.74 to 1.08)	0	2	209	0.75	(0 to 1.92)	0.83	(0.53 to 1.3)	0
	No*	5	2156	0.23	(0.03 to 0.43)				9	2151	0.44	(0.16 to 0.72)			
Signs of RV overload§	Yes	4	327	1.22	(0.03 to 2.41)	4.82	(1.26 to 18.38)	0	5	325	1.54	(0.2 to 2.88)	3.04	(0.1 to 88.38)	17.1
	No*	0	911	0.00	(0 to 0)				1	907	0.11	(0 to 0.33)			
RV dysfunction on TTE	Yes	0	30	1.60	(0 to 6.1)	0.80	(0.45 to 1.43)	0	1	30	1.84	(0 to 6.67)	0.71	(0.3 to 1.69)	0
	No*	5	1147	0.39	(0.03 to 0.75)				7	1142	0.65	(0.18 to 1.12)			
Symptoms	Incidental *	0	15	0.00	(0 to 0)	1.00	(0 to 1005.22)	0	0	15	0.00	(0 to 0)	1.00	(0 to 1005.22)	0
	Symptomatic	4	1638	0.24	(0 to 0.48)				7	1636	0.43	(0.11 to 0.75)			
Age	18-40*	3	582	0.52	(0 to 1.1)				4	581	0.69	(0.02 to 1.36)			
	41-60	4	1084	0.37	(0.01 to 0.73)	0.89	(0.53 to 1.5)	0	6	1084	0.55	(0.11 to 0.99)	0.93	(0.42 to 2.06)	0
	61-80	2	895	0.22	(0 to 0.53)	0.79	(0.45 to 1.39)	0	5	889	0.56	(0.07 to 1.05)	0.91	(0.67 to 1.25)	0
	>81	0	99	0.00	(0 to 0)	0.77	(0.52 to 1.15)	0	0	99	0.00	(0 to 0)	0.72	(0.45 to 1.17)	0

I^2 was 0% and 0 for all proportion analysis RR presents the ratio of the risk for an event for the exposure group to the risk for the non-exposure/reference group; non-exposure/reference group is marked with an asterisk # (1) Current diagnosis of cancer, (2) receiving treatment for cancer or (3) not receiving treatment for cancer and not in complete response ; ^Estimated Glomerular Filtration Rate < 60 ml/min; ¥Preexisting pulmonary disease was defined as a history of chronic obstructive pulmonary disease, asthma, or lung fibrosis, a preexisting cardiovascular disease, defined as any of coronary artery disease, heart failure, congenital heart disease, cardiomyopathy or rheumatic heart disease; ΩAbnormal troponin was defined as a troponin level >99th percentile according to local technique; ± NT-proBNP > 500 ng/L or BNP level >100 ng/L ; §Right ventricle/ left ventricle ratio >0.9 on computed tomography pulmonary angiogram or echocardiogram; Abbreviations: CI, confidence interval; DOAC direct oral anticoagulant; LMWH low molecular weight heparin; MB, major bleeding; NA not applicable; PI prediction interval; RR relative risk; RV right ventricle; TTE trans thoracic echocardiography; VKA vitamin K antagonist; VTE, venous thromboembolism.

Table S4: overall and subgroup analysis at 14- and 30-days for major bleeding

This table depicts the major bleeding outcomes of all patients that received home-treatment according to our IPDMA definition of home-treatment (discharge within 24 hours)

		Major bleeding at 14 days							Major bleeding at 30 days						
**subgroup variable**	**subgroup level**	**Events (n)**	**Patients (n)**	**%**	**(95%CI)**	**RR**	**(95%PI)**	**I2**	**Events (n)**	**Patients (n)**	**%**	**(95%CI)**	**RR**	**(95%PI)**	**I2**
overall		5	2659	0.19	(0.03 to 0.35)				12	2648	0.45	(0.19 to 0.71)			
Sex	Male*	2	1396	0.14	(0 to 0.34)	1.28	(0.73 to 2.26)	0	3	1392	0.22	(0 to 0.46)	1.91	(1.11 to 3.3)	0
	Female	3	1263	0.24	(0 to 0.51)				9	1256	0.72	(0.25 to 1.19)			
Cancer[#]	Yes	1	217	0.46	(0 to 1.36)	2.14	(0.98 to 4.68)	0	2	209	0.95	(0 to 2.27)	2.89	(1.22 to 6.87)	0
	No*	4	2442	0.16	(0 to 0.32)				10	2439	0.41	(0.16 to 0.66)			
Previous VTE	Yes	0	829	0.00	(0 to 0)	0.59	(0.32 to 1.09)	0	1	826	0.12	(0 to 0.36)	0.50	(0.24 to 1.04)	0
	No*	5	1714	0.29	(0.03 to 0.55)				11	1707	0.64	(0.26 to 1.02)			
Decreased kidney function[^]	Yes	0	203	0.00	(0 to 0)	0.69	(0.41 to 1.15)	0	0	202	0.01	(0 to 0.12)	0.46	(0.23 to 0.92)	0
	No*	5	2456	0.20	(0.02 to 0.38)				12	2446	0.49	(0.21 to 0.77)			
Preexisting cardio-pulmonary disease[¥]	Yes	1	478	0.17	(0 to 0.54)	0.92	(0.78 to 1.09)	0	2	476	0.37	(0 to 0.92)	0.76	(0.58 to 1.01)	0
	No*	4	2181	0.19	(0.01 to 0.37)				10	2172	0.47	(0.18 to 0.76)			
Treatment	DOAC*	2	1532	0.13	(0 to 0.31)	1.65	(0.63 to 4.33)	0	6	1530	0.39	(0.08 to 0.7)	1.89	(0.75 to 4.78)	0
	LMWH or VKA	3	1011	0.30	(0 to 0.64)				6	1003	0.60	(0.12 to 1.08)			
Abnormal troponin[Ω]	Yes	2	249	0.84	(0 to 1.98)	3.56	(1.26 to 10.07)	0	3	247	1.29	(0 to 2.7)	2.52	(1.37 to 4.62)	0
	No*	2	1945	0.10	(0 to 0.24)				7	1937	0.35	(0.09 to 0.61)			
Abnormal (NT-pro)BNP[±]	Yes	2	210	1.05	(0 to 2.43)	4.88	(1.58 to 15.06)	0	3	207	1.60	(0 to 3.31)	4.19	(1.82 to 9.67)	0
	No*	2	2153	0.08	(0 to 0.2)				7	2146	0.31	(0.07 to 0.55)			
Signs of RV overload[§]	Yes	1	326	0.46	(0 to 1.19)	1.17	(0.03 to 39.93)	15.4	3	324	0.79	(0 to 1.76)	1.09	(0.44 to 2.73)	0
	No*	3	910	0.28	(0 to 0.62)				5	904	0.60	(0.1 to 1.1)			
RV dysfunction on TTE	Yes	0	29	0.00	(0 to 0)	NA	NA		0	28	0.19	(0 to 1.78)	NA	NA	
	No*	0	1144	0.00	(0 to 0)				2	1137	0.17	(0 to 0.41)			
Symptoms	Incidental *	0	15	0.00	(0 to 0)	1.00	(0 to 1005.22)	0	0	15	0.00	(0 to 0)	1.00	(0 to 1005.22)	0
	Symptomatic	5	1640	0.30	(0.03 to 0.57)				10	1637	0.61	(0.23 to 0.99)			
Age	18-40*	0	580	0.00	(0 to 0)				3	579	0.52	(0 to 1.1)			
	41-60	3	1085	0.28	(0 to 0.59)	1.40	(0.87 to 2.26)	0	5	1084	0.46	(0.06 to 0.86)	0.89	(0.51 to 1.57)	0
	61-80	2	894	0.22	(0 to 0.53)	1.32	(0.9 to 1.93)	0	4	885	0.45	(0.01 to 0.89)	0.85	(0.54 to 1.32)	0
	>81	0	99	0.00	(0 to 0)	NA	NA		0	99	0.00	(0 to 0)	0.74	(0.52 to 1.04)	0

I^2 was 0% and 0 for all proportion analysis * RR presents the ratio of the risk for an event for the exposure group to the risk for the non-exposure/reference group; non-exposure/reference group is marked with an asterisk # (1) Current diagnosis of cancer, (2) receiving treatment for cancer or (3) not receiving treatment for cancer and not in complete response ; ^Estimated Glomerular Filtration Rate < 60 ml/min; ¥Preexisting pulmonary disease was defined as a history of chronic obstructive pulmonary disease, asthma, or lung fibrosis, a preexisting cardiovascular disease, defined as any of coronary artery disease, heart failure, congenital heart disease, cardiomyopathy or rheumatic heart disease; ΩAbnormal troponin was defined as a troponin level >99th percentile according to local technique; ± NT-proBNP > 500 ng/L or BNP level >100 ng/L ; §Right ventricle/ left ventricle ratio >0.9 on computed tomography pulmonary angiogram or echocardiogram; Abbreviations: CI, confidence interval; DOAC direct oral anticoagulant; LMWH low molecular weight heparin; MB, major bleeding; NA not applicable; PI prediction interval; RR relative risk; RV right ventricle; TTE trans thoracic echocardiography; VKA vitamin K antagonist; VTE, venous thromboembolism.

Sensitivity analysis: according to the definition of home-treatment of the original studies

Table S5: characteristics of patients that received home treatment according to the original study definitions

Study, year (reference)	Overall	Zondag et al.[3,4]	Exter et al. [5]	Roy et al.[6]	Kline et al.[7]	Barco et al. [8]	Kabrhel et al. [9]	Otero et al.[10]	Font et al. [11]	Vinson et al.[12]	Bledsoe et al. [13]
Patients, n	3301	297	513	739	604	520	164	72	62	130	200
Mean age (SD)	54.61 (16.15)	54.53 (15.37)	53.48 (14.65)	56.68 (16.14)	51.97 (16.57)	56.33 (16.33)	55.94 (16.37)	60.01 (17.50)	62.48 (10.33)	60.42 (15.45)	44.20 (14.18)
Female, n (%)	1570 (48%)	125 (42%)	235 (46%)	341 (46%)	301 (50%)	242 (47%)	86 (52%)	36 (50%)	25 (40%)	71 (55%)	108 (54%)
Eligibility criteria applied, n (%)											
Negative Hestia (like) rule	1713 (52%)	297 (100%)	513 (100%)	378 (51%)	463 (77%)	0 (0%)	0 (0%)	0 (0%)	62 (100%)	0 (0%)	0 (0%)
sPESI 0 or PESI II/II	502 (16%)	0 (0%)	0 (0%)	361 (49%)	141 (23%)	0 (0%)	0 (0%)	0 (0%)	0 (0%)	0 (0%)	0 (0%)
sPESI 0 or PESI II/II and absence of RVD	330 (10)	0 (0%)	0 (0%)	0 (0%)	0 (0%)	0 (0%)	0 (0%)	0 (0%)	0 (0%)	130 (100%)	200 (100%)
Negative Hestia (like) rule and absence of RVD	520 (16%)	0 (0%)	0 (0%)	0 (0%)	0 (0%)	520 (100%)	0 (0%)	72 (100%)	0 (0%)	0 (0%)	0 (0%)
Other tool	236 (7.1%)	0 (0%)	0 (0%)	0 (0%)	0 (0%)	0 (0%)	164 (100%)	0 (0%)	0 (0%)	0 (0%)	0 (0%)
Treatment with a DOAC, n (%)	1969 (62%)	0 (0%)	3 (1%)	612 (83%)	604 (100%)	520 (100%)	58 (35%)	0 (0%)	0 (0%)	*	172 (86%)
Risk factors, n (%)											
Recent immobilization or surgery	382 (13%)	27 (9%)	69 (13%)	74 (10%)	54 (9%)	78 (15%)	28 (17%)	13 (18%)	39 (63%)	*	*
Estrogen use	360 (12%)	47 (16%)	91 (18%)	65 (9%)	41 (7%)	87 (17%)	9 (5%)	4 (6%)	8 (13%)	9 (7%)	*
Symptomatic PE, n (%)	2121 (96%)	297 (100%)	513 (100%)	739 (100%)	*	486 (93%)	*	72 (100%)	14 (23%)	*	*
Vital signs at presentation, n (%)											
Heart rate ≥110/min	242 (8%)	27 (9%)	35 (7%)	67 (9%)	62 (10%)	7 (1%)	*	9 (12%)	0 (0%)	17 (13%)	18 (9%)
Respiratory rate of ≥ 30/min	18 (1%)	*	*	*	5 (1%)	7 (1%)	*	*	*	4 (3%)	2 (1%)
Oxygen saturation <90% or need for oxygen	65 (2%)	19 (6%)	4 (1%)	24 (3%)	2 (0%)	1 (0%)	*	6 (8%)	0 (0%)	8 (6%)	0 (0%)
Comorbidities, n (%)											
Cancer #	326 (10%)	28 (9%)	34 (7%)	52 (7%)	24 (4%)	40 (8%)	66 (40%)	2 (3%)	62 (100%)	17 (13%)	2 (1%)
Previous VTE	965 (30%)	74 (25%)	120 (23%)	193 (26%)	375 (62%)	105 (20%)	47 (29%)	10 (14%)	4 (6%)	*	37 (18%)
Decreased kidney function^	271 (8%)	12 (4%)	22 (4%)	74 (10%)	67 (11%)	55 (11%)	15 (9%)	3 (4%)	2 (3%)	15 (12%)	6 (3%)
Cardiopulmonary comorbidity¥	603 (18%)	12 (4%)	25 (5%)	175 (24%)	186 (31%)	62 (12%)	43 (26%)	17 (24%)	17 (27%)	34 (26%)	32 (16%)
Laboratory/imaging results, n (%)											
Abnormal troponinΩ	269 (11%)	*	67 (13%)	129 (17%)	53 (9%)	*	5 (3%)	3 (4%)	*	11 (8%)	1 (0%)
Abnormal (NT-pro)BNP±	290 (10%)	*	27 (5%)	86 (12%)	80 (13%)	50 (10%)	13 (8%)	*	*	27 (21%)	7 (4%)
RV overload§	376 (20%)	201 (68%)	*	112 (15%)	*	23 (4%)	19 (12%)	0 (0%)	21 (34%)	*	*
RVD on echocardiography	34 (2%)	*	*	*	20 (3%)	0 (0%)	8 (5%)	1 (1%)	*	3 (2%)	2 (1%)

* Variable systematically missing within a study. # (1) Current diagnosis of cancer, (2) receiving treatment for cancer or (3) not receiving treatment for cancer and not in complete response; ^Estimated Glomerular Filtration Rate < 60 ml/min; ¥Preexisting pulmonary disease was defined as a history of chronic obstructive pulmonary disease, asthma, or lung fibrosis, a preexisting cardiovascular disease, defined as any of coronary artery disease, heart failure, congenital heart disease, cardiomyopathy or rheumatic heart disease ; ΩAbnormal troponin was defined as a troponin level >99th percentile according to local technique; ± NT-proBNP > 500 ng/L or BNP level >100 ng/L ; §Right ventricle/ left ventricle ratio >0.9 on computed tomography pulmonary angiogram or echocardiogram. Abbreviation: DOAC direct oral anticoagulant; PE pulmonary embolism; RVD right ventricle dysfunction SD standard deviation; VTE venous thromboembolism; RV/LV right ventricle/left ventricle

Table S6: overall and subgroup analysis at 14- and 30-days for mortality: sensitivity analysis according to the definition of home-treatment of the original studies

This table depicts the mortality outcomes of all patients that received home-treatment according to the definition of home-treatment of the original studies (discharge within 120 hours)

		mortality at 14 days							mortality at 30 days						
**subgroup variable**	**subgroup level**	**Events (n)**	**Patients (n)**	**%**	**(95%CI)**	**RR**	**(95%PI)**	**I2**	**Events (n)**	**Patients (n)**	**%**	**(95%CI)**	**RR**	**(95%PI)**	**I2**
overall		8	3,248	0.25	(0.08 to 0.42)				13	3,239	0.40	(0.18 to 0.62)			
Sex	Female	5	1,544	0.32	(0.04 to 0.6)	1.40	(0.65 to 3.01)	0	9	1,539	0.58	(0.2 to 0.96)	1.65	(0.84 to 3.25)	0
	Male*	3	1,704	0.18	(0 to 0.38)				4	1,700	0.24	(0.01 to 0.47)			
Cancer[#]	Yes	4	317	1.26	(0.03 to 2.49)	3.61	(1.25 to 10.43)	0	7	312	2.24	(0.6 to 3.88)	5.52	(3.2 to 9.53)	0
	No*	4	2,931	0.14	(0.01 to 0.27)				6	2,927	0.20	(0.04 to 0.36)			
Previous VTE	Yes	2	946	0.21	(0 to 0.5)	1.54	(0.62 to 3.85)	0	4	943	0.42	(0.01 to 0.83)	1.84	(0.79 to 4.26)	0
	No*	6	2,172	0.28	(0.06 to 0.5)				8	2,166	0.37	(0.11 to 0.63)			
Decreased kidney function[^]	Yes	0	264	0.00	(0 to 0)	0.66	(0.48 to 0.91)	0	0	264	0.14	(0 to 0.59)	0.59	(0.37 to 0.93)	0
	No*	8	2,984	0.27	(0.08 to 0.46)				13	2,975	0.42	(0.19 to 0.65)			
Preexisting cardio-pulmonary disease[¥]	Yes	2	590	0.40	(0 to 0.91)	1.87	(0.66 to 5.26)	0	3	588	0.45	(0 to 0.99)	1.60	(0.61 to 4.24)	0
	No*	6	2,658	0.21	(0.03 to 0.39)				10	2,651	0.39	(0.15 to 0.63)			
Treatment	DOAC*	0	1,938	0.00	(0 to 0)	3.86	(0.45 to 33.36)	0	1	1,934	0.05	(0 to 0.15)	2.90	(0.91 to 9.28)	0
	LMWH or VKA	8	1,180	0.68	(0.21 to 1.15)				11	1,175	0.94	(0.39 to 1.49)			
Abnormal troponin[Ω]	Yes	0	267	0.13	(0 to 0.56)	0.77	(0.49 to 1.21)	0	1	267	0.55	(0 to 1.44)	1.92	(0.58 to 6.31)	0
	No*	5	2,109	0.22	(0.02 to 0.42)				8	2,104	0.36	(0.1 to 0.62)			
Abnormal (NT-pro)BNP[±]	Yes	0	260	0.08	(0 to 0.43)	0.85	(0.64 to 1.14)	0	1	260	0.32	(0 to 1.01)	0.80	(0.47 to 1.34)	0
	No*	3	2,559	0.11	(0 to 0.24)				6	2,552	0.24	(0.05 to 0.43)			
Signs of RV overload[§]	Yes	2	371	0.47	(0 to 1.17)	0.89	(0.27 to 2.88)	0	3	371	0.82	(0 to 1.74)	1.00	(0.53 to 1.88)	0
	No*	5	1,431	0.37	(0.06 to 0.68)				7	1,422	0.49	(0.13 to 0.85)			
RV dysfunction on TTE	Yes	0	33	0.20	(0 to 1.72)	0.73	(0.43 to 1.23)	0	0	33	0.36	(0 to 2.41)	0.58	(0.33 to 1.02)	0
	No*	3	1,607	0.18	(0 to 0.39)				5	1,598	0.31	(0.04 to 0.58)			
Symptoms	Symptomatic*	5	2,110	0.24	(0.03 to 0.45)	2.69	(0 to 2314.71)	0	8	2,104	0.38	(0.12 to 0.64)	2.69	(0 to 2342.59)	0
	Incidental	2	81	2.48	(0 to 5.87)				2	81	2.48	(0 to 5.87)			
Age	18-40*	1	670	0.15	(0 to 0.44)				2	670	0.30	(0 to 0.71)			
	41-60	4	1,288	0.31	(0.01 to 0.61)	0.92	(0.51 to 1.67)	0	4	1,286	0.31	(0.01 to 0.61)	0.73	(0.37 to 1.44)	0
	61-80	2	1,159	0.17	(0 to 0.41)	0.84	(0.57 to 1.25)	0	6	1,154	0.52	(0.11 to 0.93)	0.92	(0.45 to 1.89)	0
	>81	1	130	0.77	(0 to 2.27)	1.24	(0.62 to 2.47)	0	1	128	0.78	(0 to 2.31)	1.11	(0.7 to 1.77)	0

I^2 was 0% and 0 for all proportion analysis * RR presents the ratio of the risk for an event for the exposure group to the risk for the non-exposure/reference group; non-exposure/reference group is marked with an asterisk # (1) Current diagnosis of cancer, (2) receiving treatment for cancer or (3) not receiving treatment for cancer and not in complete response ; ^Estimated Glomerular Filtration Rate < 60 ml/min; ¥Preexisting pulmonary disease was defined as a history of chronic obstructive pulmonary disease, asthma, or lung fibrosis, a preexisting cardiovascular disease, defined as any of coronary artery disease, heart failure, congenital heart disease, cardiomyopathy or rheumatic heart disease; ΩAbnormal troponin was defined as a troponin level >99th percentile according to local technique; ± NT-proBNP > 500 ng/L or BNP level >100 ng/L ; §Right ventricle/ left ventricle ratio >0.9 on computed tomography pulmonary angiogram or echocardiogram; Abbreviations: CI, confidence interval; DOAC direct oral anticoagulant; LMWH low molecular weight heparin; MB, major bleeding; NA not applicable; PI prediction interval; RR relative risk; RV right ventricle; TTE trans thoracic echocardiography; VKA vitamin K antagonist; VTE, venous thromboembolism.

Table S7: overall and subgroup analysis at 14- and 30-days for all adverse events (i.e. combined endpoint of recurrent VTE, MB or mortality): sensitivity analysis according to the definition of home-treatment of the original studies

This table depicts the adverse events outcomes of all patients that received home-treatment according to the definition of home-treatment of the original studies (discharge within 120 hours)

		adverse events at 14 days							adverse events at 30 days						
**subgroup variable**	**subgroup level**	**Events (n)**	**Patients (n)**	**%**	**(95%CI)**	**RR**	**(95%PI)**	**I2**	**Events (n)**	**Patients (n)**	**%**	**(95%CI)**	**RR**	**(95%PI)**	**I2**
overall		25	3,242	0.77	(0.47 to 1.07)				44	3,231	1.36	(0.96 to 1.76)			
Sex	Female	11	1,542	0.71	(0.29 to 1.13)	1.16	(0.57 to 2.35)	0	24	1,536	1.56	(0.94 to 2.18)	1.37	(0.65 to 2.86)	3.09
	Male*	14	1,700	0.82	(0.39 to 1.25)				20	1,695	1.18	(0.67 to 1.69)			
Cancer[#]	Yes	5	313	1.60	(0.21 to 2.99)	1.85	(0.76 to 4.52)	0	11	308	3.57	(1.5 to 5.64)	3.63	(1.89 to 6.99)	0
	No*	20	2,929	0.68	(0.38 to 0.98)				33	2,923	1.13	(0.75 to 1.51)			
Previous VTE	Yes	10	945	1.03	(0.39 to 1.67)	2.14	(0.97 to 4.72)	0	16	941	1.71	(0.88 to 2.54)	1.65	(0.91 to 3.01)	0
	No*	14	2,167	0.66	(0.32 to 1)				26	2,160	1.20	(0.74 to 1.66)			
Decreased kidney function[^]	Yes	1	264	0.40	(0 to 1.17)	0.42	(0.22 to 0.78)	0	2	262	0.60	(0 to 1.53)	0.34	(0.15 to 0.75)	0
	No*	24	2,978	0.80	(0.48 to 1.12)				42	2,969	1.43	(1 to 1.86)			
Preexisting cardio-pulmonary disease[¥]	Yes	9	588	1.57	(0.57 to 2.57)	2.84	(1.3 to 6.19)	0	12	585	2.11	(0.95 to 3.27)	1.77	(1.01 to 3.11)	0
	No*	16	2,654	0.59	(0.3 to 0.88)				32	2,646	1.20	(0.79 to 1.61)			
Treatment	DOAC*	10	1,936	0.52	(0.2 to 0.84)	1.57	(0.75 to 3.28)	0	19	1,931	0.98	(0.54 to 1.42)	1.52	(0.49 to 4.71)	0
	LMWH or VKA	14	1,176	1.19	(0.57 to 1.81)				23	1,170	1.97	(1.17 to 2.77)			
Abnormal troponin[Ω]	Yes	4	267	1.34	(0 to 2.72)	2.36	(1.19 to 4.69)	0	7	266	2.56	(0.66 to 4.46)	2.76	(1.47 to 5.2)	0
	No*	12	2,105	0.59	(0.26 to 0.92)				24	2,098	1.15	(0.69 to 1.61)			
Abnormal (NT-pro)BNP[±]	Yes	4	260	1.59	(0.07 to 3.11)	3.76	(1.44 to 9.81)	0	6	258	2.44	(0.56 to 4.32)	3.19	(1.5 to 6.77)	0
	No*	11	2,554	0.43	(0.18 to 0.68)				23	2,545	0.89	(0.52 to 1.26)			
Signs of RV overload[§]	Yes	7	369	1.93	(0.53 to 3.33)	2.27	(0.78 to 6.6)	0	10	368	2.85	(1.15 to 4.55)	1.77	(0.76 to 4.13)	0
	No*	10	1,427	0.69	(0.26 to 1.12)				18	1,418	1.23	(0.66 to 1.8)			
RV dysfunction on TTE	Yes	1	32	2.00	(0 to 6.85)	0.56	(0.33 to 0.95)	0	1	32	2.57	(0 to 8.09)	0.41	(0.24 to 0.73)	0
	No*	11	1,604	0.71	(0.3 to 1.12)				19	1,592	1.20	(0.66 to 1.74)			
Symptoms	Symptomatic*	16	2,111	0.76	(0.39 to 1.13)	0.65	(0.06 to 7.04)	0	27	2,106	1.28	(0.8 to 1.76)	0.86	(0.04 to 20.59)	0
	Incidental	2	79	2.54	(0 to 6.02)				3	80	3.77	(0 to 7.95)			
Age	18-40*	5	668	0.75	(0.1 to 1.4)				10	668	1.50	(0.58 to 2.42)			
	41-60	12	1,288	0.93	(0.41 to 1.45)	0.91	(0.58 to 1.43)	0	17	1,286	1.32	(0.7 to 1.94)	0.80	(0.5 to 1.28)	0
	61-80	6	1,155	0.52	(0.11 to 0.93)	0.68	(0.36 to 1.29)	0	15	1,148	1.31	(0.65 to 1.97)	0.76	(0.49 to 1.19)	0
	>81	2	130	1.54	(0 to 3.66)	1.28	(0.56 to 2.89)	0	2	128	1.56	(0 to 3.71)	1.00	(0.48 to 2.06)	0

I^2 was 0% and 0 for all proportion analysis * RR presents the ratio of the risk for an event for the exposure group to the risk for the non-exposure/reference group; non-exposure/reference group is marked with an asterisk # (1) Current diagnosis of cancer, (2) receiving treatment for cancer or (3) not receiving treatment for cancer and not in complete response ; ^Estimated Glomerular Filtration Rate < 60 ml/min; ¥Preexisting pulmonary disease was defined as a history of chronic obstructive pulmonary disease, asthma, or lung fibrosis, a preexisting cardiovascular disease, defined as any of coronary artery disease, heart failure, congenital heart disease, cardiomyopathy or rheumatic heart disease; ΩAbnormal troponin was defined as a troponin level >99th percentile according to local technique; ± NT-proBNP > 500 ng/L or BNP level >100 ng/L ; §Right ventricle/ left ventricle ratio >0.9 on computed tomography pulmonary angiogram or echocardiogram; Abbreviations: CI, confidence interval; DOAC direct oral anticoagulant; LMWH low molecular weight heparin; MB, major bleeding; NA not applicable; PI prediction interval; RR relative risk; RV right ventricle; TTE trans thoracic echocardiography; VKA vitamin K antagonist; VTE, venous thromboembolism.

Table S8: overall and subgroup analysis at 14- and 30-days for recurrent VTE incidence: sensitivity analysis according to the definition of home-treatment of the original studies

This table depicts the recurrent VTE outcomes of all patients that received home-treatment according to the definition of home-treatment of the original studies (discharge within 120 hours)

		Recurrent VTE at 14 days							Recurrent VTE at 30 days						
**subgroup variable**	**subgroup level**	**Events (n)**	**Patients (n)**	**%**	**(95%CI)**	**RR**	**(95%PI)**	**I2**	**Events (n)**	**Patients (n)**	**%**	**(95%CI)**	**RR**	**(95%PI)**	**I2**
overall		14	3,242	0.43	(0.2 to 0.66)				21	3,231	0.65	(0.37 to 0.93)			
Sex	Female	4	1,540	0.26	(0.01 to 0.51)	0.75	(0.36 to 1.55)	0	8	1,535	0.52	(0.16 to 0.88)	0.85	(0.43 to 1.68)	0
	Male*	10	1,702	0.59	(0.23 to 0.95)				13	1,696	0.77	(0.35 to 1.19)			
Cancer#	Yes	1	313	0.32	(0 to 0.95)	0.56	(0.34 to 0.9)	0	2	307	0.65	(0 to 1.55)	1.87	(0.09 to 38.2)	9.4
	No*	13	2,929	0.44	(0.2 to 0.68)				19	2,924	0.65	(0.36 to 0.94)			
Previous VTE	Yes	8	944	0.82	(0.25 to 1.39)	2.56	(0.95 to 6.87)	0	12	940	1.28	(0.56 to 2)	3.15	(1.31 to 7.62)	0
	No*	5	2,168	0.24	(0.03 to 0.45)				8	2,162	0.37	(0.12 to 0.62)			
Decreased kidney function^	Yes	1	264	0.40	(0 to 1.17)	0.63	(0.35 to 1.15)	0	1	262	0.45	(0 to 1.26)	0.53	(0.28 to 1)	0
	No*	13	2,978	0.43	(0.19 to 0.67)				20	2,969	0.67	(0.38 to 0.96)			
Preexisting cardio-pulmonary disease¥	Yes	7	589	1.26	(0.36 to 2.16)	3.31	(1.66 to 6.61)	0	8	586	1.41	(0.45 to 2.37)	2.49	(1.25 to 4.96)	0
	No*	7	2,653	0.25	(0.06 to 0.44)				13	2,645	0.48	(0.22 to 0.74)			
Treatment	DOAC*	7	1,938	0.36	(0.09 to 0.63)	0.78	(0.25 to 2.39)	0	11	1,935	0.57	(0.23 to 0.91)	0.67	(0.12 to 3.86)	0
	LMWH or VKA	6	1,174	0.51	(0.1 to 0.92)				9	1,167	0.77	(0.27 to 1.27)			
Abnormal troponinΩ	Yes	1	267	0.55	(0 to 1.44)	0.97	(0.58 to 1.62)	0	3	266	0.94	(0 to 2.1)	2.00	(0.69 to 5.85)	0
	No*	8	2,106	0.36	(0.11 to 0.61)				12	2,099	0.60	(0.27 to 0.93)			
Abnormal (NT-pro)BNP±	Yes	2	260	0.74	(0 to 1.78)	1.14	(0.68 to 1.91)	0	2	259	0.90	(0 to 2.05)	1.05	(0.55 to 2.02)	0
	No*	6	2,556	0.24	(0.05 to 0.43)				11	2,548	0.42	(0.17 to 0.67)			
Signs of RV overload§	Yes	4	370	1.18	(0.08 to 2.28)	3.48	(0.9 to 13.43)	0	5	368	1.46	(0.24 to 2.68)	2.57	(0.26 to 25.48)	9.4
	No*	4	1,427	0.26	(0 to 0.52)				6	1,419	0.40	(0.07 to 0.73)			
RV dysfunction on TTE	Yes	1	33	1.73	(0 to 6.16)	0.69	(0.4 to 1.19)	0	1	33	1.94	(0 to 6.65)	0.61	(0.29 to 1.27)	0
	No*	8	1,606	0.52	(0.17 to 0.87)				12	1,597	0.77	(0.34 to 1.2)			
Symptoms	Symptomatic*	8	2,107	0.38	(0.12 to 0.64)	0.21	(0 to 9.4)	0	12	2,102	0.57	(0.25 to 0.89)	0.21	(0 to 9.45)	0
	Incidental	0	79	0.00	(0 to 0)				0	79	0.00	(0 to 0)			
Age	18-40*	4	670	0.60	(0.02 to 1.18)				5	669	0.75	(0.1 to 1.4)			
	41-60	6	1,285	0.47	(0.1 to 0.84)	0.86	(0.54 to 1.37)	0	9	1,285	0.70	(0.24 to 1.16)	0.96	(0.52 to 1.78)	0
	61-80	3	1,157	0.26	(0 to 0.55)	0.63	(0.35 to 1.14)	0	6	1,148	0.52	(0.1 to 0.94)	0.77	(0.49 to 1.21)	0
	>81	1	129	0.78	(0 to 2.29)	0.94	(0.49 to 1.81)	0	1	128	0.78	(0 to 2.31)	0.90	(0.46 to 1.79)	0

I^2 was 0% and 0 for all proportion analysis * RR presents the ratio of the risk for an event for the exposure group to the risk for the non-exposure/reference group; non-exposure/reference group is marked with an asterisk # (1) Current diagnosis of cancer, (2) receiving treatment for cancer or (3) not receiving treatment for cancer and not in complete response ; ^Estimated Glomerular Filtration Rate < 60 ml/min; ¥Preexisting pulmonary disease was defined as a history of chronic obstructive pulmonary disease, asthma, or lung fibrosis, a preexisting cardiovascular disease, defined as any of coronary artery disease, heart failure, congenital heart disease, cardiomyopathy or rheumatic heart disease; ΩAbnormal troponin was defined as a troponin level >99th percentile according to local technique; ± NT-proBNP > 500 ng/L or BNP level >100 ng/L ; §Right ventricle/ left ventricle ratio >0.9 on computed tomography pulmonary angiogram or echocardiogram; Abbreviations: CI, confidence interval; DOAC direct oral anticoagulant; LMWH low molecular weight heparin; MB, major bleeding; NA not applicable; PI prediction interval; RR relative risk; RV right ventricle; TTE trans thoracic echocardiography; VKA vitamin K antagonist; VTE, venous thromboembolism.

Table S9: overall and subgroup analysis at 14- and 30-days for major bleeding: sensitivity analysis according to the definition of home-treatment of the original studies

This table depicts the major bleeding outcomes of all patients that received home-treatment according to the definition of home-treatment of the original studies (discharge within 120 hours)

		Major bleeding at 14 days							Major bleeding at 30 days						
**subgroup variable**	**subgroup level**	**Events (n)**	**Patients (n)**	**%**	**(95%CI)**	**RR**	**(95%PI)**	**I2**	**Events (n)**	**Patients (n)**	**%**	**(95%CI)**	**RR**	**(95%PI)**	**I2**
overall		9	3,237	0.28	(0.1 to 0.46)				17	3,223	0.53	(0.28 to 0.78)			
Sex	Female	6	1,539	0.39	(0.08 to 0.7)	1.51	(0.84 to 2.72)	0	12	1,530	0.78	(0.34 to 1.22)	1.93	1.07 to 3.49	0
	Male*	3	1,698	0.18	(0 to 0.38)				5	1,693	0.30	(0.04 to 0.56)			
Cancer#	Yes	2	311	0.64	(0 to 1.53)	1.94	(0.84 to 4.48)	0	4	303	1.32	(0.03 to 2.61)	2.39	0.54 to 10.69	3.29
	No*	7	2,926	0.24	(0.06 to 0.42)				13	2,920	0.45	(0.21 to 0.69)			
Previous VTE	Yes	0	943	0.00	(0 to 0)	0.52	(0.33 to 0.82)	0	1	937	0.11	(0 to 0.32)	0.46	0.26 to 0.8	0
	No*	8	2,164	0.37	(0.11 to 0.63)				15	2,157	0.70	(0.35 to 1.05)			
Decreased kidney function^	Yes	0	264	0.17	(0 to 0.67)	0.68	(0.42 to 1.11)	0	0	262	0.18	(0 to 0.69)	0.49	0.26 to 0.9	0
	No*	9	2,973	0.29	(0.1 to 0.48)				17	2,961	0.56	(0.29 to 0.83)			
Preexisting cardio-pulmonary disease¥	Yes	2	586	0.31	(0 to 0.76)	1.11	(0.56 to 2.2)	0	4	585	0.64	(0 to 1.29)	1.25	0.68 to 2.32	0
	No*	7	2,651	0.27	(0.07 to 0.47)				13	2,638	0.50	(0.23 to 0.77)			
Treatment	DOAC*	3	1,936	0.15	(0 to 0.33)	1.65	(0.64 to 4.26)	0	7	1,932	0.36	(0.09 to 0.63)	1.89	0.76 to 4.68	0
	LMWH or VKA	5	1,171	0.43	(0.06 to 0.8)				9	1,162	0.77	(0.27 to 1.27)			
Abnormal troponinΩ	Yes	3	267	0.97	(0 to 2.15)	3.42	(1.38 to 8.5)	0	4	265	1.39	(0 to 2.8)	2.48	1.44 to 4.27	0
	No*	3	2,101	0.16	(0 to 0.33)				8	2,091	0.40	(0.13 to 0.67)			
Abnormal (NT-pro)BNP±	Yes	3	260	1.10	(0 to 2.37)	4.76	(1.56 to 14.49)	0	4	257	1.54	(0.04 to 3.04)	4.11	1.79 to 9.45	0
	No*	3	2,552	0.12	(0 to 0.26)				8	2,543	0.32	(0.1 to 0.54)			
Signs of RV overload§	Yes	2	369	0.68	(0 to 1.52)	1.68	(0.29 to 9.67)	0.84	4	367	0.97	(0 to 1.98)	1.22	0.63 to 2.37	0
	No*	5	1,423	0.32	(0.03 to 0.61)				8	1,414	0.60	(0.2 to 1)			
RV dysfunction on TTE	Yes	0	32	0.29	(0 to 2.16)	NA	NA	0	0	31	0.47	(0 to 2.85)	0.66	0.39 to 1.13	0
	No*	3	1,601	0.18	(0 to 0.39)				5	1,590	0.31	(0.04 to 0.58)			
Symptoms	Symptomatic*	7	2,108	0.33	(0.08 to 0.58)	1.61	(0 to 6892.43)	0	12	2,101	0.57	(0.25 to 0.89)	2.29	0 to 98662.56	0
	Incidental	1	78	1.29	(0 to 3.8)				2	79	2.54	(0 to 6.02)			
Age	18-40*	1	667	0.15	(0 to 0.44)				4	666	0.60	(0.01 to 1.19)			
	41-60	4	1,285	0.31	(0.01 to 0.61)	1.01	(0.53 to 1.92)	0	6	1,283	0.47	(0.1 to 0.84)	0.77	0.44 to 1.35	0
	61-80	3	1,155	0.26	(0 to 0.55)	1.00	(0.54 to 1.86)	0	6	1,145	0.52	(0.1 to 0.94)	0.75	0.45 to 1.26	0
	>81	1	129	0.78	(0 to 2.29)	1.29	(0.8 to 2.08)	0	1	128	0.78	(0 to 2.31)	1.06	0.59 to 1.9	0

I^2 was 0% for all proportion analysis * RR presents the ratio of the risk for an event for the exposure group to the risk for the non-exposure/reference group; non-exposure/reference group is marked with an asterisk # (1) Current diagnosis of cancer, (2) receiving treatment for cancer or (3) not receiving treatment for cancer and not in complete response ; ^Estimated Glomerular Filtration Rate < 60 ml/min; ¥Preexisting pulmonary disease was defined as a history of chronic obstructive pulmonary disease, asthma, or lung fibrosis, a preexisting cardiovascular disease, defined as any of coronary artery disease, heart failure, congenital heart disease, cardiomyopathy or rheumatic heart disease; ΩAbnormal troponin was defined as a troponin level >99th percentile according to local technique; ± NT-proBNP > 500 ng/L or BNP level >100 ng/L ; §Right ventricle/ left ventricle ratio >0.9 on computed tomography pulmonary angiogram or echocardiogram; Abbreviations: CI, confidence interval; DOAC direct oral anticoagulant; LMWH low molecular weight heparin; MB, major bleeding; NA not applicable; PI prediction interval; RR relative risk; RV right ventricle; TTE trans thoracic echocardiography; VKA vitamin K antagonist; VTE, venous thromboembolism.

Sensitivity analysis: Font et al included (discharge within 24 hours)

Table S10: characteristics patients that received home treatment (defined as discharge within 24 hours) including Font et al.

Study, year (reference)	Overall	Zondag et al., 2011	Exter et al., 2016	Roy et al., 2021	Kline et al., 2021	Barco et al., 2020	Kabrhel et al., 2019	Font et al., 2014	Vinson et al., 2018	Bledsoe et al., 2018
Patients, n	2756	296	513	681	604	170	122	62	116	192
Mean age (SD)	54.0 (16.0)	54.5(15.4)	53.5(14.7)	56.4 (16.2)	52.0(16.6)	54.5 (16.0)	55.4 (16.4)	62.5 (10.3)	60.3 (15.1)	44.4 (14.3)
Female, n (%)	1307 (47%)	124 (42%)	235 (46%)	314 (46%)	301 (50%)	80 (47%)	64 (52%)	25 (40%)	61 (53%)	103 (54%)
Triage tool applied, n (%)										
Negative Hestia (or Hestia like) rule	1685 (61%)	296 (100%)	513 (100%)	351 (51%)	463 (77%)	0 (0%)	0 (0%)	62 (100%)	0 (0%)	0 (0%)
sPESI 0 or PESI I/II$^{\alpha}$	471 (17%)	0 (0%)	0 (0%)	330 (49%)	141 (23%)	0 (0%)	0 (0%)	0 (0%)	0 (0%)	0 (0%)
sPESI 0 or PESI I/II and absence of RVD$^{\alpha}$	308 (11%)	0 (0%)	0 (0%)	0 (0%)	0 (0%)	0 (0%)	0 (0%)	0 (0%)	116 (100%)	192 (100%)
Negative Hestia (lor Hestia like) criteria and absence of RVD	170 (6.2%)	0 (0%)	0 (0%)	0 (0%)	0 (0%)	170 (100%)	0 (0%)	0 (0%)	0 (0%)	0 (0%)
Other tool	122 (4.4%)	0 (0%)	0 (0%)	0 (0%)	0 (0%)	0 (0%)	122 (100%)	0 (0%)	0 (0%)	0 (0%)
Treatment with a DOAC, n (%)	1550 (59%)	0 (0%)	3 (1%)	565 (83%)	604 (100%)	170 (100%)	43 (35%)	0 (0%)	*	165 (86%)
Risk factors, n (%)										
Recent immobilization or surgery	300 (12%)	27 (9%)	69 (13%)	68 (10%)	54 (9%)	26 (15%)	17 (14%)	39 (63%)	*	*
Estrogen use	297 (12%)	47 (16%)	91 (18%)	64 (9%)	41 (7%)	31 (18%)	8 (7%)	8 (13%)	8 (7%)	*
Symptomatic PE, n (%)	1659 (96%)	296 (100%)	513 (100%)	681 (100%)	*	155 (91%)	*	14 (23%)	*	*
Vital signs at presentation€, n (%)										
Heart rate ≥110/min	223 (8%)	27 (9%)	35 (7%)	63 (9%)	62 (10%)	3 (2%)	*	0 (0%)	15 (13%)	18 (9%)
Respiratory rate of ≥ 30/min	13 (1%)	*	*	*	5 (1%)	2 (1%)	*	*	4 (3%)	2 (1%)
Oxygen saturation <90% or need for oxygen	54 (2%)	19 (6%)	4 (1%)	23 (3%)	2 (0%)	1 (1%)	*	0 (0%)	5 (4%)	0 (0%)
Comorbidities, n (%)										
Cancer#	289 (10%)	28 (9%)	34 (7%)	50 (7%)	24 (4%)	19 (11%)	55 (45%)	62 (100%)	16 (14%)	2 (1%)
Previous VTE	846 (32%)	74 (25%)	120 (23%)	169 (25%)	375 (62%)	34 (20%)	34 (28%)	4 (6%)	*	37 (19%)
Decreased kidney function^	210 (8%)	12 (4%)	22 (4%)	66 (10%)	67 (11%)	12 (7%)	11 (9%)	2 (3%)	11 (9%)	6 (3%)
Preexisting cardio-pulmonary disease¥	504 (18%)	12 (4%)	25 (5%)	161 (24%)	186 (31%)	12 (7%)	30 (25%)	17 (27%)	28 (24%)	32 (17%)
Laboratory/imaging results, n (%)										
Abnormal troponin$^{\Omega}$	251 (11%)	*	67 (13%)	117 (17%)	53 (9%)	*	4 (3%)	*	9 (8%)	1 (1%)
Abnormal (NT-pro)BNP$^{\pm}$	234 (10%)	*	27 (5%)	78 (11%)	80 (13%)	10 (6%)	11 (9%)	*	21 (18%)	6 (3%)
RV overload§	351 (26%)	201 (68%)	*	106 (16%)	*	9 (5%)	15 (12%)	21 (34%)	*	*
RVD on echocardiography	31 (3%)	*	*	*	20 (3%)	0 (0%)	6 (5%)	*	3 (3%)	2 (1%)

* Variable systematically missing within a study. $^{\alpha}$ in combination with a negative clinical judgement € Vinson et al. reported the worst vital signs throughout the hole of the patients' ED stay # cancer was defined as (1) a current diagnosis of cancer, (2) receiving treatment for cancer or (3) not receiving treatment for cancer and not in complete response; ^Estimated Glomerular Filtration Rate < 60 ml/min; ¥Preexisting pulmonary disease was defined as a history of chronic obstructive pulmonary disease, asthma, or lung fibrosis, a preexisting cardiovascular disease, defined as any of coronary artery disease, heart failure, congenital heart disease, cardiomyopathy or rheumatic heart disease ; ΩAbnormal troponin was defined as a troponin level >99th percentile according to local technique; ± NT-proBNP > 500 ng/L or BNP level >100 ng/L ; §Right ventricle/ left ventricle ratio >0.9 on computed tomography pulmonary angiogram or echocardiogram. Abbreviation: DOAC direct oral anticoagulant; PE pulmonary embolism; RV right ventricular; RVD right ventricle dysfunction SD standard deviation; VTE venous thromboembolism; RV/LV right ventricle/left ventricle

Table S11: overall and subgroup analysis at 14- and 30-days for mortality: sensitivity analysis with Font et al included

This table depicts the mortality outcomes of all patients that were discharged within 24 hours

		mortality at 14 days							mortality at 30 days						
**subgroup variable**	**subgroup level**	**Events (n)**	**Patients (n)**	**%**	**(95%CI)**	**RR**	**(95%PI)**	**I2**	**Events (n)**	**Patients (n)**	**%**	**(95%CI)**	**RR**	**(95%PI)**	**I2**
overall		5	2726	0.18	(0.02 to 0.34)				10	2722	0.37	(0.14 to 0.6)			
Sex	Male*	3	1435	0.21	(0 to 0.45)	1.03	(0.49 to 2.16)	0	4	1433	0.28	(0.01 to 0.55)	1.40	(0.72 to 2.7)	0
	Female	2	1291	0.15	(0 to 0.36)				6	1289	0.47	(0.1 to 0.84)			
Cancer#	Yes	3	281	1.07	(0 to 2.27)	2.88	(0.8 to 10.36)	0	6	277	2.16	(0.45 to 3.87)	4.94	(2.69 to 9.08)	0
	No*	2	2445	0.08	(0 to 0.19)				4	2445	0.16	(0 to 0.32)			
Previous VTE	Yes	1	835	0.12	(0 to 0.35)	1.23	(0.51 to 2.94)	0	3	835	0.36	(0 to 0.77)	1.72	(0.6 to 4.94)	0
	No*	4	1775	0.23	(0.01 to 0.45)				6	1771	0.34	(0.07 to 0.61)			
Decreased kidney function^	Yes	0	205	0.00	(0 to 0)	0.74	(0.54 to 1.03)	0	0	205	0.18	(0 to 0.75)	0.63	(0.39 to 1.02)	0
	No*	5	2521	0.20	(0.03 to 0.37)				10	2517	0.38	(0.14 to 0.62)			
Preexisting cardio-pulmonary disease¥	Yes	1	497	0.27	(0 to 0.72)	1.85	(0.3 to 11.54)	5.8	2	495	0.33	(0 to 0.83)	1.40	(0.23 to 8.55)	7.1
	No*	4	2229	0.16	(0 to 0.33)				8	2227	0.38	(0.13 to 0.63)			
Treatment	DOAC*	0	1534	0.00	(0 to 0)	3.12	(0.17 to 55.93)	0	1	1533	0.07	(0 to 0.2)	2.62	(0.91 to 7.49)	0
	LMWH or VKA	5	1076	0.46	(0.05 to 0.87)				8	1073	0.75	(0.24 to 1.26)			
Abnormal troponinΩ	Yes	0	249	0.14	(0 to 0.6)	0.86	(0.56 to 1.32)	0	1	249	0.60	(0 to 1.56)	2.19	(0.59 to 8.13)	0
	No*	2	1950	0.08	(0 to 0.21)				5	1947	0.23	(0.02 to 0.44)			
Abnormal (NT-pro)BNP±	Yes	0	210	0.10	(0 to 0.53)	0.93	(0.75 to 1.14)	0	1	210	0.40	(0 to 1.25)	0.84	(0.52 to 1.35)	0
	No*	2	2158	0.08	(0 to 0.2)				5	2154	0.24	(0.03 to 0.45)			
Signs of RV overload§	Yes	1	348	0.43	(0 to 1.12)	0.88	(0.27 to 2.88)	0	3	347	0.80	(0 to 1.74)	0.99	(0.52 to 1.89)	0
	No*	3	954	0.26	(0 to 0.58)				4	951	0.44	(0.02 to 0.86)			
RV dysfunction on TTE	Yes	0	30	0.00	(0 to 0)	NA	NA		0	30	0.18	(0 to 1.7)	0.71	(0.41 to 1.24)	
	No*	0	1147	0.00	(0 to 0)				2	1143	0.17	(0 to 0.41)			
Symptoms	Incidental *	2	63	3.17	(0 to 7.5)	2.51	(0 to 3646.64)	0	2	63	3.17	(0 to 7.5)	2.50	(0 to 3672.75)	0
	Symptomatic	3	1655	0.18	(0 to 0.38)				6	1654	0.36	(0.07 to 0.65)			
Age	18-40*	0	583	0.00	(0 to 0)				1	583	0.17	(0 to 0.51)			
	41-60	4	1109	0.36	(0.01 to 0.71)	1.30	(0.93 to 1.82)	0	4	1108	0.36	(0.01 to 0.71)	0.95	(0.5 to 1.79)	0
	61-80	1	931	0.11	(0 to 0.32)	1.13	(0.88 to 1.46)	0	5	928	0.54	(0.07 to 1.01)	1.24	(0.58 to 2.65)	0
	>81	0	102	0.00	(0 to 0)	NA	NA		0	102	0.00	(0 to 0)	0.86	(0.66 to 1.12)	0

I^2 was 0% and 0 for all proportion analysis * RR presents the ratio of the risk for an event for the exposure group to the risk for the non-exposure/reference group; non-exposure/reference group is marked with an asterisk # (1) Current diagnosis of cancer, (2) receiving treatment for cancer or (3) not receiving treatment for cancer and not in complete response ; ^Estimated Glomerular Filtration Rate < 60 ml/min; ¥Preexisting pulmonary disease was defined as a history of chronic obstructive pulmonary disease, asthma, or lung fibrosis, a preexisting cardiovascular disease, defined as any of coronary artery disease, heart failure, congenital heart disease, cardiomyopathy or rheumatic heart disease; ΩAbnormal troponin was defined as a troponin level >99th percentile according to local technique; ± NT-proBNP > 500 ng/L or BNP level >100 ng/L ; §Right ventricle/ left ventricle ratio >0.9 on computed tomography pulmonary angiogram or echocardiogram; Abbreviations: CI, confidence interval; DOAC direct oral anticoagulant; LMWH low molecular weight heparin; MB, major bleeding; NA not applicable; PI prediction interval; RR relative risk; RV right ventricle; TTE trans thoracic echocardiography; VKA vitamin K antagonist; VTE, venous thromboembolism.

Table S12: overall and subgroup analysis at 14- and 30-days for all adverse events (i.e. combined endpoint of recurrent VTE, MB or mortality): sensitivity analysis with Font et al included

This table depicts the mortality outcomes of all patients that received home-treatment according to the definition of home-treatment of the original studies (discharge within 24 hours)

		**combined endpoint of recurrent VTE, MB or mortality) at 14 days**							**combined endpoint of recurrent VTE, MB or mortality) at 30 days**						
**subgroup variable**	**subgroup level**	**Events (n)**	**Patients (n)**	**%**	**(95%CI)**	**RR**	**(95%PI)**	**I2**	**Events (n)**	**Patients (n)**	**%**	**(95%CI)**	**RR**	**(95%PI)**	**I2**
overall		18	2720	0.66	(0.36 to 0.96)				36	2714	1.33	(0.9 to 1.76)			
Sex	Male*	12	1431	0.84	(0.37 to 1.31)	0.94	(0.41 to 2.12)	0	18	1429	1.26	(0.68 to 1.84)	1.25	(0.48 to 3.24)	7.4
	Female	6	1289	0.47	(0.1 to 0.84)				18	1285	1.40	(0.76 to 2.04)			
Cancer[#]	Yes	4	277	1.44	(0.04 to 2.84)	1.65	(0.7 to 3.9)	0	9	272	3.30	(1.18 to 5.42)	2.70	(1.4 to 5.22)	0
	No*	14	2443	0.57	(0.27 to 0.87)				27	2442	1.11	(0.7 to 1.52)			
Previous VTE	Yes	8	834	0.92	(0.27 to 1.57)	1.89	(0.8 to 4.46)	0	14	833	1.69	(0.82 to 2.56)	1.53	(0.79 to 2.98)	0
	No*	10	1770	0.58	(0.23 to 0.93)				21	1765	1.19	(0.68 to 1.7)			
Decreased kidney function[^]	Yes	1	205	0.30	(0 to 1.05)	0.46	(0.24 to 0.9)	0	1	204	0.54	(0 to 1.55)	0.34	(0.15 to 0.78)	0
	No*	17	2515	0.69	(0.37 to 1.01)				35	2510	1.39	(0.93 to 1.85)			
Preexisting cardio-pulmonary disease[¥]	Yes	6	495	1.26	(0.28 to 2.24)	2.81	(1.08 to 7.27)	0	9	493	1.89	(0.69 to 3.09)	1.61	(0.85 to 3.03)	0
	No*	12	2225	0.53	(0.23 to 0.83)				27	2221	1.20	(0.75 to 1.65)			
Treatment	DOAC*	8	1532	0.52	(0.16 to 0.88)	1.34	(0.78 to 2.3)	0	17	1530	1.11	(0.58 to 1.64)	1.44	(0.72 to 2.89)	0
	LMWH or VKA	10	1072	0.93	(0.35 to 1.51)				18	1068	1.69	(0.92 to 2.46)			
Abnormal troponin[Ω]	Yes	3	249	1.23	(0 to 2.6)	2.46	(1.25 to 4.86)	0	6	248	2.55	(0.59 to 4.51)	2.87	(1.45 to 5.69)	0
	No*	8	1946	0.41	(0.13 to 0.69)				19	1941	0.96	(0.53 to 1.39)			
Abnormal (NT-pro)BNP[±]	Yes	3	210	1.60	(0 to 3.3)	3.94	(1.58 to 9.79)	0	6	208	2.66	(0.47 to 4.85)	3.32	(1.56 to 7.06)	0
	No*	8	2154	0.35	(0.1 to 0.6)				19	2149	0.91	(0.51 to 1.31)			
Signs of RV overload[§]	Yes	7	346	1.98	(0.51 to 3.45)	2.28	(0.83 to 6.29)	0	10	345	2.97	(1.18 to 4.76)	1.77	(0.79 to 3.98)	0
	No*	4	950	0.44	(0.02 to 0.86)				11	946	1.14	(0.46 to 1.82)			
RV dysfunction on TTE	Yes	0	29	1.67	(0 to 6.35)	0.71	(0.26 to 1.97)		1	28	2.30	(0 to 7.81)	0.47	(0.15 to 1.5)	
	No*	5	1144	0.40	(0.04 to 0.76)				11	1138	1.00	(0.42 to 1.58)			
Symptoms	Incidental *	2	61	3.28	(0 to 7.75)	0.70	(0.18 to 2.69)	0	3	62	4.84	(0 to 10.18)	0.92	(0.68 to 1.25)	0
	Symptomatic	11	1655	0.66	(0.27 to 1.05)				21	1654	1.27	(0.73 to 1.81)			
Age	18-40*	3	581	0.52	(0 to 1.1)				8	581	1.38	(0.43 to 2.33)			
	41-60	10	1109	0.90	(0.34 to 1.46)	1.11	(0.69 to 1.79)	0	14	1107	1.26	(0.6 to 1.92)	0.83	(0.48 to 1.44)	0
	61-80	5	927	0.54	(0.07 to 1.01)	0.97	(0.51 to 1.83)	0	14	923	1.52	(0.73 to 2.31)	0.92	(0.63 to 1.33)	0
	>81	0	102	0.00	(0 to 0)	0.80	(0.56 to 1.13)	0	0	102	0.00	(0 to 0)	0.53	(0.34 to 0.82)	0

I^2 was 0% and 0 for all proportion analysis * RR presents the ratio of the risk for an event for the exposure group to the risk for the non-exposure/reference group; non-exposure/reference group is marked with an asterisk # (1) Current diagnosis of cancer, (2) receiving treatment for cancer or (3) not receiving treatment for cancer and not in complete response ; ^Estimated Glomerular Filtration Rate < 60 ml/min; ¥Preexisting pulmonary disease was defined as a history of chronic obstructive pulmonary disease, asthma, or lung fibrosis, a preexisting cardiovascular disease, defined as any of coronary artery disease, heart failure, congenital heart disease, cardiomyopathy or rheumatic heart disease; ΩAbnormal troponin was defined as a troponin level >99th percentile according to local technique; ± NT-proBNP > 500 ng/L or BNP level >100 ng/L ; §Right ventricle/ left ventricle ratio >0.9 on computed tomography pulmonary angiogram or echocardiogram; Abbreviations: CI, confidence interval; DOAC direct oral anticoagulant; LMWH low molecular weight heparin; MB, major bleeding; NA not applicable; PI prediction interval; RR relative risk; RV right ventricle; TTE trans thoracic echocardiography; VKA vitamin K antagonist; VTE, venous thromboembolism.

Table S13: overall and subgroup analysis at 14- and 30-days for recurrent VTE: sensitivity analysis with Font et al included

This table depicts the recurrent VTE outcomes of all patients that received home-treatment according to the definition of home-treatment of the original studies (discharge within 24 hours)

		Recurrent VTE at 14 days							Recurrent VTE at 30 days						
**subgroup variable**	**subgroup level**	**Events (n)**	**Patients (n)**	**%**	**(95%CI)**	**RR**	**(95%PI)**	**I2**	**Events (n)**	**Patients (n)**	**%**	**(95%CI)**	**RR**	**(95%PI)**	**I2**
overall		10	2721	0.37	(0.14 to 0.6)				16	2714	0.59	(0.3 to 0.88)			
Sex	Male*	8	1432	0.56	(0.17 to 0.95)	0.70	(0.32 to 1.53)	0	11	1429	0.77	(0.32 to 1.22)	0.74	(0.37 to 1.51)	0
	Female	2	1289	0.16	(0 to 0.37)				5	1285	0.39	(0.05 to 0.73)			
Cancer[#]	Yes	1	278	0.36	(0 to 1.06)	0.71	(0.41 to 1.22)	0	1	272	0.37	(0 to 1.09)	0.61	(0.31 to 1.17)	0
	No*	9	2443	0.37	(0.13 to 0.61)				15	2442	0.61	(0.3 to 0.92)			
Previous VTE	Yes	7	834	0.80	(0.19 to 1.41)	2.33	(0.81 to 6.67)	0	11	833	1.33	(0.55 to 2.11)	3.02	(1.21 to 7.57)	0
	No*	3	1771	0.19	(0 to 0.39)				5	1766	0.28	(0.03 to 0.53)			
Decreased kidney function[^]	Yes	1	205	0.30	(0 to 1.05)	0.63	(0.35 to 1.15)	0	1	205	0.36	(0 to 1.18)	0.53	(0.28 to 1)	0
	No*	9	2516	0.37	(0.13 to 0.61)				15	2509	0.61	(0.31 to 0.91)			
Preexisting cardio-pulmonary disease[¥]	Yes	4	496	0.89	(0.06 to 1.72)	2.39	(1.02 to 5.64)	0	5	495	1.07	(0.16 to 1.98)	1.89	(0.9 to 3.98)	0
	No*	6	2225	0.25	(0.04 to 0.46)				11	2219	0.48	(0.19 to 0.77)			
Treatment	DOAC*	6	1534	0.39	(0.08 to 0.7)	0.78	(0.26 to 2.38)	0	10	1533	0.65	(0.25 to 1.05)	0.68	(0.12 to 3.85)	0
	LMWH or VKA	4	1071	0.37	(0 to 0.74)				6	1066	0.56	(0.11 to 1.01)			
Abnormal troponin[Ω]	Yes	1	248	0.39	(0 to 1.17)	0.89	(0.74 to 1.08)	0	2	247	0.81	(0 to 1.93)	2.09	(0.63 to 6.9)	0
	No*	5	1949	0.26	(0.03 to 0.49)				9	1944	0.46	(0.16 to 0.76)			
Abnormal (NT-pro)BNP[±]	Yes	1	210	0.55	(0 to 1.55)	0.90	(0.74 to 1.08)	0	2	209	0.75	(0 to 1.92)	0.83	(0.53 to 1.3)	0
	No*	5	2156	0.23	(0.03 to 0.43)				9	2151	0.44	(0.16 to 0.72)			
Signs of RV overload[§]	Yes	4	346	1.26	(0.09 to 2.43)	3.50	(0.97 to 12.65)	0	5	345	1.56	(0.25 to 2.87)	2.58	(0.29 to 23.31)	8.5
	No*	1	952	0.07	(0 to 0.23)				2	947	0.17	(0 to 0.43)			
RV dysfunction on TTE	Yes	0	30	1.60	(0 to 6.1)	0.80	(0.45 to 1.43)		1	30	1.84	(0 to 6.67)	0.71	(0.3 to 1.69)	
	No*	5	1147	0.39	(0.03 to 0.75)				7	1142	0.65	(0.18 to 1.12)			
Symptoms	Incidental *	0	61	0.00	(0 to 0)	0.24	(0 to 408.36)	0	0	61	0.00	(0 to 0)	0.24	(0 to 408.36)	0
	Symptomatic	5	1652	0.30	(0.04 to 0.56)				8	1650	0.48	(0.14 to 0.82)			
Age	18-40*	3	583	0.51	(0 to 1.09)				4	582	0.69	(0.02 to 1.36)			
	41-60	4	1105	0.36	(0.01 to 0.71)	0.89	(0.56 to 1.43)	0	6	1105	0.54	(0.11 to 0.97)	0.93	(0.46 to 1.91)	0
	61-80	3	930	0.32	(0 to 0.68)	0.80	(0.47 to 1.34)	0	6	924	0.65	(0.13 to 1.17)	0.92	(0.69 to 1.23)	0
	>81	0	102	0.00	(0 to 0)	0.80	(0.56 to 1.13)	0	0	102	0.00	(0 to 0)	0.75	(0.5 to 1.14)	0

* RR presents the ratio of the risk for an event for the exposure group to the risk for the non-exposure/reference group; non-exposure/reference group is marked with an asterisk # (1) Current diagnosis of cancer, (2) receiving treatment for cancer or (3) not receiving treatment for cancer and not in complete response ; ^Estimated Glomerular Filtration Rate < 60 ml/min; ¥Preexisting pulmonary disease was defined as a history of chronic obstructive pulmonary disease, asthma, or lung fibrosis, a preexisting cardiovascular disease, defined as any of coronary artery disease, heart failure, congenital heart disease, cardiomyopathy or rheumatic heart disease; ΩAbnormal troponin was defined as a troponin level >99th percentile according to local technique; ± NT-proBNP > 500 ng/L or BNP level >100 ng/L ; §Right ventricle/ left ventricle ratio >0.9 on computed tomography pulmonary angiogram or echocardiogram; Abbreviations: CI, confidence interval; DOAC direct oral anticoagulant; LMWH low molecular weight heparin; MB, major bleeding; NA not applicable; PI prediction interval; RR relative risk; RV right ventricle; TTE trans thoracic echocardiography; VKA vitamin K antagonist; VTE, venous thromboembolism.

Table S14: overall and subgroup analysis at 14- and 30-days for major bleeding: sensitivity analysis with Font et al included

This table depicts the major bleeding outcomes of all patients that received home-treatment according to the definition of home-treatment of the original studies (discharge within 24 hours)

		Major bleeding at 14 days							Major bleeding at 30 days						
subgroup variable	subgroup level	Events (n)	Patients (n)	%	(95%CI)	RR	(95%PI)	I2	Events (n)	Patients (n)	%	(95%CI)	RR	(95%PI)	I2
overall		6	2718	0.22	(0.04 to 0.4)				14	2708	0.52	(0.25 to 0.79)			
Sex	Male*	3	1430	0.21	(0 to 0.45)	1.13	(0.63 to 2.02)	0	5	1427	0.35	(0.04 to 0.66)	1.62	(0.83 to 3.16)	0
	Female	3	1288	0.23	(0 to 0.49)				9	1281	0.70	(0.24 to 1.16)			
Cancer#	Yes	2	276	0.72	(0 to 1.72)	2.14	(0.98 to 4.68)	0	4	269	1.48	(0.04 to 2.92)	2.89	(1.22 to 6.87)	0
	No*	4	2442	0.16	(0 to 0.32)				10	2439	0.41	(0.16 to 0.66)			
Previous VTE	Yes	0	833	0.00	(0 to 0)	0.59	(0.34 to 1)	0	1	830	0.12	(0 to 0.36)	0.49	(0.26 to 0.95)	0
	No*	6	1769	0.34	(0.07 to 0.61)				13	1763	0.74	(0.34 to 1.14)			
Decreased kidney function^	Yes	0	205	0.00	(0 to 0)	0.67	(0.43 to 1.06)	0	0	204	0.01	(0 to 0.12)	0.45	(0.24 to 0.83)	0
	No*	6	2513	0.24	(0.05 to 0.43)				14	2504	0.56	(0.27 to 0.85)			
Preexisting cardio-pulmonary disease¥	Yes	1	494	0.17	(0 to 0.53)	0.81	(0.61 to 1.06)	0	3	493	0.56	(0 to 1.22)	1.06	(0.7 to 1.59)	0
	No*	5	2224	0.23	(0.03 to 0.43)				11	2215	0.51	(0.21 to 0.81)			
Treatment	DOAC*	2	1532	0.13	(0 to 0.31)	1.65	(0.63 to 4.33)	0	6	1530	0.39	(0.08 to 0.7)	1.89	(0.75 to 4.78)	0
	LMWH or VKA	4	1070	0.37	(0 to 0.74)				8	1063	0.75	(0.23 to 1.27)			
Abnormal troponinΩ	Yes	2	249	0.84	(0 to 1.98)	3.56	(1.26 to 10.07)	0	3	247	1.29	(0 to 2.7)	2.52	(1.37 to 4.62)	0
	No*	2	1945	0.10	(0 to 0.24)				7	1937	0.35	(0.09 to 0.61)			
Abnormal (NT-pro)BNP±	Yes	2	210	1.05	(0 to 2.43)	4.88	(1.58 to 15.06)	0	3	207	1.60	(0 to 3.31)	4.19	(1.82 to 9.67)	0
	No*	2	2153	0.08	(0 to 0.2)				7	2146	0.31	(0.07 to 0.55)			
Signs of RV overload§	Yes	2	346	0.72	(0 to 1.61)	1.70	(0.32 to 8.92)	0	4	343	1.04	(0 to 2.11)	1.22	(0.65 to 2.31)	0
	No*	3	949	0.26	(0 to 0.59)				6	945	0.68	(0.16 to 1.2)			
RV dysfunction on TTE	Yes	0	29	0.00	(0 to 0)	NA	NA	NA	0	28	0.19	(0 to 1.78)	NA	NA	NA
	No*	0	1144	0.00	(0 to 0)				2	1137	0.17	(0 to 0.41)			
Symptoms	Incidental *	1	60	1.67	(0 to 4.91)	1.83	(0.02 to 217.27)	0	2	61	3.28	(0 to 7.75)	2.54	(0 to 3664.24)	0
	Symptomatic	5	1654	0.30	(0.04 to 0.56)				10	1651	0.61	(0.24 to 0.98)			
Age	18-40*	0	581	0.00	(0 to 0)				3	580	0.52	(0 to 1.1)			
	41-60	4	1107	0.36	(0.01 to 0.71)	1.41	(0.91 to 2.18)	0	6	1106	0.54	(0.11 to 0.97)	0.90	(0.54 to 1.51)	0
	61-80	2	927	0.22	(0 to 0.52)	1.31	(0.93 to 1.85)	0	5	919	0.54	(0.06 to 1.02)	0.85	(0.56 to 1.29)	0
	>81	0	102	0.00	(0 to 0)	1.00	(1 to 1)	0	0	102	0.00	(0 to 0)	0.76	(0.56 to 1.04)	0

I^2 was 0% and 0 for all proportion analysis * RR presents the ratio of the risk for an event for the exposure group to the risk for the non-exposure/reference group; non-exposure/reference group is marked with an asterisk # (1) Current diagnosis of cancer, (2) receiving treatment for cancer or (3) not receiving treatment for cancer and not in complete response ; ^Estimated Glomerular Filtration Rate < 60 ml/min; ¥Preexisting pulmonary disease was defined as a history of chronic obstructive pulmonary disease, asthma, or lung fibrosis, a preexisting cardiovascular disease, defined as any of coronary artery disease, heart failure, congenital heart disease, cardiomyopathy or rheumatic heart disease; ΩAbnormal troponin was defined as a troponin level >99th percentile according to local technique; ± NT-proBNP > 500 ng/L or BNP level >100 ng/L ; §Right ventricle/ left ventricle ratio >0.9 on computed tomography pulmonary angiogram or echocardiogram; Abbreviations: CI, confidence interval; DOAC direct oral anticoagulant; LMWH low molecular weight heparin; MB, major bleeding; NA not applicable; PI prediction interval; RR relative risk; RV right ventricle; TTE trans thoracic echocardiography; VKA vitamin K antagonist; VTE, venous thromboembolism.

Sensitivity analysis according to the non-imputed data (discharge within 24 hours)

Table S15 : overall and subgroup analysis at 14- and 30-days for mortality: sensitivity analysis non-imputed data (discharge within 24 hours)

		mortality at 14 days							Mortality at 30 days						
subgroup variable	subgroup level	Events (n)	Patients (n)	%	(95%CI)	RR	(95%PI)	I2	Events (n)	Patients (n)	%	(95%CI)	RR	(95%PI)	I2
overall		3	2664	0.11	(0 to 0.24)				8	2660	0.30	(0.09 to 0.51)			
Sex	Male*	1	1398	0.07	(0 to 0.21)	1.28	(0.65 to 2.52)	0	2	1396	0.14	(0 to 0.34)	1.68	(0.98 to 2.86)	0
	Female	2	1266	0.16	(0 to 0.38)				6	1264	0.47	(0.09 to 0.85)			
Cancer[#]	Yes	1	218	0.46	(0 to 1.36)	2.87	(0.8 to 10.32)	0	4	214	1.87	(0.06 to 3.68)	4.94	(2.69 to 9.05)	0
	No*	2	2428	0.08	(0 to 0.19)				4	2428	0.16	(0 to 0.32)			
Previous VTE	Yes	1	786	0.13	(0 to 0.38)	1.30	(0.49 to 3.4)	0	3	786	0.38	(0 to 0.81)	1.82	(0.57 to 5.81)	0
	No*	2	1646	0.12	(0 to 0.29)				4	1642	0.24	(0 to 0.48)			
Decreased kidney function[^]	Yes	0	155	0.00	(0 to 0)	NA	NA	NA	0	155	0.00	(0 to 0)	NA	NA	NA
	No*	3	1918	0.16	(0 to 0.34)				6	1915	0.31	(0.06 to 0.56)			
Preexisting cardio-pulmonary disease[¥]	Yes	1	210	0.48	(0 to 1.41)	3.63	(0.34 to 38.16)	5.4	1	208	0.48	(0 to 1.42)	2.00	(0.12 to 34.32)	12.5
	No*	1	1242	0.08	(0 to 0.24)				5	1240	0.40	(0.05 to 0.75)			
Treatment	DOAC*	0	1508	0.00	(0 to 0)	3.21	(0.17 to 61.5)	0	1	1507	0.07	(0 to 0.2)	2.70	(0.89 to 8.16)	0
	LMWH or VKA	3	998	0.30	(0 to 0.64)				6	995	0.60	(0.12 to 1.08)			
Abnormal troponin[Ω]	Yes	0	147	0.00	(0 to 0)	0.77	(0.47 to 1.28)	0	1	147	0.68	(0 to 2.01)	2.80	(0.52 to 15.03)	0
	No*	1	1126	0.09	(0 to 0.26)				2	1125	0.18	(0 to 0.43)			
Abnormal (NT-pro)BNP[±]	Yes	0	107	0.00	(0 to 0)	0.95	(0.79 to 1.15)	0	0	107	0.00	(0 to 0)	0.92	(0.68 to 1.24)	0
	No*	1	1221	0.08	(0 to 0.24)				2	1220	0.16	(0 to 0.39)			
Signs of RV overload[§]	Yes	0	281	0.00	(0 to 0)	0.46	(0.13 to 1.7)	0	1	281	0.36	(0 to 1.06)	0.71	(0.45 to 1.11)	0
	No*	1	719	0.14	(0 to 0.41)				1	718	0.14	(0 to 0.41)			
RV dysfunction on TTE	Yes	0	8	0.00	(0 to 0)	NA	NA	NA	0	8	0.00	(0 to 0)	NA	NA	NA
	No*	0	401	0.00	(0 to 0)				0	399	0.00	(0 to 0)			
Symptoms	Incidental *	0	15	0.00	(0 to 0)	1.00	(0 to 1005.22)	0	0	15	0.00	(0 to 0)	1.00	(0 to 986.94)	0
	Symptomatic	3	1641	0.18	(0 to 0.39)				6	1640	0.37	(0.08 to 0.66)			
Age	18-40*	0	582	0.00	(0 to 0)				1	582	0.17	(0 to 0.51)			
	41-60	2	1086	0.18	(0 to 0.44)	1.28	(0.9 to 1.82)	0	2	1085	0.18	(0 to 0.44)	0.93	(0.47 to 1.84)	0
	61-80	1	896	0.11	(0 to 0.33)	1.14	(0.86 to 1.5)	0	5	893	0.56	(0.07 to 1.05)	1.25	(0.54 to 2.87)	0
	>81	0	99	0.00	(0 to 0)	NA	NA	0	0	99	0.00	(0 to 0)	0.84	(0.62 to 1.14)	0

I^2 was 0% and 0 for all proportion analysis * RR presents the ratio of the risk for an event for the exposure group to the risk for the non-exposure/reference group; non-exposure/reference group is marked with an asterisk # (1) Current diagnosis of cancer, (2) receiving treatment for cancer or (3) not receiving treatment for cancer and not in complete response ; ^Estimated Glomerular Filtration Rate < 60 ml/min; ¥Preexisting pulmonary disease was defined as a history of chronic obstructive pulmonary disease, asthma, or lung fibrosis, a preexisting cardiovascular disease, defined as any of coronary artery disease, heart failure, congenital heart disease, cardiomyopathy or rheumatic heart disease; ΩAbnormal troponin was defined as a troponin level >99th percentile according to local technique; ± NT-proBNP > 500 ng/L or BNP level >100 ng/L ; §Right ventricle/ left ventricle ratio >0.9 on computed tomography pulmonary angiogram or echocardiogram; Abbreviations: CI, confidence interval; DOAC direct oral anticoagulant; LMWH low molecular weight heparin; MB, major bleeding; NA not applicable; PI prediction interval; RR relative risk; RV right ventricle; TTE trans thoracic echocardiography; VKA vitamin K antagonist; VTE, venous thromboembolism.

Table S16: overall and subgroup analysis at 14- and 30-days for combined endpoint(i.e. combined endpoint of recurrent VTE, MB or mortality) : sensitivity analysis non-imputed data (discharge within 24 hours)

		Adverse events at 14 days							Adverse events at 30 days						
**subgroup variable**	**subgroup level**	**Events (n)**	**Patients (n)**	**%**	**(95%CI)**	**RR**	**(95%PI)**	**I2**	**Events (n)**	**Patients (n)**	**%**	**(95%CI)**	**RR**	**(95%PI)**	**I2**
overall		15	2660	0.56	(0.28 to 0.84)				32	2653	1.21	(0.79 to 1.63)			
Sex	Male*	9	1396	0.64	(0.22 to 1.06)	1.08	(0.48 to 2.43)	0	14	1393	1.01	(0.49 to 1.53)	1.39	(0.57 to 3.4)	7.3
	Female	6	1264	0.47	(0.09 to 0.85)				18	1260	1.43	(0.77 to 2.09)			
Cancer[#]	Yes	1	216	0.46	(0 to 1.37)	1.64	(0.7 to 3.89)	0	5	210	2.38	(0.32 to 4.44)	2.70	(1.4 to 5.21)	0
	No*	14	2426	0.58	(0.28 to 0.88)				27	2425	1.11	(0.69 to 1.53)			
Previous VTE	Yes	6	785	0.76	(0.15 to 1.37)	1.34	(0.54 to 3.32)	0	12	784	1.53	(0.67 to 2.39)	1.33	(0.64 to 2.75)	0
	No*	8	1643	0.49	(0.15 to 0.83)				17	1637	1.04	(0.55 to 1.53)			
Decreased kidney function[^]	Yes	0	155	0.00	(0 to 0)	NA	NA	NA	0	154	0.00	(0 to 0)	NA	NA	NA
	No*	12	1914	0.63	(0.28 to 0.98)				22	1909	1.15	(0.67 to 1.63)			
Preexisting cardio-pulmonary disease[¥]	Yes	2	209	0.96	(0 to 2.28)	4.57	(1.54 to 13.54)	0	2	206	0.97	(0 to 2.31)	2.54	(0.87 to 7.38)	0
	No*	4	1239	0.32	(0 to 0.64)				11	1235	0.89	(0.37 to 1.41)			
Treatment	DOAC*	8	1506	0.53	(0.16 to 0.9)	1.39	(0.75 to 2.56)	0	17	1504	1.13	(0.6 to 1.66)	1.51	(0.79 to 2.89)	0
	LMWH or VKA	7	996	0.70	(0.18 to 1.22)				14	991	1.41	(0.68 to 2.14)			
Abnormal troponin[Ω]	Yes	1	147	0.68	(0 to 2.01)	2.22	(0.61 to 8.16)	0	4	147	2.72	(0.09 to 5.35)	3.06	(1.24 to 7.53)	0
	No*	3	1125	0.27	(0 to 0.57)				9	1121	0.80	(0.28 to 1.32)			
Abnormal (NT-pro)BNP[±]	Yes	2	107	1.87	(0 to 4.44)	4.45	(1.75 to 11.28)	0	3	105	2.86	(0 to 6.05)	3.54	(1.56 to 8.02)	0
	No*	2	1220	0.16	(0 to 0.39)				8	1217	0.66	(0.21 to 1.11)			
Signs of RV overload[§]	Yes	5	280	1.79	(0.24 to 3.34)	2.66	(0.48 to 14.59)	2.1	8	280	2.86	(0.91 to 4.81)	2.19	(0.28 to 16.91)	14.1
	No*	2	719	0.28	(0 to 0.66)				5	718	0.70	(0.09 to 1.31)			
RV dysfunction on TTE	Yes	0	7	0.00	(0 to 0)	NA	NA	NA	0	7	0.00	(0 to 0)	NA	NA	NA
	No*	0	401	0.00	(0 to 0)				0	399	0.00	(0 to 0)			
Symptoms	Incidental *	0	15	0.00	(0 to 0)	1.00	(0 to 1005.22)	0	0	15	0.00	(0 to 0)	1.00	(0 to 986.94)	0
	Symptomatic	10	1641	0.61	(0.23 to 0.99)				20	1640	1.22	(0.69 to 1.75)			
Age	18-40*	3	580	0.52	(0 to 1.1)				8	580	1.38	(0.43 to 2.33)			
	41-60	8	1086	0.74	(0.23 to 1.25)	1.10	(0.66 to 1.84)	0	12	1084	1.11	(0.49 to 1.73)	0.82	(0.45 to 1.5)	0
	61-80	4	894	0.45	(0.01 to 0.89)	0.96	(0.48 to 1.94)	0	12	889	1.35	(0.59 to 2.11)	0.91	(0.61 to 1.37)	0
	>81	0	99	0.00	(0 to 0)	0.77	(0.52 to 1.15)	0	0	99	0.00	(0 to 0)	0.49	(0.31 to 0.77)	0

I^2 was 0% and 0 for all proportion analysis * RR presents the ratio of the risk for an event for the exposure group to the risk for the non-exposure/reference group; non-exposure/reference group is marked with an asterisk # (1) Current diagnosis of cancer, (2) receiving treatment for cancer or (3) not receiving treatment for cancer and not in complete response ; ^Estimated Glomerular Filtration Rate < 60 ml/min; ¥Preexisting pulmonary disease was defined as a history of chronic obstructive pulmonary disease, asthma, or lung fibrosis, a preexisting cardiovascular disease, defined as any of coronary artery disease, heart failure, congenital heart disease, cardiomyopathy or rheumatic heart disease; ΩAbnormal troponin was defined as a troponin level >99th percentile according to local technique; ± NT-proBNP > 500 ng/L or BNP level >100 ng/L ; §Right ventricle/ left ventricle ratio >0.9 on computed tomography pulmonary angiogram or echocardiogram; Abbreviations: CI, confidence interval; DOAC direct oral anticoagulant; LMWH low molecular weight heparin; MB, major bleeding; NA not applicable; PI prediction interval; RR relative risk; RV right ventricle; TTE trans thoracic echocardiography; VKA vitamin K antagonist; VTE, venous thromboembolism.

Table S17: overall and subgroup analysis at 14- and 30-days for recurrent VTE: sensitivity analysis non-imputed data (discharge within 24 hours)

		Recurrent VTE at 14 days							Recurrent VTE at 30 days						
**subgroup variable**	**subgroup level**	**Events (n)**	**Patients (n)**	**%**	**(95%CI)**	**RR**	**(95%PI)**	**I2**	**Events (n)**	**Patients (n)**	**%**	**(95%CI)**	**RR**	**(95%PI)**	**I2**
overall		9	2661	0.34	(0.12 to 0.56)				15	2654	0.57	(0.28 to 0.86)			
Sex	Male*	7	1397	0.50	(0.13 to 0.87)	0.74	(0.31 to 1.81)	0	10	1394	0.72	(0.28 to 1.16)	0.78	(0.36 to 1.71)	0
	Female	2	1264	0.16	(0 to 0.38)				5	1260	0.40	(0.05 to 0.75)			
Cancer[#]	Yes	0	217	0.00	(0 to 0)	0.71	(0.41 to 1.22)	0	0	211	0.00	(0 to 0)	0.61	(0.31 to 1.17)	0
	No*	9	2426	0.37	(0.13 to 0.61)				15	2425	0.62	(0.31 to 0.93)			
Previous VTE	Yes	5	785	0.64	(0.08 to 1.2)	1.40	(0.66 to 2.99)	0	9	784	1.15	(0.4 to 1.9)	2.34	(0.92 to 5.92)	0
	No*	3	1644	0.18	(0 to 0.39)				4	1639	0.24	(0 to 0.48)			
Decreased kidney function[^]	Yes	0	155	0.00	(0 to 0)	0.60	(0.27 to 1.33)	0	0	155	0.00	(0 to 0)	0.46	(0.21 to 1.02)	0
	No*	6	1915	0.31	(0.06 to 0.56)				9	1911	0.47	(0.16 to 0.78)			
Preexisting cardio-pulmonary disease[¥]	Yes	1	209	0.48	(0 to 1.42)	3.34	(1 to 11.13)	0	1	208	0.48	(0 to 1.42)	2.85	(0.98 to 8.3)	0
	No*	2	1241	0.16	(0 to 0.38)				4	1236	0.32	(0 to 0.64)			
Treatment	DOAC*	6	1508	0.40	(0.08 to 0.72)	0.79	(0.26 to 2.36)	0	10	1507	0.66	(0.25 to 1.07)	0.69	(0.12 to 3.8)	0
	LMWH or VKA	3	995	0.30	(0 to 0.64)				5	990	0.51	(0.07 to 0.95)			
Abnormal troponin[Ω]	Yes	0	147	0.00	(0 to 0)	0.87	(0.59 to 1.3)	0	1	146	0.68	(0 to 2.02)	2.64	(0.44 to 15.72)	0
	No*	1	1125	0.09	(0 to 0.26)				4	1124	0.36	(0.01 to 0.71)			
Abnormal (NT-pro)BNP[±]	Yes	0	107	0.00	(0 to 0)	NA	NA	NA	0	107	0.00	(0 to 0)	0.69	(0.4 to 1.19)	0
	No*	0	1220	0.00	(0 to 0)				3	1220	0.25	(0 to 0.53)			
Signs of RV overload[§]	Yes	4	281	1.42	(0.03 to 2.81)	4.24	(0.91 to 19.68)	0	5	280	1.79	(0.24 to 3.34)	2.85	(0.11 to 73.34)	17.3
	No*	0	718	0.00	(0 to 0)				1	718	0.14	(0 to 0.41)			
RV dysfunction on TTE	Yes	0	8	0.00	(0 to 0)	NA	NA	NA	NA	8	0.00	(0 to 0)	NA	NA	NA
	No*	0	401	0.00	(0 to 0)				0	400	0.00	(0 to 0)			
Symptoms	Incidental *	0	15	0.00	(0 to 0)	1.00	(0 to 1005.22)	0	0	15	0.00	(0 to 0)	1.00	(0 to 1005.22)	0
	Symptomatic	4	1638	0.24	(0 to 0.48)				7	1636	0.43	(0.11 to 0.75)			
Age	18-40*	3	582	0.52	(0 to 1.1)				4	581	0.69	(0.02 to 1.36)			
	41-60	4	1084	0.37	(0.01 to 0.73)	0.89	(0.53 to 1.5)	0	6	1084	0.55	(0.11 to 0.99)	0.93	(0.42 to 2.06)	0
	61-80	2	895	0.22	(0 to 0.53)	0.79	(0.45 to 1.39)	0	5	889	0.56	(0.07 to 1.05)	0.91	(0.67 to 1.25)	0
	>81	0	99	0.00	(0 to 0)	0.77	(0.52 to 1.15)	0	0	99	0.00	(0 to 0)	0.72	(0.45 to 1.17)	0

I^2 was 0% and 0 for all proportion analysis * RR presents the ratio of the risk for an event for the exposure group to the risk for the non-exposure/reference group; non-exposure/reference group is marked with an asterisk # (1) Current diagnosis of cancer, (2) receiving treatment for cancer or (3) not receiving treatment for cancer and not in complete response ; ^Estimated Glomerular Filtration Rate < 60 ml/min; ¥Preexisting pulmonary disease was defined as a history of chronic obstructive pulmonary disease, asthma, or lung fibrosis, a preexisting cardiovascular disease, defined as any of coronary artery disease, heart failure, congenital heart disease, cardiomyopathy or rheumatic heart disease; ΩAbnormal troponin was defined as a troponin level >99th percentile according to local technique; ± NT-proBNP > 500 ng/L or BNP level >100 ng/L ; §Right ventricle/ left ventricle ratio >0.9 on computed tomography pulmonary angiogram or echocardiogram; Abbreviations: CI, confidence interval; DOAC direct oral anticoagulant; LMWH low molecular weight heparin; MB, major bleeding; NA not applicable; PI prediction interval; RR relative risk; RV right ventricle; TTE trans thoracic echocardiography; VKA vitamin K antagonist; VTE, venous thromboembolism.

Table S18: overall and subgroup analysis at 14- and 30-days for major bleeding: sensitivity analysis non-imputed data (discharge within 24 hours)

		Major bleeding at 14 days							Major bleeding at 30 days						
**subgroup variable**	**subgroup level**	**Events (n)**	**Patients (n)**	**%**	**(95%CI)**	**RR**	**(95%PI)**	**I2**	**Events (n)**	**Patients (n)**	**%**	**(95%CI)**	**RR**	**(95%PI)**	**I2**
overall		5	2659	0.19	(0.03 to 0.35)				12	2648	0.45	(0.19 to 0.71)			
Sex	Male*	2	1396	0.14	(0 to 0.34)	1.28	(0.73 to 2.26)	0	3	1392	0.22	(0 to 0.46)	1.91	(1.11 to 3.3)	0
	Female	3	1263	0.24	(0 to 0.51)				9	1256	0.72	(0.25 to 1.19)			
Cancer[#]	Yes	1	216	0.46	(0 to 1.37)	2.13	(0.98 to 4.65)	0	2	208	0.96	(0 to 2.29)	2.88	(1.22 to 6.83)	0
	No*	4	2425	0.16	(0 to 0.32)				10	2422	0.41	(0.15 to 0.67)			
Previous VTE	Yes	0	784	0.00	(0 to 0)	0.59	(0.32 to 1.09)	0	1	781	0.13	(0 to 0.38)	0.48	(0.24 to 0.97)	0
	No*	5	1643	0.30	(0.03 to 0.57)				11	1636	0.67	(0.27 to 1.07)			
Decreased kidney function[^]	Yes	0	155	0.00	(0 to 0)	0.65	(0.36 to 1.18)	0	0	154	0.00	(0 to 0)	0.46	(0.23 to 0.94)	0
	No*	5	1913	0.26	(0.03 to 0.49)				10	1906	0.52	(0.2 to 0.84)			
Preexisting cardio-pulmonary disease[¥]	Yes	0	208	0.00	(0 to 0)	0.91	(0.69 to 1.2)	0	0	206	0.00	(0 to 0)	0.87	(0.56 to 1.34)	0
	No*	2	1239	0.16	(0 to 0.38)				4	1231	0.32	(0 to 0.64)			
Treatment	DOAC*	2	1506	0.13	(0 to 0.31)	1.71	(0.61 to 4.83)	0	6	1504	0.40	(0.08 to 0.72)	1.98	(0.74 to 5.34)	0
	LMWH or VKA	3	995	0.30	(0 to 0.64)				6	987	0.61	(0.13 to 1.09)			
Abnormal troponin[Ω]	Yes	1	147	0.68	(0 to 2.01)	3.33	(0.55 to 20)	0	2	146	1.37	(0 to 3.26)	2.34	(1.14 to 4.8)	0
	No*	1	1124	0.09	(0 to 0.26)				4	1119	0.36	(0.01 to 0.71)			
Abnormal (NT-pro)BNP[±]	Yes	2	107	1.87	(0 to 4.44)	4.99	(1.54 to 16.18)	0	3	105	2.86	(0 to 6.05)	4.56	(2.08 to 9.98)	0
	No*	1	1219	0.08	(0 to 0.24)				4	1216	0.33	(0.01 to 0.65)			
Signs of RV overload[§]	Yes	1	280	0.36	(0 to 1.06)	1.15	(0.05 to 25.05)	11.5	2	279	0.72	(0 to 1.71)	1.05	(0.43 to 2.6)	0
	No*	2	719	0.28	(0 to 0.66)				4	719	0.56	(0.02 to 1.1)			
RV dysfunction on TTE	Yes	0	7	0.00	(0 to 0)	0.00	0.00	0	0	7	0.00	(0 to 0)	0.00	0.00	0
	No*	0	401	0.00	(0 to 0)				0	400	0.00	(0 to 0)			
Symptoms	Incidental *	0	15	0.00	(0 to 0)	1.00	(0 to 1005.22)	0	0	15	0.00	(0 to 0)	1.00	(0 to 1005.22)	0
	Symptomatic	5	1640	0.30	(0.03 to 0.57)				10	1637	0.61	(0.23 to 0.99)			
Age	18-40*	0	580	0.00	(0 to 0)				3	579	0.52	(0 to 1.1)			
	41-60	3	1085	0.28	(0 to 0.59)	1.40	(0.87 to 2.26)	0	5	1084	0.46	(0.06 to 0.86)	0.89	(0.51 to 1.57)	0
	61-80	2	894	0.22	(0 to 0.53)	1.32	(0.9 to 1.93)	0	4	885	0.45	(0.01 to 0.89)	0.85	(0.54 to 1.32)	0
	>81	0	99	0.00	(0 to 0)	1.00	(1 to 1)	0	0	99	0.00	(0 to 0)	0.74	(0.52 to 1.04)	0

I^2 was 0% for all proportion analysis * RR presents the ratio of the risk for an event for the exposure group to the risk for the non-exposure/reference group; non-exposure/reference group is marked with an asterisk # (1) Current diagnosis of cancer, (2) receiving treatment for cancer or (3) not receiving treatment for cancer and not in complete response ; ^Estimated Glomerular Filtration Rate < 60 ml/min; ¥Preexisting pulmonary disease was defined as a history of chronic obstructive pulmonary disease, asthma, or lung fibrosis, a preexisting cardiovascular disease, defined as any of coronary artery disease, heart failure, congenital heart disease, cardiomyopathy or rheumatic heart disease; ΩAbnormal troponin was defined as a troponin level >99th percentile according to local technique; ± NT-proBNP > 500 ng/L or BNP level >100 ng/L ; §Right ventricle/ left ventricle ratio >0.9 on computed tomography pulmonary angiogram or echocardiogram; Abbreviations: CI, confidence interval; DOAC direct oral anticoagulant; LMWH low molecular weight heparin; MB, major bleeding; NA not applicable; PI prediction interval; RR relative risk; RV right ventricle; TTE trans thoracic echocardiography; VKA vitamin K antagonist; VTE, venous thromboembolism.

Echocardiography data main analysis

Table S19: echocardiography analysis at 14-days for mortality and all adverse events (i.e. combined endpoint of recurrent VTE, MB or mortality)

		Combined endpoint of VTE, MB or all-cause morality						All-cause mortality					
		Events (n)	Patients (n)	%	(95%CI)	RR	(95%PI)	Events (n)	Patients (n)	%	(95% CI)	RR	(95%PI)
RV dysfunction on TTE	Yes	0	29	1.67	(0 to 6.35)	0.71	(0.26 to 1.97)	0	30	0.00	(0 to 0)	NA	NA
	No*	5	1,144	0.40	(0.04 to 0.76)			0	1,147	0.00	(0 to 0)		

This Table presents the 14-day incidence of the combined endpoint of VTE, MB or all-cause mortality and the 30-day incidence of all-cause mortality. * RR presents the ratio of the risk for an event for the exposure group to the risk for the non-exposure/reference group; non-exposure/reference group is marked with an asterisk Abbreviations: CI, confidence interval; MB, major bleeding; RR relative risk; RV right ventricle; TTE trans thoracic echocardiography; VTE, venous thromboembolism.

Table S20: echocardiography analysis at 30-days for mortality and all adverse events (i.e. combined endpoint of recurrent VTE, MB or mortality)

		Combined endpoint of VTE, MB or all-cause morality						All-cause mortality					
		Events (n)	Patients (n)	%	(95%CI)	RR	(95%PI)	Events (n)	Patients (n)	%	(95% CI)	RR	(95%PI)
RV dysfunction on TTE	Yes	1	28	2.30	(0 to 7.81)	0.47	(0.15 to 1.5)	0	30	0.18	(0 to 1.7)	0.71	(0.41 to 1.24)
	No*	11	1,138	1.00	(0.42 to 1.58)			2	1,143	0.17	(0 to 0.41)		

This Table presents the 30-day incidence of the combined endpoint of VTE, MB or all-cause mortality and the 30-day incidence of all-cause mortality. * RR presents the ratio of the risk for an event for the exposure group to the risk for the non-exposure/reference group; non-exposure/reference group is marked with an asterisk Abbreviations: CI, confidence interval; MB, major bleeding; RR relative risk; RV right ventricle; TTE trans thoracic echocardiography; VTE, venous thromboembolism.

Figure S1: incidence of 30-day adverse events and mortality with 95% PIs versus age as a continuous variable

A) Incidence (%) of 30-day adverse events (i.e. combined endpoint of death, major bleeding or recurrent venous thromboembolism) and B) Incidence (%) of 30-day mortality versus age

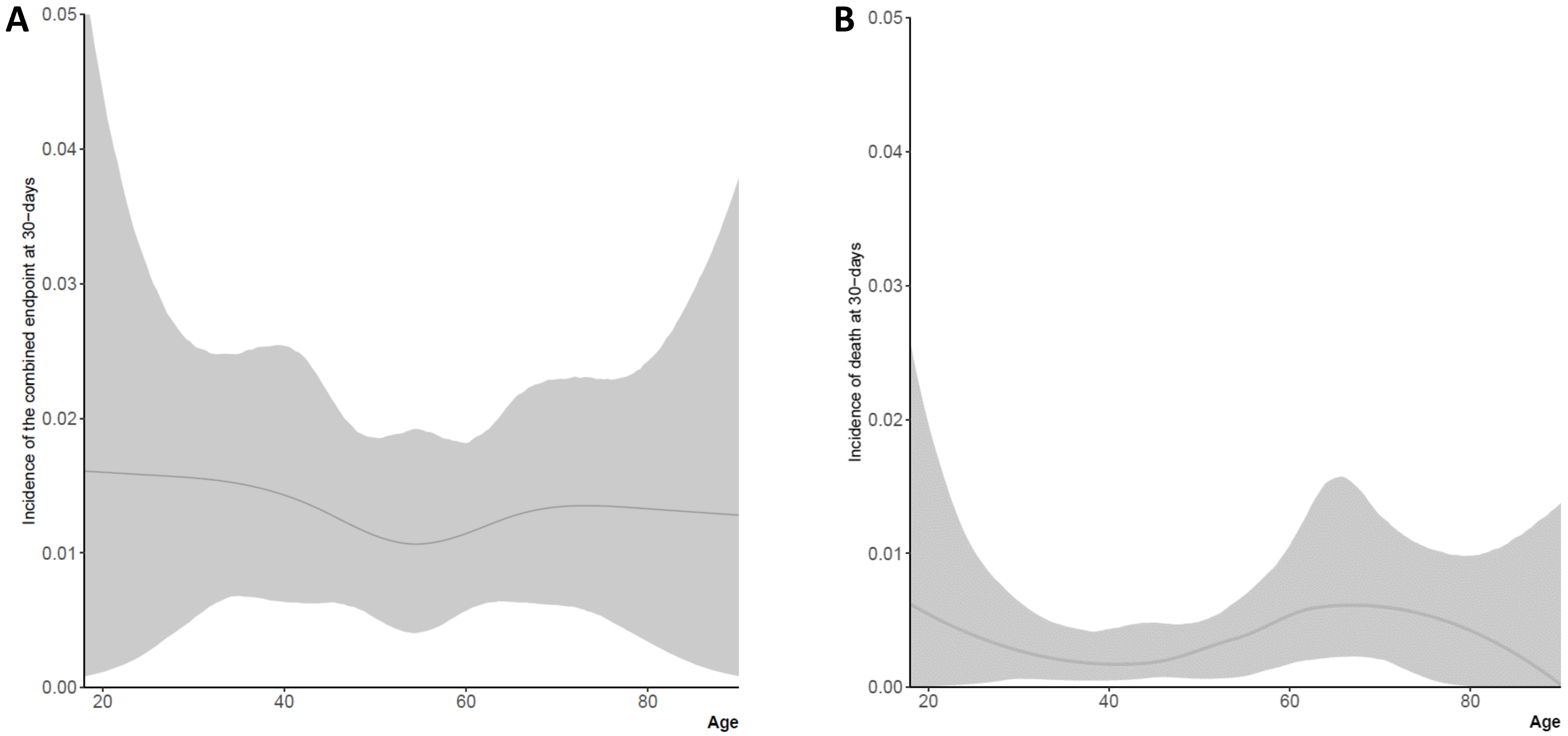

Figure S2: distribution of age in our cohort

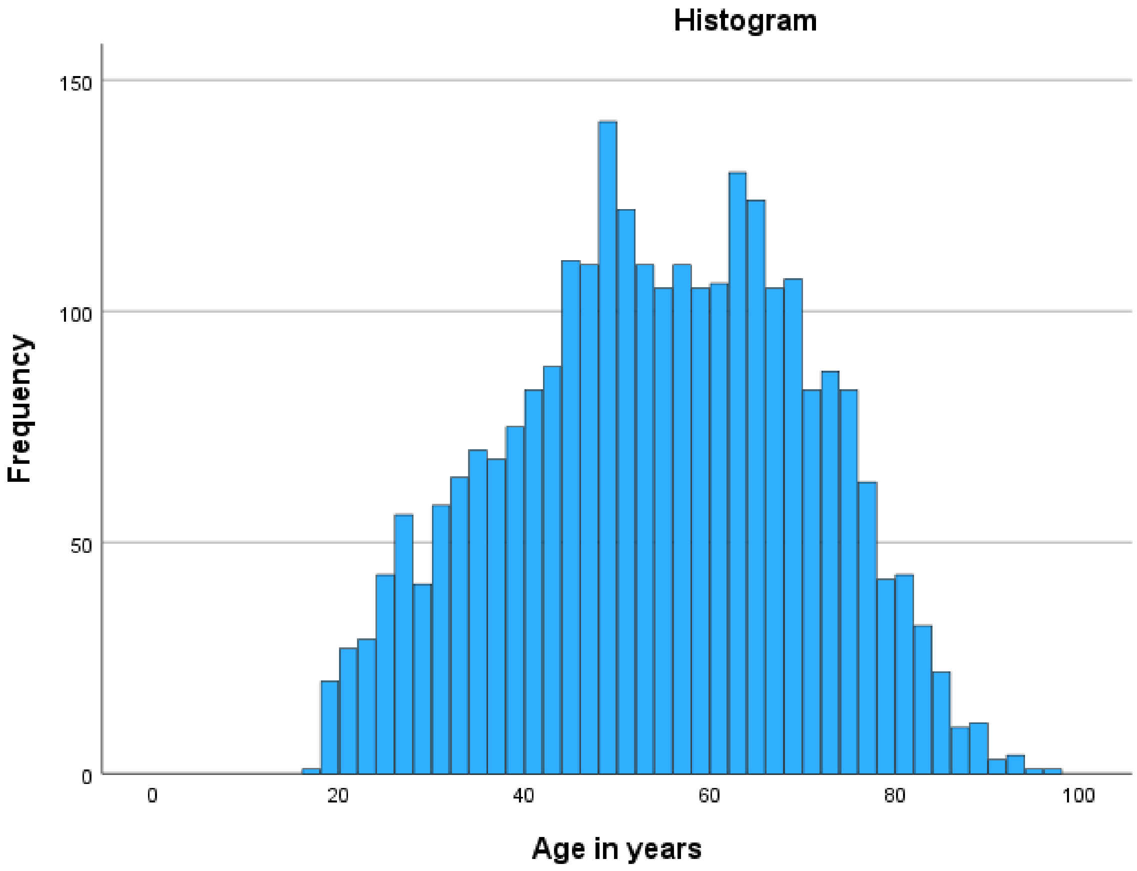

Appendix E: Missing data imputation

- All variables imputed using pmm (because of very rare binary variables, e.g., all outcomes)
- We included the following variables in our imputation model:
    - binary variables
        - sex
        - symp
        - treat.med.bin
        - mal
        - prevvte
        - renal60
        - carpulm_calc
        - tropoabn
        - ntprobnpabn500
        - rvlvratio9
        - echo.rvdys
        - auxiliarly variables imputation
            - immosur
            - estrogen
            - hr110
            - bpsyst100
            - resp30
            - sat90
            - location
        - vtefup.14
        - deathfup.14
        - mbfup.14
    - continuous variables
        - age
        - tropongml
        - creatinine

References

1. Le Gal G, Carrier M, Castellucci LA, et al. Development and implementation of common data elements for venous thromboembolism research: on behalf of SSC Subcommittee on official Communication from the SSC of the ISTH. *Journal of Thrombosis and Haemostasis* 2021; **19**(1): 297-303.
2. Konstantinides SV, Meyer G, Becattini C, et al. 2019 ESC Guidelines for the diagnosis and management of acute pulmonary embolism developed in collaboration with the European Respiratory Society (ERS). *The Task Force for the diagnosis and management of acute pulmonary embolism of the European Society of Cardiology (ESC)* 2019: 1901647.
3. Zondag W, Hiddinga BI, Crobach MJT, et al. Hestia criteria can discriminate high- from low-risk patients with pulmonary embolism. *European Respiratory Journal* 2013; **41**(3): 588-92.
4. Zondag W, Mos IC, Creemers-Schild D, et al. Outpatient treatment in patients with acute pulmonary embolism: the Hestia Study. *J Thromb Haemost* 2011; **9**(8): 1500-7.
5. den Exter PL, Zondag W, Klok FA, et al. Efficacy and Safety of Outpatient Treatment Based on the Hestia Clinical Decision Rule with or without N-Terminal Pro-Brain Natriuretic Peptide Testing in Patients with Acute Pulmonary Embolism. A Randomized Clinical Trial. *Am J Respir Crit Care Med* 2016; **194**(8): 998-1006.
6. Roy PM, Penaloza A, Hugli O, et al. Triaging acute pulmonary embolism for home treatment by Hestia or simplified PESI criteria: the HOME-PE randomized trial. *Eur Heart J* 2021; **42**(33): 3146-57.
7. Kline JA, Adler DH, Alanis N, et al. Monotherapy Anticoagulation to Expedite Home Treatment of Patients Diagnosed With Venous Thromboembolism in the Emergency Department: A Pragmatic Effectiveness Trial. *Circ Cardiovasc Qual Outcomes* 2021; **14**(7): e007600.
8. Barco S, Schmidtmann I, Ageno W, et al. Early discharge and home treatment of patients with low-risk pulmonary embolism with the oral factor Xa inhibitor rivaroxaban: an international multicentre single-arm clinical trial. *Eur Heart J* 2020; **41**(4): 509-18.
9. Kabrhel C, Rosovsky R, Baugh C, et al. Multicenter Implementation of a Novel Management Protocol Increases the Outpatient Treatment of Pulmonary Embolism and Deep Vein Thrombosis. *Acad Emerg Med* 2019; **26**(6): 657-69.
10. Otero R, Uresandi F, Jiménez D, et al. Home treatment in pulmonary embolism. *Thromb Res* 2010; **126**(1): e1-5.
11. Font C, Carmona-Bayonas A, Fernández-Martinez A, et al. Outpatient management of pulmonary embolism in cancer: data on a prospective cohort of 138 consecutive patients. *J Natl Compr Canc Netw* 2014; **12**(3): 365-73.
12. Vinson DR, Mark DG, Chettipally UK, et al. Increasing Safe Outpatient Management of Emergency Department Patients With Pulmonary Embolism: A Controlled Pragmatic Trial. *Ann Intern Med* 2018; **169**(12): 855-65.
13. Bledsoe JR, Woller SC, Stevens SM, et al. Management of Low-Risk Pulmonary Embolism Patients Without Hospitalization: The Low-Risk Pulmonary Embolism Prospective Management Study. *Chest* 2018; **154**(2): 249-56.
